# Unsupervised Multi-Omics Data Integration Methods: A Comprehensive Review

**DOI:** 10.3389/fgene.2022.854752

**Published:** 2022-03-22

**Authors:** Nasim Vahabi, George Michailidis

**Affiliations:** Informatics Institute, University of Florida, Gainesville, FL, United States

**Keywords:** multi-omics, unsupervised integration, data-ensemble, model-ensemble, sequential analysis, clustering method, network analysis

## Abstract

Through the developments of Omics technologies and dissemination of large-scale datasets, such as those from The Cancer Genome Atlas, Alzheimer’s Disease Neuroimaging Initiative, and Genotype-Tissue Expression, it is becoming increasingly possible to study complex biological processes and disease mechanisms more holistically. However, to obtain a comprehensive view of these complex systems, it is crucial to integrate data across various Omics modalities, and also leverage external knowledge available in biological databases. This review aims to provide an overview of multi-Omics data integration methods with different statistical approaches, focusing on *unsupervised learning* tasks, including disease onset prediction, biomarker discovery, disease subtyping, module discovery, and network/pathway analysis. We also briefly review feature selection methods, multi-Omics data sets, and resources/tools that constitute critical components for carrying out the integration.

## Introduction

With the development of multi-Omics initiatives (e.g., The Cancer Genome Atlas (TCGA) www.genome.gov/Funded-Programs-Projects/Cancer-Genome-Atlas, International Cancer Genome Consortium (ICGC) dcc.icgc.org/, and Genotype-Tissue Expression (GTEx) gtexportal.org/home/), several collections of Omics data (epigenome, genome, transcriptome, proteome, and metabolome) have become available to the biomedical community. Moreover, curated databases for Omics interactions (e.g., DoRiNA, a database of RNA interactions in post-transcriptional regulation, dorina.mdc-berlin.de/), and molecular pathways (e.g., Kyoto Encyclopedia of Genes and Genomes (KEGG) www.genome.jp/kegg/, Reactome reactome.org/, and functional protein association networks (STRING) string-db.org/) are also available to incorporate known biological information in the Omics analysis. Environmental/clinical features are external sources of influence that can play a key role in the development of complex diseases (Chakraborty et al., 2018). Incorporating known biological information (a detailed list of databases/resources is presented in Supplementary Table S1) is particularly important since the presence of many more features than available samples (high-dimensionality) pose a critical challenge to almost all Omics analysis methods. Note that human genomes are regulated at multiple levels, which are captured by different genomic assays, and also environmental/clinical factors. Further, these factors exhibit intricate interdependencies; for example, DNA methylation is known to affect the phenotypic outcome of genetic variation and offers highly complementary information on transcriptional silencing and gene imprinting. However, the identification of causal relationships is still very much a work in progress. Therefore, a coherent biological model of complex diseases would only be possible if the various layers of Omics regulations, environmental/clinical factors, and their relationships are considered. Interconnections and heterogeneity are other challenges in understanding the complex nature of diseases and their key biomarkers.

There have been various attempts to address these issues. The terms *supervised* and *unsupervised* are often used to describe different approaches to data integration. Supervised methods train a model using labeled training data with known outcome variables (such as disease status, exposure to a specific environmental factor, and survival time). In contrast, unsupervised data integration consists of a class of methods that make inferences and find patterns in input data sets without labeled outcome variables (such as normal/disease status, benign/tumor tissue, and early/late-stage progression). Unsupervised multi-Omics approaches typically aim to classify (e.g., disease and sample subtype) and discover biomarkers/modules (such as prioritize genes associated with a disease). There might be multiple outcome variables (such as time-to-cure, or cancer-stage) which are mostly considered one-by-one in the available methods (instead of using multivariate models). Note that multiple Omics data often contain missing values, an issue particularly common for individuals with measurements by selected Omics modalities. Imputation is a typical solution to infer the missing values, see (Song et al., 2020) for an overview of the available multi-Omics imputation methods. Most of the supervised multi-Omics methods/tools require “matched samples” (where multiple types of Omics data are measured on the same subject/patient). For the remainder of the paper, we consider that samples are matched unless otherwise is stated. Last but not least, the molecules and Omics modalities involved in a biological process are usually correlated, and it is shown that most of the major biological processes are only affected by a small set of features (Wang et al., 2014). Thus, different feature selection methods have been introduced to address this issue and decrease computational complexity (for a comprehensive review of feature selection methods refer to Supplementary Section S2).

In the sequel, we review key unsupervised multi-Omics data integration approaches and summarize the state-of-the-art of statistical models and related topics, including an overview of different Omics data and sources. Existing reviews on the topic of multi-Omics data integration are narrowly focused, such as on a specific statistical approach (e.g., network analysis or clustering) or in a specific field (e.g., machine learning methods in oncology (Nicora et al., 2020)). On the contrary, we provide a comprehensive list of key unsupervised multi-Omics data integration methods leveraging a diverse set of statistical methods and biological objectives. Note that we furnish technical details for a selective list of methods that have been more widely adopted in applications. The remainder of the paper is organized as follows: In *Multi-Omics Data*, we briefly review the nature of multi-Omics data. In *Unsupervised Multi-Omics Data Integration Methods*, we provide our categorization of unsupervised multi-Omics data integration methods followed by detailed descriptions and case studies of selected ones in each of the proposed categories. We conclude with some remarks and directions for future work. More detailed information, including technical descriptions, formulas/algorithms, and additional illustrative case studies are provided in the Appendix due to space limitations. Further, a comprehensive review of multi-Omics data definition (Supplementary Section S1) and feature selection methods (Supplementary Section S2) is provided in the Supplement.

## Multi-Omics Data

**FIGURE 1:**
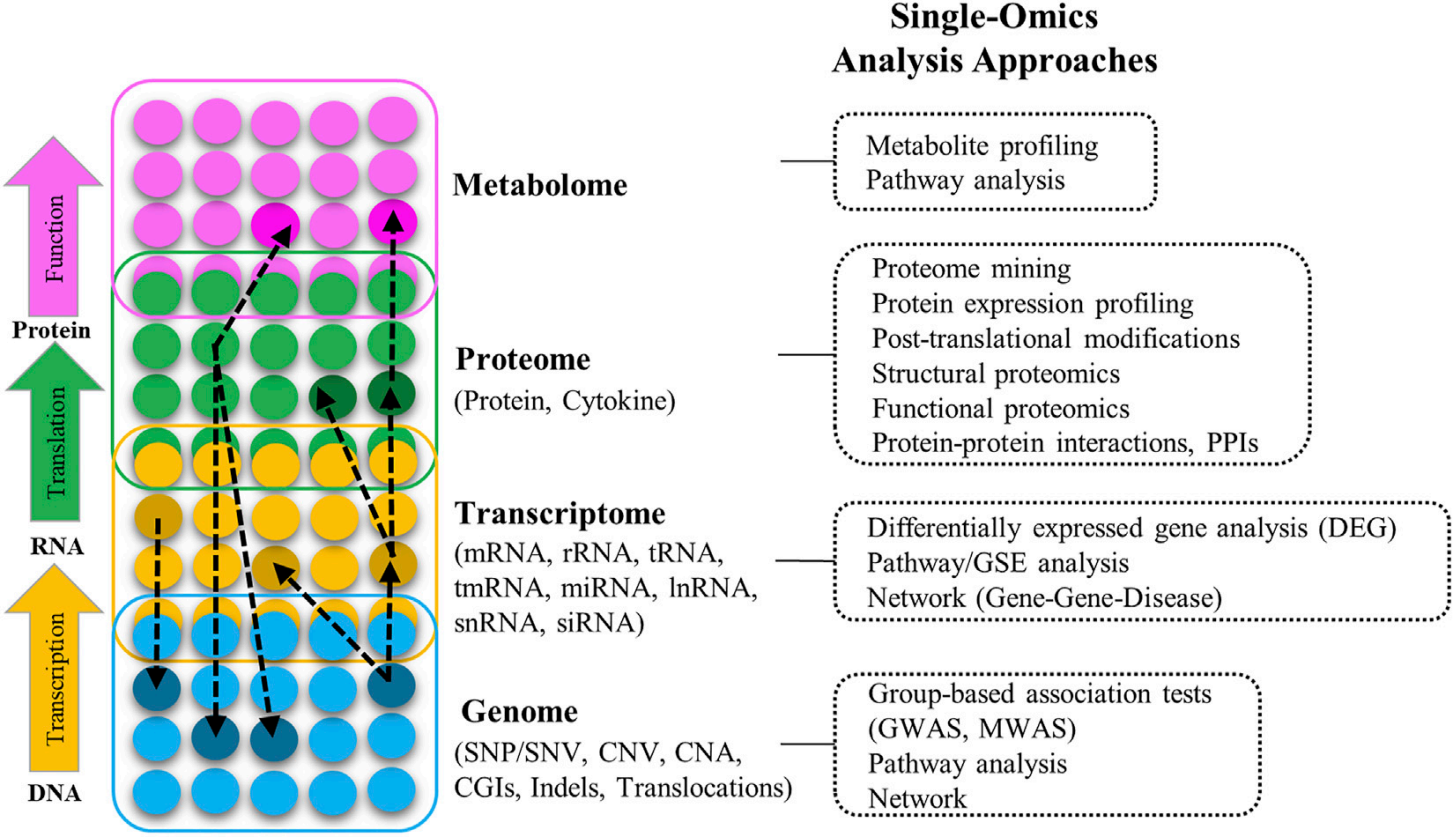
Different layers of multi-Omics data (genome, transcriptome, proteome, metabolome), the interactions between them (black dashed-arrow), types of the Omics features in each layer, and different approaches to analyze Omics data in different layers (SNP: Single nucleotide polymorphism, SNV: Single nucleotide variation, CNV: Copy number variation, CAN: Copy number alternation, CGIs: CpG islands, Indels: Insertion and deletion, GWAS: Genome-wide association study, MWAS: Methylation-wide association study, RNA: Ribonucleic acid, mRNA: Messenger RNA, rRNA: Ribosomal RNA, tRNA: Transfer RNA, tmRNA: Transfer-messenger RNA, miRNA: Micro RNA, lncRNA: Long-noncoding RNA, snRNA: Small nuclear RNA, siRNA: Small interfering RNA, GSE: Gene-set enrichment).

The term Omics refers to the collective characterization and quantification of biomolecules that are involved in the structure, function, and dynamics of organisms and biological processes. Figure 1 provides an overview of the molecular arrangement of key Omics modalities, potential interactions between and within them, the types of features available in each Omics layer, and the different approaches to their analysis. A full introduction to different Omics data is beyond the aim of this article; for detailed information and definition of Omics modalities, and a list of multiple Omics public data sources/repositories, refer to Supplementary Section S1 (including Supplementary Table S1). For a comprehensive overview of Omics modalities, background, technologies, and resources refer to (Gligorijević et al., 2016; Sun and Hu, 2016; Manzoni et al., 2018).

## Unsupervised Multi-Omics Data Integration Methods

**FIGURE 2:**
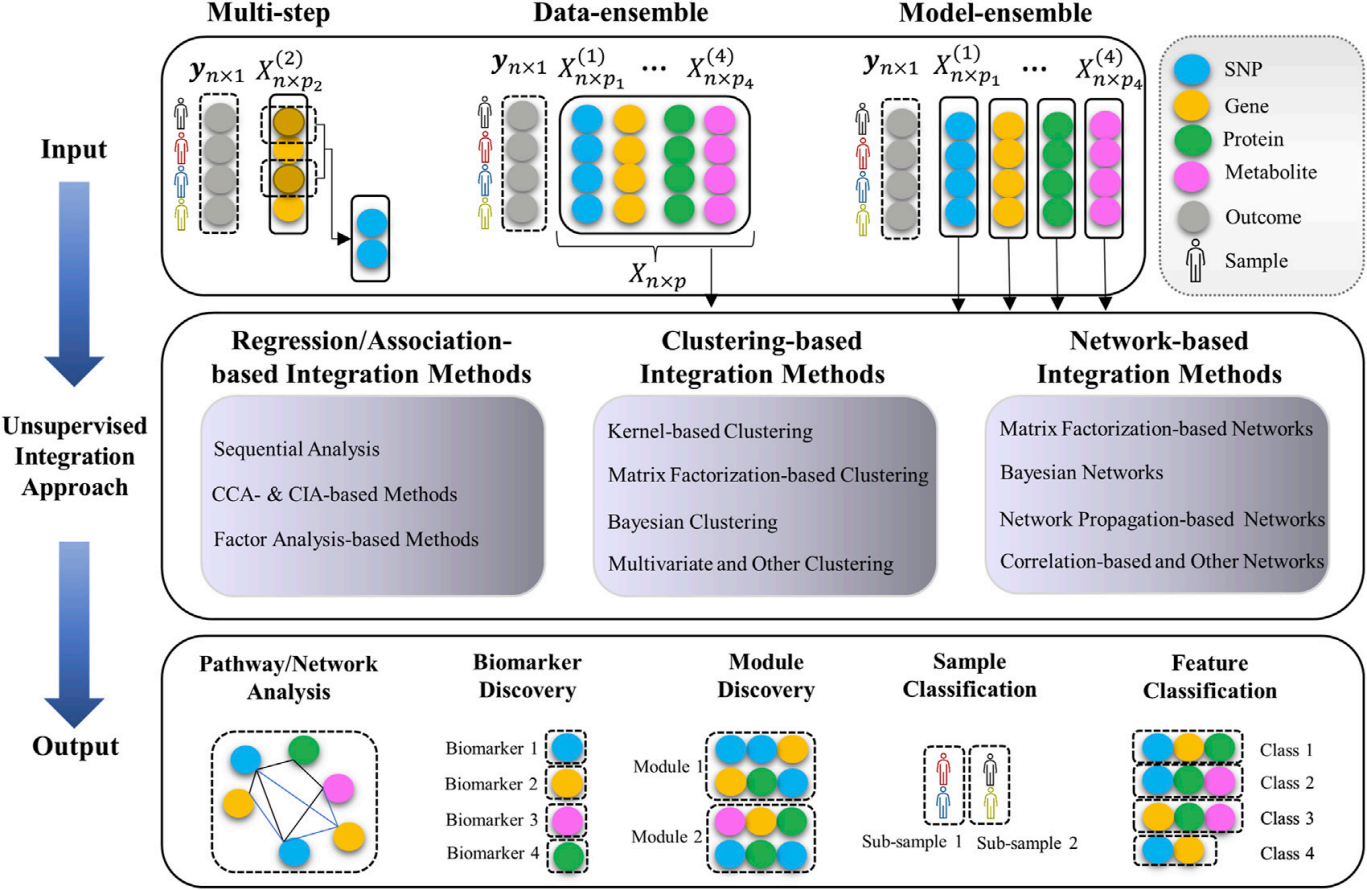
Unsupervised multi-omics data integration pipeline (input data, integration methods, and output). Data-ensemble methods concatenate the multi-Omics data from different molecular layers to a single matrix as the input data. Model-ensemble methods analyze each Omics data independently and then ensemble/fuse the results to construct an integrative analysis. A “module” is a combination of different Omics markers with similar functions or associations regarding the underlying outcome. A “class” is a group of Omics markers that have the same effect on the outcome. A “sub-sample” is a group of biological samples (e.g., human, animal, plant) with the same behavior regarding the underlying outcome. X and indicate the features and outcome variable, respectively. n and $p_{j}$ show the sample size and the number of the Omics features in the $j^{th}$ Omics type.

Categorizing the multi-Omics data integration methods is not a trivial task. There is a huge list of diverse methodologies with different objectives. One way to systematically categorize these methods is to consider their underlying statistical strategies, their biological objective, and the way they handle and treat multiple Omics datatypes. For instance, some methods (so-called “data-ensemble”) concatenate the multi-Omics data from different molecular layers to a single matrix as the input data (see Figure 2). Whereas the so-called “model-ensemble” approaches analyze each Omics data independently and then ensemble/fuse the results to construct an integrative analysis (see Figure 2).

We categorize the integration methods into the following three comprehensive categories: 1) *Regression/Association-based* Methods, 2) *Clustering-based* Methods, and 3) *Network-based* Methods. In each category, we group the methods based on their statistical approaches (see Table 1). Each of the methods will also be assigned to one of the following “macro” categories: (A) Multi-step and Sequential Analysis (MS-SA), (B) Data-ensemble (DatE), and (C) Model-ensemble (ModE) (see Figure 2 and Tables 2-4). “DatE” refers to methods that typically concatenate the multi-Omics data from different molecular layers to a single data matrix and consider that as the analysis input. Whereas the so-called “ModE” approaches analyze each Omics data independently and then ensemble/fuse the results to construct an integrative analysis. Table 1 shows the (high-level) list of the key methods we aim to review, with details provided in the proceeding sub-sections.

**TABLE 1:** High-level: Unsupervised multi-Omics data integration methods.

Category	Approach	Key methods
Regression/Association-based	Sequential Analysis	CNAMet (2011), MEMo (2012), iPAC (2013)
Integration Methods	CCA- and CIA-based Methods	Sparse MCCA (2009), BCCA (2013), MCIA (2014), sMCIA (2020)
(Refer to Table 2 for low-level details)	Factor Analysis-based Methods	Joint Bayesian Factor (2014), MOFA (2018), BayRel (2020)
Clustering-based	Kernel-based Clustering Methods	L-MKKM (2014), SNF (2014), rMKL-LPP (2015), WSNF (2016), mixKernel (2018), DSSF (2018), ANF (2018), NEMO (2019), ab-SNF (2019), MvNE (2020), INF (2020), SmSPK (2020), PAMOGK (2020)
Integration Methods	Matrix Factorization-based Clustering Methods	iCluster (2009), jNMF (2012), iClusterPlus (2013), FA (2013), moCluster (2016), JIVE (2016), iNMF (2016), PFA (2017), IS -means (2017), MOGSA (2019), SCFA (2020)
(Refer to Table 3 for low-level details)	Bayesian Clustering Methods	TMD (2010), PARADIGM (2010), PSDF (2011), MDI (2012), BCC (2013), LRAcluster (2015)
	Multivariate and Other Clustering Methods	COCA (2014), iPF (2015), Clusternomics (2017), PINS (2017), iDRW (2018), PINSPlus (2019), Subtype-GAN (2021)
Network-based	Matrix Factorization-based Networks	CMF (2008), NBS (2013), DFMF 2014), FUSENET (2015), Medusa (2016), MAE (2019), DisoFun (2020), IMCDriver (2021), RAIMC (2021)
Integration Methods	Bayesian Networks	PARADIGM (2010), CONEXIC (2010)
(Refer to Table 4 for low-level details)	Network Propagation-based Networks	GeneticInterPred (2010), RWRM (2012), TieDIE (2013), SNF (2014), HotNet2 (2015), NetICS (2018), RWR-M (2019), RWR-MH (2019), MSNE (2020), RWRF (2021)
	Correlation-based and Other Networks	WGCNA (2008), GGM (2011), GEM (2013), DBN (2015), Lemon-Tree (2015), TransNet (2018)

**TABLE 2:** Low-level: Regression/Association-based unsupervised integration methods.

Approach	Method	Macro category*	Author	Objective	Omics data**	Software***
Sequential Analysis	• CNAMet	MS-SA	Louhimo and Hautaniemi, (2011)	Biomarker-prediction	CNV, DM, GE	• *CNAmet* (http://csbi.ltdk.helsinki.fi/CNAmet )
	• MEMo (Mutual Exclusivity Modules)	MS-SA	Ciriello et al. (2012)	Module-discovery	CNA, GE	• JAVA code (http://cbio.mskcc.org/memo)
	• iPAC (in-trans Process Associated and cis-Correlated)	MS-SA	Aure et al. (2013)	Biomarker-prediction	CNV, GE	• *-*
CCA & CIA	• Sparse MCCA (Sparse Multiple Canonical Correlation Analysis)	DatE	Witten and Tibshirani, (2009)	Disease insight, Hotspot-detection	GE, CNV	• *PMA* (https://cran.r-project.org/web/packages/PMA/index.html)
	• BCCA (Bayesian Canonical Correlation Analysis)	DatE	Klami et al. (2013)	Disease insight	Any Omics	• *CCAGFA* (https://cran.r-project.org/web/packages/CCAGFA/index.html)
	• MCIA (Multiple Co-Inertia Analysis)	DatE	Meng et al. (2014)	Disease-subtyping, Biomarker-prediction	GE, PE	• *omicade4* (https://www.bioconductor.org/packages/release/bioc/html/omicade4.html)
						• *ade4* (https://cran.r-project.org/web/packages/ade4/index.html)
	• sMCIA (sparse Multiple Co-Inertia Analysis)	DatE	Min and Long, (2020)	Biomarker-prediction	Any Omics	• *pmCIA* (https://www.med.upenn.edu/long-lab/software.html)
Factor Analysis	• Joint Bayesian Factor	DatE	Ray et al. (2014)	Biomarker-prediction	CNV, DM, GE	• Matlab code (https://sites.google.com/site/jointgenomics/)
	• MOFA (Multi-Omics Factor Analysis)	DatE	Argelaguet et al. (2018)	Biomarker-prediction	Any Omics	• *MOFAtools*
						(https://github.com/bioFAM/MOFA)
	• BayRel (Bayesian Relational learning)	DatE	Hajiramezanali et al. (2020)	Biomarker-prediction	Any Omics	• TensorFlow (https://github.com/ehsanhajiramezanali/BayReL)

*Macro categories include (A) Multi-step and Sequential Analysis (MS-SA), (B) Data-ensemble (DatE), (C) Model-ensemble (ModE). ** CNV: copy number variation, DM: DNA methylation, GE: gene expression, PE: Protein expression. ***R packages, unless otherwise stated.

### Regression/Association-Based Integration Methods

One of the basic strategies for multi-Omics data integration is identifying marginal associations/correlations between different Omics layers. Sequential analysis is an example of this strategy where a sequence of statistical tests and models are applied to narrow down the list of features in one Omics layer (mostly genes) based on their relationship with features in other Omics layers (mostly CAN, genotypes, and DNA methylation). Multivariate analysis (such as CCA, CIA, and factor analysis) is another popular approach for multi-Omics data integration due to its flexibility in accepting multiple matrices as input data. The kernel-based method gives an excellent opportunity to work with lower space similarity kernels (such as patient-patient similarity, gene-gene similarity) instead of the original (raw) Omics data. We grouped *multi-Omics unsupervised regression-based* methods into three distinct categories based on their statistical approaches, including sequential analysis, CCA- and CIA-based, and factor analysis-based methods (see Table 2 for complementary details for each method).

#### Sequential Analysis

**CNAmet** (Louhimo and Hautaniemi, 2011) is a biomarker-discovery correlation-based method. It consists of two main steps; first, weights are calculated for each gene connecting it to DNA methylation and copy number variation (CNV). Second, each gene’s weights are combined and tested (using a corrected *p*-value *via* permutation) to calculate a global score for each gene. These scores help identify whether a gene is hypomethylated (and upregulated) or hypermethylated (and downregulated). The main hypothesis is that amplified copy number and hypomethylation result in gene upregulation. **iPAC** (in-trans Process Associated and cis-Correlated) (Aure et al., 2013) is an unsupervised, integrative method based on mRNA, and CNV aims to identify the *cis*-regulated genes. It also uses a sequence of statistical tests to narrow down the list of cancer driver genes. In summary, it takes the matrix of all the genes as the input, first filters the genes based on aberration frequency (>10%), then filters the remaining genes based on *in-cis* correlation (>0.6), and finally checks the *in-trans* functionality for the remaining ones that make the final set of gens. **MEMo** (Mutual Exclusivity Modules) (Ciriello et al., 2012) is a module-discovery method to find a set of genes that exhibit the same genetic alternation among patients. First, it gives a score to each gene and makes a binary-event-matrix based on these scores where its elements are either “1” indicating that the gene is significantly altered or “0” otherwise. Subsequently, it builds a network from genes involved in the same molecular pathway (using curated and nun-curated sources of biological information/interactions). The final step collects the genomic events within this network that show a significant level of mutual exclusivity *via* a permutation test.

**Illustrative Case-studies: CNAmet** is applied to a cohort of glioblastoma multiforme (GBM) patients from TCGA to find synergistically regulated genes by DNA methylation CNV. It could identify this synergistic effect on well-known oncogenes, including *MDM2*, *EGFR,* and *PDGFRA*. CNAmet also showed that GBM patients with hypomethylated (upregulated) *EGFR* had a better prognosis than patients with amplified *EGFR*. **iPAC** is applied to a cohort of breast carcinoma patients. It identified a list of significant genes, including *ERBB2*, *MAP3K7*, *MDM4*, *FGFR1*, *CCND1*, and *FADD,* which are well-known cancer-associated genes. It also included some less appreciated genes such as *ATAD2*, *TPD52,* and *PPM1D*, which were reported as cancer genes in previous independent studies (Choschzick et al., 2010; Lambros et al., 2010). iPAC could also identify several novel genes such as *MTL5* that can affect multiple proteins/enzymes *via* its negative correlation with the MT (metallothionein) family of proteins and metal-binding ability.

#### CCA- and CIA-Based Methods

**CCA-based methods** (Dolédec and Chessel, 1994) can be applied for module identification, feature selection, and classification in high-dimensional multi-Omics data. Due to the high dimensionality of Omics data, standard CCA cannot be employed directly. Therefore, there have been several extensions of CCA for more than two datasets ( B≥3) and/or high dimensional data in an unsupervised setting. **Sparse MCCA** (Sparse Multiple Canonical Correlation Analysis) (Witten and Tibshirani, 2009) is a sample-subtyping method applicable for more than two data types. It aims to find sparse components (linear combination of the features) by maximizing the following objective function ( $X^{\left(b\right)}s$ are standardized beforehand):

$$argmax_{w^{\left(b\right)}}\sum_{b}w\prime^{\left(b\right)}X\prime^{\left(b\right)}X^{\left(b\right)}w^{\left(b\right)},s.t.\|w^{\left(b\right)}{\|}^{2}\leq 1,P_{b}\left(w^{\left(b\right)}\right)\leq c_{b},$$

where $P_{b}$ is a convex penalty function, such as lasso or fused lasso, and $c_{b}$ is a tuning parameter for data type b ( b=1,…,B ). Two main disadvantages of CCA-based models are (1) they are not capable of handling non-linearity in data, which most of the time is the case in real-world data, and (2) they cannot fully take into account the structural information between and within Omics data (such as gene-gene interaction and PPIs).

**CIA-based** methods (Dolédec and Chessel, 1994; Dray et al., 2003) is another approach to find the low-dimensional components in two-table data settings where $X=\left[X_{n\times p_{1}}^{\left(1\right)}|X_{n\times p_{2}}^{\left(2\right)}\right]$ . This method was first introduced in ecology to link species abundance with environmental features. Orthonormal directions ( u and v **)** are computed by maximizing the covariance between the data tables:

$$argmax_{u,v}\;\;u\prime X^{\left(1\right)}\prime X^{\left(2\right)}v,s.t.\|u\|=\|v\|=1.$$

CIA can be considered as a variation of CCA (Sankaran and Holmes, 2019); the only difference is that in CIA the norm constraint ( ‖u‖=‖v‖=1 ) is directly applied on the orthonormal directions ( u and v **)**. **MCIA** (Multiple Co-Inertia Analysis) (Meng et al., 2014) is the extension of the CIA for the analysis of more than two data tables. **sMCIA** (sparse Multiple Co-Inertia Analysis) (Min and Long, 2020) is a sparse version of MCIA *via* imposing a sparsity constraint on the transformed direction vectors.

**Illustrative Case-studies: Sparse MCCA** is applied on a diffuse large B-cell lymphoma dataset to assess the relationships (such as co-amplification and codeletion) between copy number changes in genome regions on separate chromosomes (Meng et al., 2014). The results showed a complex relationship between CAN in different chromosomes. **MCIA** is applied to gene and protein expression for NCI-60 cancer cell lines from different tissues. Results showed that different cell lines were differentiated based on their tissue of origin. That is, cell line-specific features can help improve prediction and biomarker identification. In the 2nd application (Meng et al., 2014), MCIA is applied to a cohort of ovarian cancer patients, including mRNA expression data obtained from microarray and NGS. It identified four known subtypes of ovarian cancer (proliferative, immunoreactive, mesenchymal, and differentiated) along with its first two directions (components). Moreover, gene expression analysis in each component showed the capability of MCIA to detect disease subtype-specific markers.

#### Factor Analysis-Based Methods

**MOFA** (Multi-Omics Factor Analysis) (Argelaguet et al., 2018) is an unsupervised multi-Omics integration method that aims to detect the sources of variation (both technical and biological) in datasets *via* latent factors. It first decomposes each Omics data ( $X^{\left(b\right)}$ ) as follows:

$$X^{\left(b\right)}=ZW^{\left(b\right)}+\varepsilon^{\left(b\right)},b=1,\ldots ,B$$

where Z indicates the factor-matrix which is common for all data types and $W^{\left(b\right)}$ is a matrix of weights for datatype b , and $\varepsilon^{\left(b\right)}$ is the residual (or noise) for datatype b . It then—following the Bayesian framework—assigns a prior distribution for Z , $W^{\left(b\right)}$ , and parameters of the noise term. MOFA then applies a two-step regularization on the weight matrices to deal with the high dimensionality of multi-Omics data. it first identifies which factor is more active in which datatype (Omics type) and then applies a feature-wise sparsity to find a smaller set of features with active weights. These latent factors can serve as an input for further downstream analysis, including sample classification and missing data imputation. The most important advantages of MOFA are its interpretability, the ability to visualize samples in the factor space, and the capability of handling missing data and data with different distributions.

**Illustrative Case-studies: MOFA** is applied to a cohort of patients with chronic lymphocytic leukemia (CLL) to integrate mRNA expression, DNA methylation, somatic mutation, and drug response. It identified two important (already-known) markers, including the *IGHV* gene (immunoglobulin heavy-chain variable) and trisomy of chromosome 12. However, MOFA could find a more comprehensive and complex sub-structure for *IGHV* and connect it with multiple Omics, including changes in mRNA expression (*LPL*, *PLD1*, *ADAM29*), DNA methylation (cg17479716, cg19358877, cg26615224), and with drugs (tamatinib, dasatinib, AZD7762) that target kinases in the B-cell receptor pathway (Argelaguet et al., 2018). These changes in mRNA expression and DNA methylation were previously connected to IGHV in different independent studies (Plesingerova et al., 2017). Interestingly, *IGHV* and trisomy of chromosome 12 explained only <20% of the variation in CLL patients, indicating the presence of other factors and sources of heterogeneity. Therefore, they could find the oxidative stress pathway (with *HSP* family of proteins as the top-weighted genes) as one of the critical drivers which was previously underappreciated in the context of CLL. The results (factors) of MOFA are then used in a Cox-PH regression model and could predict the time to the next treatment with a reasonably high prediction accuracy (C-index∼75%). In the second and the third applications, MOFA is used to analyze Ustekinumab (UST) drug-response (Verstockt et al., 2019) and mESCs (mouse embryonic stem cells) multi-omics data (Argelaguet et al., 2018) to identify predictive factors (a combination of different Omics data).

### Clustering-Based Integration Methods

Multi-Omics clustering methods enable the discovery of molecular subtypes, disease subtypes, and patterns/modules. These methods mostly aim to find a subgroup of features/samples that have similar functions/patterns. We grouped *unsupervised multi-Omics clustering* methods into four distinct categories based on their statistical approaches, including 1) kernel-based, 2) (non-negative) matrix factorization-based-based, 3) Bayesian, and 4) multivariate and other clustering methods (see Table 3 for complementary details for each method). Descriptions of and case studies for the key methods are provided in the proceeding sub-sections. For more detailed information, model description, and case studies refer to Supplementary Appendix Section SA1.

**TABLE 3:** Low-level: Clustering-based unsupervised integration methods.

Approach	Clustering method	Macro category*	Author	Objective	Omics data**	Software***
Kernel-based Clustering Methods	• L-MKKM (Localized Multiple Kernel *K*-Means)	ModE	Gönen and Margolin, (2014)	Sample-subtyping	CNV, DM, GE	• Matlab code (https://github.com/mehmetgonen/lmkkmeans)
	• SNF (Similarity Network Fusion)	ModE	Wang et al. (2014)	Disease-subtyping	Any Omics	• *MOVICS* (https://xlucpu.github.io/MOVICS/MOVICS-VIGNETTE.html)
						• *CEPICS* (https://rdrr.io/github/GaoLabXDU/CEPICS/)
						• *CancerSubtypes* (https://bioconductor.org/packages/release/bioc/html/CancerSubtypes.html)
	• rMKL-LPP (regularized Multiple Kernels Learning with Locality Preserving Projections)	ModE	Speicher and Pfeifer, (2015)	Disease-subtyping	DM, MiE, GE	• -
	• WSNF (Weighted SNF)	ModE	Xu et al. (2016)	Disease-subtyping	MiE, GE	• *CancerSubtypes* (https://bioconductor.org/packages/release/bioc/html/CancerSubtypes.html)
	• mixKernel	ModE	Mariette and Villa-Vialaneix, (2018)	Sample-subtyping	GE, MiE, DM	• *mixKernel* (https://cran.r-project.org/web/packages/mixKernel/index.html)
	• DSSF (Deep Subspace Similarity Fusion)	ModE	Yang et al. (2018)	Disease-subtyping	DM, MiE, GE	• -
	• ANF (Affinity Network Fusion)	ModE	Ma and Zhang, (2018)	Sample-subtyping	DM, MiE, GE	• *ANF* (https://bioconductor.org/packages/release/bioc/html/ANF.html)
	• NEMO (NEighborhood based Multi-Omics clustering)	ModE	Rappoport and Shamir, (2019)	Disease-subtyping	DM, MiE, GE	• *NEMO* (https://github.com/Shamir-Lab/NEMO)
						• *MOVICS* (https://xlucpu.github.io/MOVICS/MOVICS-VIGNETTE.html)
	• ab-SNF (association-signal-annotation boosted SNF)	ModE	Ruan et al. (2019)	Sample-subtyping	DM, GE	• R code (https://github.com/pfruan/abSNF/)
	• MvNE (Multiview Neighborhood Embedding)	ModE	Mitra et al. (2020)	Molecular-classification	DM, MiE, GE	• -
	• INF (Integrative Network Fusion)	DatE/ModE	Chierici et al. (2020)	Disease-subtyping, Disease-prediction	CNV, MiE, GE, PE	• Python/R code (https://gitlab.fbk.eu/MPBA/INF)
	• SmSPK (Smoothed Shortest Path graph Kernel)	ModE	Tepeli et al. (2020)	Sample-subtyping	GE, PE, Mutation	• Python code (https://github.com/tastanlab/pamogk)
	• PAMOGK (PAthway-based MultiOmic Graph Kernel clustering)	ModE	Tepeli et al. (2020)	Sample-subtyping	GE, PE, Mutation	• Python code (https://github.com/tastanlab/pamogk)
(Non-negative) Matrix Factorization-based Clustering Methods	• iCluster	ModE	Shen et al. (2009)	Disease-subtyping, Biomarker-identification	CNV, GE	• *iCluster* (https://cran.r-project.org/web/packages/iCluster/index.html)
						• *iClusterPlus* (https://bioconductor.org/packages/release/bioc/html/iClusterPlus.html)
						• *MOVICS* (https://xlucpu.github.io/MOVICS/MOVICS-VIGNETTE.html)
						• *CEPICS* (https://rdrr.io/github/GaoLabXDU/CEPICS/)
						• *CancerSubtypes* (https://bioconductor.org/packages/release/bioc/html/CancerSubtypes.html)
	• jNMF (Joint Non-negative Matrix Factorization)	ModE	Zhang et al. (2012)	Disease-insight, Module-discovery	MiE, DM, GE	• -
	• iClusterPlus	ModE	Mo et al. (2013)	Disease-subtyping	CNV, DM, GE	• *iClusterPlus* (https://bioconductor.org/packages/release/bioc/html/iClusterPlus.html)
				Biomarker-identification		
	• FA (Factor Analysis)	DatE	Liu et al. (2013)	Disease-subtyping	MiE, GE, PE	• *-*
	• moCluster	ModE	Meng et al. (2016)	Disease-subtyping, Molecular-subtyping	MiE, DM, PE	• *mogsa* (https://www.bioconductor.org/packages/release/bioc/html/mogsa.html)
						• *MOVICS* (https://xlucpu.github.io/MOVICS/MOVICS-VIGNETTE.html)
	• JIVE (Joint and Individual Variation Explained)	ModE	O’Connell and Lock, (2016)	Disease-subtyping	MiE, DM, GE	• *R.jive* (https://cran.r-project.org/web/packages/r.jive/index.html)
	• iNMF (integrative Non-negative Matrix Factorization)	ModE	Yang and Michailidis, (2016)	Disease-subtyping	MiE, DM, GE	• *MOVICS* (https://xlucpu.github.io/MOVICS/MOVICS-VIGNETTE.html)
						• Python code (https://github.com/yangzi4/iNMF)
	• PFA (Pattern Fusion Analysis)	ModE	Shi et al. (2017)	Disease-subtyping	MiE, DM, GE	• -
	• IS k -means (Integrative Sparse *k*-means)	DatE	Huo and Tseng, (2017)	Disease-subtyping	CNV, DM, GE	• IS-Kmeans (https://github.com/Caleb-Huo/IS-Kmeans)
	• MOGSA (Multi-Omics Gene-Set Analysis)	DatE	Meng et al. (2019)	Disease-insight	• GE, CNV, PE	• *Mogsa* (https://www.bioconductor.org/packages/release/bioc/html/mogsa.html)
	• SCFA (Subtyping via Consensus Factor Analysis)	ModE	Tran et al. (2020)	Disease-subtyping	• DM, MiE, GE	• R code (https://github.com/duct317/SCFA)
Bayesian Clustering Methods	• TMD (Transcriptional Modules Discovery)	ModE	Savage et al. (2010)	Disease-subtyping	GE, TF	• -
	• PARADIGM (PAthway Recognition Algorithm using Data Integration on Genomic Models)	ModE	Vaske et al. (2010)	Disease-subtyping and Disease-insight	CNV, GE, PE	• *GIANT* interface (http://giant.princeton.edu/)
	• PSDF (Patient-Specific Data Fusion)	ModE	Yuan et al. (2011)	Disease-subtyping	CNV, GE	• Matlab code (https://sites.google.com/site/patientspecificdatafusion/)
	• MDI (Multiple Dataset Integration)	ModE	Kirk et al. (2012)	Disease-subtyping	GE, PE	• Matlab code (https://warwick.ac.uk/fac/cross_fac/zeeman_institute/zeeman_research/software/)
	• BCC (Bayesian Consensus Clustering)	ModE	Lock and Dunson, (2013)	Disease-subtyping	MiE, DM, GE, PE	• *bayesCC* (https://github.com/ttriche/bayesCC)
	• LRAcluster (Low-Rank-Approximation)	ModE	Wu et al. (2015)	Disease-subtyping	CNV, DM, GE	• *LRAcluster* (http://lifeome.net/software/lracluster/)
						• *MOVICS* (https://xlucpu.github.io/MOVICS/MOVICS-VIGNETTE.html)
Multivariate and Other Clustering Methods	• COCA (Cluster-Of-Cluster Assignment)	ModE	Hoadley et al. (2014)	Disease-subtyping	MiE, CNV, DM, GE, PE	• *MOVICS* (https://xlucpu.github.io/MOVICS/MOVICS-VIGNETTE.html)
						• *coca* (https://github.com/acabassi/coca)
	• iPF (integrative Phenotyping Framework)	DatE	Kim et al. (2015)	Sample-subtyping	MiE, GE	• *iPF* (http://tsenglab.biostat.pitt.edu/software.htm)
	• Clusternomics	ModE	Gabasova et al. (2017)	Disease-subtyping	MiE, DM, GE, PE	• *Clusternomics* (https://github.com/evelinag/clusternomics)
	• PINS (Perturbation clustering for data INtegration and disease Subtyping)	ModE	Nguyen et al. (2017)	Disease-subtyping	MiE, CNV, DM, GE	• -
	• iDRW (integrative Directed Random Walk)	DatE	Kim et al. (2018)	Disease-subtyping, Biomarker-discovery	DM, GE	• R code (https://github.com/sykim122/iDRW)
	• PINSPlus	ModE	Nguyen et al. (2019)	Disease-subtyping	MiE, CNV, DM, GE	• *PINSPlus* (https://cran.r-project.org/web/packages/PINSPlus/index.html)
						• *MOVICS* (https://xlucpu.github.io/MOVICS/MOVICS-VIGNETTE.html)
	• Subtype-GAN	ModE	Yang et al. (2021)	Disease-subtyping	MiE, CNV, DM, GE	• R code (https://github.com/haiyang1986/Subtype-GAN)

*Macro categories include (A) Multi-step and Sequential Analysis (MS-SA), (B) Data-ensemble (DatE), (C) Model-ensemble (ModE). ** CNV: copy number variation, DM: DNA methylation, MiE: Micro RNA expression, GE: gene expression, TF: transcriptional factor, PE: Protein expression. ***R packages, unless otherwise stated.

#### Kernel-Based Clustering Method

The input data in the kernel-based methods is the kernel matrix ( $k\left(x_{i}^{\left(b\right)},x_{j}^{\left(b\right)}\right),\;b=1,\ldots ,B$ ), also called inter-patients similarities, instead of the original data ( $X=\left[X_{n\times p_{1}}^{\left(1\right)}|\ldots |X_{n\times p_{B}}^{\left(B\right)}\right]$ ). Therefore, the multi-Omics data integration problem is converted to kernel integration in the sample space ( $R^{n}$ ) rather than the multi-Omics (feature) space ( $R^{n\times \left(p_{1}+\ldots +p_{B}\right)}$ ). As a result, the optimization problems in the kernel-based methods are called dimension-free, i.e., it does not depend on the total number of the features ( $p_{1}+\ldots +p_{B}$ ).

**SNF** (Similarity Network Fusion) (Wang et al., 2014) is a popular method for multi-Omics data integration and subtype analysis. It first builds a sample-by-sample similarity matrix (or network, where nodes are samples and edges are similarities between samples) for each dataset separately and then fuses them to a global (weighted) sample similarity network. The second step (network-fusion) uses a nonlinear message-passing theory-based method (Pearl, 2014) to fuse the similarity matrices. SNF may lead to false fusion since it does not distinguish between different data types. Another drawback of SNF is that it uses Euclidean distance to calculate the similarity matrices between the samples that often is incapable of capturing the intrinsic similarities between data points. To address this issue, **DSSF** (Deep Subspace Similarity Fusion) (Yang et al., 2018) employs an auto-encoder to improve the discriminative similarity between samples. **AFN** (Affinity Network Fusion) (Ma and Zhang, 2018) is also an extension of SNF that enables the consideration of patients' pairwise distances. To handle the unmatched samples (different sample sizes in different Omics-types), **NEMO** (NEighborhood based Multi-Omics clustering) (Rappoport and Shamir, 2019) is introduced that enables the computation of global kernel matrix without performing any imputation on the missing observation. **INF** (Integrative Network Fusion) (Chierici et al., 2020) is another extension that utilizes SNF within a predictive framework including RF (Breiman, 2001) (Random Forest) and LSVM (Cortes and Vapnik, 1995) (Linear Support Vector Machine). See Supplementary Appendix Section SA1.1 for more details and case studies.

**Illustrative Case-studies: SNF** is applied to different multi-omics data integration studies. In the 1^st^ application (2018) (Chiu et al., 2018), it is applied on a cohort of triple-negative breast cancers (TNBC) patients from TCGA (including CNV, miRNA, and mRNA expressions) to identify the different sub-groups of cancer patients. Results revealed a new TNBC classification scheme with three different clusters of patients. One of the clusters, interestingly, was enriched in the “non-basal” subtype (by PAM50), whereas PAM50 obtained the most common “basal-like” subtype. This nan-basal cluster showed more aggressive clinical characteristics and distinctive oncogenic features (including 38% basal-like2 and 50% luminal androgen receptor subtypes).

#### (Non-negative) Matrix Factorization-based Clustering Method

Standard factorization methods commonly use singular value decomposition (SVD), such as PCA. However, for some data types, such as genotypes, the original matrices are non-negative. SVD-based factorizations contain negative entries, making it difficult to interpret their results in some applications. In contrast, NMF (Nonnegative Matrix Factorization) (Lee and Seung, 2001) restricts the entries in matrix factors to be non-negative.

**iCluster** (Shen et al., 2009) simultaneously considers the association between different data types and the covariance structure within each datatype. It employs the principles of two methods, including probabilistic PCA(Tipping and Bishop, 1999) and a (spectral) relaxed version of k -means (Zha et al., 2001). It first uses the Gaussian latent variable model to compute the posterior mean of the (latent variable) components ( T ). It then calculates the class membership by employing the standard k -means algorithm. The integrative model can be written as follows:

$$X^{\left(b\right)}=W^{\left(b\right)}\prime T+\varepsilon^{\left(b\right)},for\;b=1,\ldots ,B,T∼N\left(0,I\right),\varepsilon^{\left(b\right)}∼N\left(0,\sigma^{\left(b\right)}\right),$$

where $W^{\left(b\right)}$ is the $\left(c-1\right)\times p^{b}$ matrix of coefficient (also called loading matrix), c is the number of the clusters, and T is the (c−1)×n matrix of latent variable components that are shared between the data tables and explains the correlation between the different data types ( n is the sample size); $\varepsilon^{\left(b\right)}$ indicates the remaining (unexplained) variances for each data type. Therefore, the final data matrix will become as $X=\left(X^{\left(1\right)},\ldots ,X^{\left(B\right)}\right)∼N\left(0,WW\prime +\sigma \right)$ . Authors have applied a lasso-based penalty on W in the final likelihood function. Cluster memberships are then calculated by applying k -means clustering on the posterior mean of the latent variable components ( E(T|X) ). **iNMF** (integrative Non-negative Matrix Factorization) (Yang and Michailidis, 2016) is a multi-table extension of NMF to account for heterogeneity between the multiple datasets by providing heterogenous estimations/combinations ( $V_{b}T_{b}$ ) *via* minimizing the following loss function (using partitioned factorization structure):

$$argmin_{W,V_{b},T_{b}}\overset{B}{\underset{b=1}{\sum }}\|X-\left(W+V_{b}\right)T_{b}{\|}_{F}^{2}+\lambda \overset{B}{\underset{b=1}{\sum }}\|V_{b}T_{b}{\|}_{F}^{2},for\;b=1,\ldots ,B$$

$$s.t.W\geq 0,T_{b}\geq 0,V_{b}\geq 0,$$

where V is a homogeneity parameter and enables to account for different degrees of heterogeneity in the multiple datasets (since larger values of V result in smaller heterogeneous components $V_{b}T_{b}$ ). $\|.{\|}_{F}$ is the Frobenius norm (Lee and Seung, 1999). The authors also adopted the sparse version of the iNMF by applying $L_{1}-norm$ to $T_{b}$ . Whereas iCluster-based methods, NMF-based methods do not rely on any model assumptions and allow each sample to fall in more than one class or be excluded from the classification (see Supplementary Appendix Section SA1.2 for more NMF-based and iCluster-based methods and illustrative case-studies).

**Illustrative Case-studies: iCluster** is applied in different studies, mainly for cancer subtyping. It is recently applied to a cohort of ovarian carcinoma patients (including CNV, DNA methylation, and mRNA expression) to identify prognostic biomarkers (Zheng et al., 2019). The results revealed three distinct clusters of samples and identified *UBB* (ubiquitin B) and *IL18BP* (interleukin 18 binding protein) genes as the most prognostic biomarkers. The results suggested that lower expression of these two genes may result in higher methylation and lower CNV. Therefore, evaluating the expression of these two genes can help in early tumor diagnosis. In another study, iCluster is applied to multi-Omics data of adult soft tissue sarcomas (Lazar et al., 2017). iCluster showed that SS-subtype (synovial sarcoma) was the most distinct sarcoma with partial/complete loss of chromosome 3p (45% of cases), high expression of *FGFR3* and *miR-183*, and methylation of the *PDE4A* promoter. Another cluster identified by iCluster mainly included LMS (Leiomyosarcoma) cases with high expression of *MYLK*, *MYH11*, *ACTG2*, *miR-143*, *miR-145,* lower inferred activity of the apoptosis pathway, and higher hormone receptor (ER/PR) levels. It inferred *PI3K*/*AKT* pathway activity. The authors also concluded that copy number changes were the most informatics Omics in characterizing these sarcomas (except SS).

#### Bayesian Clustering Method

In the Bayesian framework of a clustering task, class memberships are calculated using a probability model (such as a Dirichlet Process Mixture (DPM) model (MacEachern and Müller, 2000)) subject to a *priori* assumption about what the true relationship between the data might be, which is expressed as a probability distribution. This probability is then updated as new observations become available (which is captured by a posterior distribution). This approach enables the use of prior information informing the clusters (sub-samples, sub-disease, or sub-features). The DPM model is one of the most widely used Bayesian nonparametric methods in the multi-Omics (multitype) clustering-based data integration. For a tutorial on DPM models, refer to (Li et al., 2019).

**LRAcluster** (Low-Rank-Approximation) (Wu et al., 2015) is a low-rank probabilistic method (similar to iClusterPlus) for molecular classification that takes both continuous and categorical data as input. LRAcluster first models each datatype using a probabilistic model and combine them as $L\left(\Theta \right)=\overset{B}{\underset{b=1}{\sum }}L\left(\Theta^{\left(b\right)},X^{\left(b\right)}\right)$ , for b=1,…,B , where $\Theta^{\left(b\right)}$ is the parameter matrix for datatype b . Θ is the overall parameter matrix and is assumed to be a low-rank matrix that leads to the following optimization problem:

$$argmin_{\Theta }L\left(\Theta \right)+\lambda |\Theta {|}^{*},$$

where λ is the tuning parameter and $|\Theta {|}^{*}$ indicates the nuclear norm of Θ . An iterative, fast LRA of the parameter matrix is then applied to solve this optimization problem. The final clustering task is applied to the low-dimension subspace (using k -means) to find feature subtypes (refer to (Subramanian et al., 2020) for more details and examples). **MDI** (Multiple Dataset Integration) (Kirk et al., 2012), **TMD** (Transcriptional Modules Discovery) (Savage et al., 2010), **PSDF** (Patient-Specific Data Fusion) (Yuan et al., 2011), and **BCC** (Bayesian Consensus Clustering) (Lock and Dunson, 2013) are four closely related integrative methods that all adopt a DPM. However, MDI and BCC have the same objective (clustering and subtyping), and all can integrate more than two data types (for more details, see Supplementary Appendix Section SA1.3).

**Illustrative Case-studies: LRAcluster** is applied in a study of hepatocellular carcinoma (HCC) as the major subtype of liver cancer (Wang et al., 2019) to characterize the molecular alternation of the metastatic HCCs. The results identified a list of individualized molecules (including *TNC*, *LAMA2*, *LAMC3*, *PDGFRA*, *CYP2E1*, *CYP3A4*, *CYP2C8*, *CYP1B1*, *CPS1*, *TAT,* and *HPD*) significantly expressed between the primary tumor compare and portal vein tumor thrombosis. Therefore, an individualized differential analysis for sequencing data was proposed to automate the process of finding these individualized genes.

#### Multivariate and Other Clustering Method

**COCA** (Cluster-Of-Cluster Assignment) (Hoadley et al., 2014) integrates the single-Omics clusters using hierarchical clustering based on pairwise concordance between different Omics platforms (including mRNA, miRNA, DNA methylation, and mutation). **PINS** (Perturbation clustering for data INtegration and disease Subtyping) (Nguyen et al., 2017) is a disease sub-typing method. It first partitions the samples into k ( k∈[2…K] ) clusters, then builds the patient connectivity matrices based on the pairwise connectivity for each possible cluster (see Supplementary Appendix Section SA1.4 for more information).

**Illustrative Case-studies: COCA** has recently been applied to a cohort of Ugandan cervical carcinoma patients (both $HIV^{+}$ and $HIV^{-}$ ) that is the first comprehensive profiling (genomic, transcriptomic, epigenomic) of sub-Saharan African patients (Gagliardi et al., 2020). They could identify human papillomavirus (HPV)-clade-specific (clade A7 and A9) patterns of multiple Omics features, including DNA methylation and gene expression. For instance, upregulated genes in clade A7-samples (such as PXDN) are also upregulated in cancers that progress through the epithelial-mesenchymal transition; and DNA methylation is closely regulated through cell differentiation. The clustering result showed the loss of *E2* expression in the A7-enriched cluster due to HPV integration in clade A7-samples. However, the A9-enriched cluster showed partial HPV integration supporting the higher expression of episomal HPV genes (due to E2 expression) in these patients. Therefore, the authors hypothesized that clade A9-infected samples might have a more active HPV infection. In another application on glioblastoma cancer patients (Yuan et al., 2020), COCA could identify two novel subtypes, including HX-1 and HX-2 categorized by three CpG regions (∼*DUSP1*, *PHOX2*, *HOXA7*) and 15 gene mutations, including *PCDH1*, *CYP27B1*, *LPIN3*, *GPR32*, *BCL6*, *OR4Q3*, *MAGI3*, *SKIV2L*, *PCSK5*, *AKAP12*, *UBE3B*, *MAP4*, *TP53BP1*, *F5*, *RHOBTB1*.

### Network-Based Integration Methods

Some of the fundamental tasks of biological research are to prioritize the features (or groups of features) that exhibit similar profiles and tend to be functionally related/co-regulated (such as gene modules) and to identify the functional relationships between different biological features (such as gene co-expression and signaling pathways). Network-based approaches do not rely solely on statistical models, but also leverage information about functional relationships and interactions available in biological knowledge databases, when integrating multi-Omics data. A network is a graphical representation (including nodes and edges) of the relationships between discrete entities. In computational network biology, nodes usually represent different features (such as SNPs, CpGs, genes, proteins, metabolites, and or phenotypes, e.g., diseases), and edges represent the relationship between pairs of nodes. When two nodes are sharing an edge, they are called neighbors, adjacent, or directly connected. The adjacency matrix of a network is then an i×j matrix with elements $W_{ij}$ where $W_{ij}=1$ if and only if the pair of nodes ( $w_{i},w_{j}$ ) are directly connected (neighbors). Degree matrix is a diagonal matrix where diagonal elements indicate the degrees of each node (i.e., number of neighbors). Biological network-based methods aim to describe the global topology of disease and biomarker/module discovery. We grouped *unsupervised multi-Omics network* methods into four distinct categories based on their statistical approaches, including 1) matrix factorization-based, 2) Bayesian, 3) network propagation-based, and 4) correlation-based and other networks (see Table 4 for complementary details for each method). Descriptions of and case studies for the key methods are provided in the proceeding sub-sections. For more detailed information, model description, and case studies, refer to Supplementary Appendix Section SA2.

**TABLE 4:** Low-level: Network-based unsupervised integration methods.

Approach	Model	Macro category*	Author	Omics data**	Objective	Software***
Matrix Factorization-based (MF-based) Networks	• CMF/CMF-W (Collective Matrix Factorization)	ModE	Liany et al. (2020)	Any Omics	Outcome/Interaction-prediction	• Python code (https://github.com/lianyh)
	• NBS (Network-Based Stratification)	ModE	Hofree et al. (2013)	MiE, CNV, DM, GE, PE	Patient-subtyping	• pyNBS Python code (https://github.com/idekerlab/pyNBS)
	• DFMF (Data Fusion by Matrix Factorization)	ModE	Žitnik and Zupan, (2014)	GE, GO-terms, MeSH-descriptor	Gene function-prediction	• *-*
	• FUSENET	ModE	Žitnik and Zupan, (2015)	GE, Mutation	Disease-insight (Gene-Disease association- prediction)	• Python code (https://github.com/mims-harvard/fusenet)
	• Medusa	ModE	Zitnik and Zupan, (2016)	Any Omics	Module-discovery, Gene-Disease association- prediction	• Python code (https://github.com/mims-harvard/medusa)
	• MAE (Multi-view factorization AutoEncoder)	ModE	Ma and Zhang, (2019)	MiE, DM, GE, PE, PPIs	Disease-prediction	PyTorch code (https://github.com/BeautyOfWeb/Multiview-AutoEncoder)
	• DisoFun (Differentiate isoform Functions with collaborative matrix factorization)	ModE	Wang et al. (2020)	GE, IE	Disease-function Prediction	MATLAB code (http://mlda.swu.edu.cn/codes.php?%20name=DisoFun)
	• IMCDriver	DatE	Zhang et al. (2021)	GE, Mutation, PPIs	Gene-discovery	Python code (https://github.com/NWPU-903PR/IMCDriver)
	• RAIMC (RBP-AS Target Prediction Based on Inductive Matrix Completion)	ModE	Qiu et al. (2021)	AS, RBPs	Protein-prediction	MATLAB code (https://github.com/yushanqiu/RAIMC)
Bayesian Networks (Pearl, 2014) (BNs)	• PARADIGM (PAthway Recognition Algorithm using Data Integration on Genomic Models)	ModE	Vaske et al. (2010)	CNV, GE, PE	Disease-subtyping, Disease-insight	• *GIANT* interface (http://giant.princeton.edu/)
	• CONEXIC	ModE	Akavia et al. (2010)	GE, CNV	Gene-discovery	• -
Network Propagation-based Networks (Random walk-, and Network Fusion-based Methods)	• GeneticInterPred	ModE	You et al. (2010)	GE, PE	Interaction-prediction	• -
	• RWRM (Random Walk with Restart on Multigraphs)	ModE	Li and Li, (2012)	GE, PPIs	Gene-prioritizing	• -
	• TieDIE (Tied Diffusion through Interacting Events)	ModE	Paull et al. (2013)	GE, TF, PPIs	Module/sub-network detection	• Python code (https://sysbiowiki.soe.ucsc.edu/tiedie)
	• SNF (Similarity Network Fusion)	ModE	Wang et al. (2014)	MiE, DM, GE	Patient-subtyping	• *SNFtool* (https://cran.r-project.org/web/packages/SNFtool/index.html)
	• HotNet2	ModE	Leiserson et al. (2015)	SNV, CNA, GE, PPIs	Sub-network detection	• HotNet software (http://compbio.cs.brown.edu/projects/hotnet/)
	• NetICS	ModE	Dimitrakopoulos et al. (2018)	MiE, CNV, GE	Biomarker-prediction	• Matlab code (https://github.com/cbg-ethz/netics)
	• RWR-M (Random Walk with Restart for Multiplex networks)	ModE	Valdeolivas et al. (2019)	GE, Co-expression, PPIs	Gene-prediction	• R code (https://github.com/alberto-valdeolivas/RWR-MH)
	• RWR-MH (RWR for Multiplex-Heterogeneous networks)	ModE	Valdeolivas et al. (2019)	GE, Co-expression, PPIs	Gene-prediction	• *RandomWalkRestartMH* (http://bioconductor.org/packages/release/bioc/html/RandomWalkRestartMH.html)
	• MSNE (Multiple Similarity Network Embedding)	ModE	Xu et al. (2020)	CNV, DM, GE	Disease-subtyping	• Python code (https://github.com/GaoLabXDU/MSNE)
	• RWRF (Random Walk with Restart for multi-dimensional data Fusion)	ModE	Wen et al. (2021)	MiE, DM, GE	Disease-subtyping	• R code (https://github.com/Sepstar/RWRF/)
Correlation-based and Other Networks	• WGCNA (Weighted Gene Co-expression Network Analysis)	DatE	Langfelder and Horvath, (2008)	GE (from multiple platforms/species)	Gene-prioritizing	• *WGCNA* (https://horvath.genetics.ucla.edu/html/CoexpressionNetwork/Rpackages/WGCNA/)
	• GGM (Gaussian Graphical Model)	ModE	Krumsiek et al. (2011)	SNP, GE, Met	Metabolite-pathway reactions	• -
	• GEM (GEnome scale Metabolic models)	ModE	Shoaie et al. (2013)	GE, Met	Metabolite-subnetwork	• -
	• DBN (Deep Belief Network)	ModE	Liang et al. (2014)	MiE, DM, GE	Disease-subtyping	• Python code (https://github.com/glgerard/MDBN)
	• Lemon-Tree	ModE	Bonnet et al. (2015)	CNV, GE	Biomarker-discovery	• JAVA command (https://github.com/erbon7/lemon-tree)
	• TransNet (Transkingdom Network)	ModE	Rodrigues et al. (2018)	Any Omics	Causal network	• TransNetDemo R code (https://github.com/richrr/TransNetDemo)

*Main categories include (A) Multi-step and Sequential Analysis (MS-SA), (B) Data-ensemble (DatE), (C) Model-ensemble (ModE). ** CNV: copy number variation, CAN: copy number alternation, SNV: single nucleotide variation, DM: DNA methylation, AS: alternative splicing, MiE: Micro RNA expression, GE: gene expression, TF: transcriptional factor, IE: isoform expression, PE: protein expression, RBPs: RNA-Binding Proteins, PPI: Protein-protein interactions, Met: Metabolite. ***R packages, unless otherwise stated.

#### Matrix Factorization-Based (MF-Based) Networks

**NBS** (Network-Based Stratification) (Hofree et al., 2013) is a sample-stratification method that uses both network propagation algorithm and matrix factorization to construct the final subtypes. Therefore, it can be categorized under either of these categories. NBS integrates genome-scale somatic mutations with a gene-interaction network. It first maps the mutations for each sample onto a gene-interaction network from STRING (https://string-db.org/), Pathway Commons (https://www.pathwaycommons.org/), and HumanNet (https://www.inetbio.org/humannet/download.php), and constructs the patient-by-gene matrix ( $M_{0}$ ). Then network propagation is used to smooth the sample-mutation-gene network as follows:

$$M_{t+1}=\alpha \;M_{t}\;A+\;\left(1-\alpha \right)M_{0},$$

where A is a normalized adjacency matric of the gene-interaction network, α is a tuning parameter controlling the mutation diffusion. The smoothing (propagation) function runs iteratively till convergence. The result of this step is a network-smoothed profile where the elements indicate the network proximity of each gene to the mutated genes for a specific sample. **FUSENET** (Žitnik and Zupan, 2015) and **DFMF** (Žitnik and Zupan, 2014) are flexible about input data and their distributions. The latter does not treat the entire input data as a single matrix and therefore, enables the identification of data-specific factors. **Medusa** (Zitnik and Zupan, 2016) is a module-discovery method that partly uses the same methodology as **DFMF** (Data Fusion by Matrix Factorization) (Žitnik and Zupan, 2014) to construct a fused network (see Supplementary Appendix Section SA2.1 for more information). **MAE** (Multi-view factorization AutoEncoder) (Ma and Zhang, 2019) is a combination of matrix factorization and an autoencoder that enables the simultaneous embedding of both features (Omics) and samples *via* more complex nonlinear transformations. It first constructs an interaction graph for each datatype. To do so, the interactions among the feature in each datatype are represented as a network ( $N\in R_{+}^{p\times p}$ ). For instance, for proteome datatype, network N will be protein-protein interaction networks (PPIs) that are publicly available (such as Reactome https://reactome.org/). Note that MAE can also be categorized as a supervised *(deep) neural networks* method. **RAIMC** (RBP-AS Target Prediction Based on Inductive Matrix Completion) (Qiu et al., 2021) is based on inductive matrix completion (IMC), where integrated RNA-binding proteins (RBP) similarities were calculated based on RBP-regulating similarity and integrated alternative splicing (AS) event similarities were computed based on AS module-similarity. Then Gaussian interaction profiles (GIP) for RBPs and AS events are computed and combined using the fast kernel learning (FKL). Before completing the association matrix with IMC, a top-kk nearest neighbor model is applied to denoise the integrated similarity matrix. See (Ou-Yang et al., 2022) for a comprehensive review of matrix factorization methods for biomedical link prediction, including, IMCDriver (Zhang et al., 2021) and DisoFun (Wang et al., 2020).

**Illustrative Case-studies: NBS** is applied for patient-subtype identification and discriminating the somatic mutation profiles in uterine, ovarian, and lung cancer studies obtained from TCGA. The survival result based on the identified subtypes showed that ovarian cancer patients with the most aggressive tumor had a mean survival of 32 months compared to others (∼80 months). The fibroblast growth factor (FGF) signaling pathway was enriched for this sub-network of patients with the worst survival in concordance with previous studies indicating the FGF signaling pathway as a driver of tumor progression resistant to anti-VEGF therapy (Cole et al., 2010). The next subtype of patients with relatively better (higher) survival was mainly enriched in DNA damage–response genes (including *ATM*, *ATR*, *BRCA1*, *BRCA2*, *RAD51*, and *CHEK2)* that have been referred to as *BRCAness* in previous studies (Konstantinopoulos et al., 2010).

#### Bayesian Networks (BNs)

Bayesian networks (BNs) are a combination of (directed acyclic) graph/network theory and probability models. Suppose N=(V, E) is a network where V is a vector of nodes and E is the set of edges. The structure of N in BNs is a directed acyclic graph (DAG) that defines the factorization of the joint probability of $V=\left\{X^{\left(1\right)},\ldots ,X^{\left(B\right)}\right\}$ into a set of local probability distributions (one for each $X^{\left(b\right)}$ ) *via* Markov property (Korb and Nicholson, 2010) of BNs:

$$P\left(X^{\left(1\right)},\ldots ,X^{\left(B\right)}\right)=\overset{B}{\underset{b=1}{\prod }}P\left(X^{\left(b\right)}|\pi_{X^{\left(b\right)}}\right),$$

showing that each node (random variable $X^{\left(b\right)}$ ) directly depends only on its parents $\pi_{X^{\left(b\right)}}$ (Scutari, 2009). The main disadvantage of BNs is their computational complexity since the number of network structures grows exponentially with the number of nodes. However, using the Monte Carlo Markov Chain (MCMC) approach can partially help the situation (Lin and Lane, 2017). **PARADIGM** (PAthway Recognition Algorithm using Data Integration on Genomic Models) (Vaske et al., 2010) can also be categorized as a BN approach. It uses the prior knowledge of the given pathways to model the nodes (Omics data). **CONEXIC** (Akavia et al., 2010) is another BN that aims to find cancer-driver mutations by integrating gene expression and CNVs.

**Illustrative Case-studies: CONEXIC** is applied to gene-CNV paired data from melanoma patients (Lin et al., 2008) to identify a list of cancer driver genes. First, a list of candidates was generated using CNV data, and then the most likely drivers were collected by integrating CNV and mRNA expression. It resulted in several modulators that explain the behavior of 7869 genes. Many of the top modulators were involved in melanoma-related pathways and included known oncogenes and tumor suppressors. CONEXIC could successfully pick known cancer-related genes out of a large region with many underlying genes. For instance, *CCNB2* (cell-cycle regulator) was selected from a large, amplified region. Finally, an automated literature-mining method called LitVAn (literature vector analysis) was used to find overrepresented terms in published studies. It resulted in a few well-known activated features in melanoma (such as *PI3K*, *MAPK*) and a novel process called “RAB” (Rabs regulate vesicular trafficking).

#### Network Propagation-Based (NP-Based) Networks

**Network Propagation (NP) (** Cowen et al., 2017) is a stochastic process that tracks each node’s flow and tries to amplify the signals through prior information and pass them to its neighborhoods over time. Suppose N=(V, E) is a network with an adjacency matrix W . Suppose $p_{0}\left(v\right)$ indicates the starting value of prior (known) information for node v∈V . For instance, it can be a vector of 0 and 1, 1 indicating the genes known to be related to the disease, and 0 otherwise. The value of $p_{0}\left(v\right)$ is the amount of information that we want to flow (diffuse) from each node to its neighborhoods. Therefore, the amount of information of node v (also called the state of node v ) at time t ( $p_{t}\left(v\right)$ ) can be formulated as the sum of the information of its neighbor ( N(v) ) at the previous time ( t−1 ):

$$p_{t}\left(v\right)=\sum_{u\in N\left(v\right)}p_{t-1}\left(u\right)\;w\left(u,v\right),$$ (1)

where w(u,v) indicates the (normalized) weights between nodes u and v and is based on the relationship/interaction between these two nodes. The result of this iterative propagation process (for t times) is the gene-ranks ( $p_{t}\left(v\right)$ ). Eq. 1 can be re-written with matrix notation as follows:

$$p_{t}=W^{*}p_{t-1},$$

where $W^{*}$ is a transition matrix and calculated from the adjacency matrix W . The random walk with restart—**RWR** (Tong et al., 2008) is a propagation algorithm that allows a walker (an imaginary particle) to start a walk (flow) from the initial node $v_{0}\in V$ (with prior probability $p_{0}$ ) to node $v_{t-1}$ at a discrete-time step t−1 ( $∼p_{t-1}$ ). It then walks from node $v_{t-1}$ to the next (randomly selected) neighbor $v_{t}$ by following a given transition matrix. Therefore, $p_{t}$ can be written as:

$$p_{t}=\alpha p_{0}+\left(1-\alpha \right)W_{t-1}^{*},$$

where α is called restart probability and controls the amount of prior information considered in the network, and $W^{*}$ is a normalized transition matrix. Different algorithms may use different transition matrices. **RWRM** (Random Walk with Restart on Multigraphs) (Li and Li, 2012) is one of the first extensions of network propagation for integrating multigraph gene networks. It enables multiple edges between two nodes [see Supplementary Appendix Section SA2.2 for extensions of RWR algorithm, including **RWR-M** (Valdeolivas et al., 2019) and **RWR-MH** (Valdeolivas et al., 2019)]. **TieDIE** (Tied Diffusion through Interacting Events) (Paull et al., 2013) accepts a biological graph/pathway (such as PPIs or gene interaction networks) and a set of prior scores for each node indicating the involvement of each node in the network. **SNF** (Wang et al., 2014) can be considered as both a clustering and network-based method. We have discussed SNF and its extensions in *Unsupervised Multi-omics Data Integration Methods*.

Based on a benchmarking study (Picart-Armada et al., 2019) for network propagation methods, selecting a prominent network analysis method is not clear-cut. The authors concluded that network propagation methods enable the biomarker discovery, but their efficiency greatly depends on the input biological network and the nodesʼ initial score (see Supplementary Appendix Section SA2.2 for more information).

**Illustrative Case-studies: SNF** is applied to identify GBM subtypes vis integrating DNA methylation, mRNA, and miRNA expressions (Wang et al., 2014). The results indicated that most edges in the similarity network (patientsʼ similarities) were only detectable when two or more types of Omics information has applied. SNF could successfully distinguish the previously reported IDH subtype (Sturm et al., 2012) consisting of younger patients with an *IDH1* mutation. SNF could further identify a subtype of patients who were more responsive to temozolomide, TMZ (a common GBM treatment), whereas another distinct subtype of patients with overexpressed CTSD and less responsive to TMZ [which is consistent with an *in vitro* study (Sun et al., 2012)]. SNF has recently been applied to TNBC (Chiu et al., 2018) and pancreatic cancers (Sinkala et al., 2020) to identify disease subtypes.

#### Correlation-Based and Other Networks

**WGCNA** (Weighted Gene Co-expression Network Analysis) (Langfelder and Horvath, 2008) is a gene-prioritizing correlation-network-based method that also enables gene module identification. Correlation networks are based on the correlation between a node and an outcome. The significance of a node (such as a gene) is then determined based on either the correlation coefficient or a regression-based *p*-value. WGCNA can be employed to find gene modules, sub-modules, and marker-prioritization. **Lemon-Tree** (Bonnet et al., 2015) is a biomarker-discovery method that first processes (normalize) the expression data (mRNA) and finds the co-expressed clusters of genes *via* a model-based Gibbs sampler (Joshi et al., 2008). It then employs ensemble methods (including spectral edge clustering algorithm) to identify gene modules and regulatory based on the co-expressed genes. Other Omics features (including miRNA, CNV, DNA methylation, and genotype) are added to the model as additional candidates and are combined to calculate the regulatory scores. Lemon-Tree also enables the gene ontology enrichment analysis for the modules. **DBN** (Deep Belief Network) (Liang et al., 2014) is a sample-classification (deep-learning, DL) method that integrates mRNA, miRNA, and DNA methylation data. DL methods are initially constructed from multi-layered (or deep) artificial neural networks (Bishop, 1995) (ANNs), inspired by actual NNs in the brain. ANN is a parallel system that accepts the input data in its first layer (input layer). It then passes the data into one or more hidden layers that ultimately connect them to an output layer. ANNs used for DL have more hidden layers where each of them helps to refine its previous layer by running a feature construction task. DBN applies a Gaussian restricted Boltzmann machines (Gaussian RBM) (Hinton, 2012) model to obtain the featuresʼ conditional distribution. An RBM consists of a visible layer (a layer of p visible Omics features) and a hidden layer (a layer of g hidden variable). The Gaussian RBM model assumes that the conditional distributions of visible variables (Omics features) given hidden variables follow a Gaussian distribution.

**Illustrative Case-studies: Lemon-Tree** has been applied to TCGA glioblastoma expression and copy-number data (Bonnet et al., 2015). It resulted in a module network composed of 121 clusters of co-expressed genes and a list of prioritized (high-scored) genes, mostly associated with amplified/deleted regions. Several of these high-scored genes were already reported as cancer genes in glioblastoma (including *EGFR*, *PDGFRA*, *FGFR3*, *PIK3CA*, *MDM4*, *CDKN2A/B*, and *PTEN*) where all involved in glioblastoma driver pathways, including proliferation, apoptosis, and angiogenesis pathways. Besides the well-known genes, Lemon-Tree could also identify a few novel markers that have rarely or never studied glioblastoma. For instance, *INSR* was involved in several modules. It stimulates cell proliferation and is aberrantly expressed in cancer cells (Belfiore et al., 2009); therefore, amplification of *INSR* in glioblastoma may enhance proliferation. *PAOX* (polyamine oxidase) is another novel marker that might have tumor suppressor activity via amine oxidase activity and their primary involvement in cancer growth inhibition and progression (Guzeloglu-Kayisli et al., 2004). Interestingly, *PAOX* was biologically relevant based on its prognostic value via a survival analysis.

## Conclusion

This paper reviews key methodologies to perform *unsupervised* multi-Omics data integration. We grouped the methods into three categories, including regression/association-based, clustering-based, and network-based methods. In each category, we then categorized the methods based on the statistical approach employed. Each of the methods has also been assigned to one of the following “macro” categories: (A) multi-step and sequential analysis (MS-SA), (B) data-ensemble (DatE), and (C) model-ensemble (ModE) (see Table 1 and Figure 2).

The majority of multi-Omics integration methods were applied to cancer data and mainly focused on genome and transcriptome integration. Therefore, the community needs to devote more efforts to make more publicly available data sources with more diverse Omics profiles (such as metabolome) and environmental/health factors. Many of the reviewed methods use custom pipelines where combinations of multiple methods are employed to answer the underlying biological question. Therefore, many of these methods are highly dependent on the input Omics data and prior information, making it difficult to compare these methods.

There are a few benchmarking studies for some of the methods we reviewed here (mostly clustering methods). For instance, comparison and benchmarking of unsupervised multi-Omics clustering algorithms (including, LRAcluster, MCCA, SNF, PINS, MCIA, moCluster, iClusterPlus) have been performed using both real data (multiple cancer from TCGA (Rappoport and Shamir, 2018) and a dataset of kidney renal clear cell carcinoma patients (Tepeli et al., 2020)), and simulated data (Pierre-Jean et al., 2020). Network propagation methods have been compared using multiple non-cancerous data (Picart-Armada et al., 2019). Graph- and kernel-based integration methods have been compared using cancer and hypertension data (Yan et al., 2017).

Future efforts should be directed toward 1) integrating more various types of data (including Omics, clinical, and environmental), 2) integrating into a universal pipeline, and 3) integrating the a priori biological knowledge into the system. For instance, in most cases, we have access to quantitative trait information, which can help to improve the feature’s weight assignment and prioritization and increase the accuracy of the prediction/classification tasks. One of the key challenges in integrating large-scale and heterogeneous Omics data is the small sample size and, therefore, most of the methods are data-hungry. One informative way around this issue is to leverage this extra a priori biological information into the method. Although it is beyond the scope of this review, many of the reviewed methods can, in principle, leverage this extra information. Unsupervised deep learning (DL) methods can be a good solution for considering the biological structure among the -Omics data, such as the hierarchical path from DNA to RNA and further to protein. Therefore, more effort should be devoted to utilizing DL for multi-Omics data integration problems with limited (small) sample sizes. Moreover, as noted in the Introduction, there might be multiple outcome variables (such as time-to-cure, or cancer-stage) which are mostly considered one-by-one in the available methods. Multivariate modeling (i.e., with multiple outcome variables) of multi-Omics profiles may provide a more realistic picture than looking at a single outcome, and therefore provides a more powerful test of significance. Lastly, most of the reviewed methods are applied on two or three different Omics modalities, however, in principle/theory, it is possible to extend these methods for more than 2 modalities, although the technical issues become more involved.
