# Supplementary material for: Unsupervised Multi-Omics Data Integration Methods: A Comprehensive Review
Source: Front Genet. 2022 Mar 22;13:854752. doi: 10.3389/fgene.2022.854752 (PMC8981526; doi:10.3389/fgene.2022.854752)
Supplement: Supplementary file 1 [file Table1.DOCX]

**APPENDICES**

*Unsupervised Multi-Omics Data Integration Methods: A Comprehensive Review*

# A1. More Details: Clustering-based Integration Methods

## A1.1 More details on: Kernel-based Clustering Method

**MKL** (Multiple kernel learning)(Gönen and Alpaydın, 2011) is a multiple-data learning method that first compute kernel (similarity) matrices for each data table separately, and then combine them - global kernel: $k\left( x_{i},x_{j} \right)=f\left( k_{1}\left( x_{i}^{(1)},x_{j}^{(1)} \right),\ldots,k_{B}\left( x_{i}^{(B)},x_{j}^{(B)} \right) \right)$ - to be used as the input of the final model. The simple form of the global kernel is a weighted linear combination of all kernel matrices:

$$k\left( x_{i},x_{j} \right)=\sum_{b=1}^{B} w_{b}k_{b}\left( x_{i}^{(b)},x_{j}^{(b)} \right), w_{b}\geq0.$$

where $w\in R^{p}$ is the vector of kernel weights. Localized multiple kernel k-means(Gönen and Margolin, 2014) (LMKKM) is an extension of MKL that also considers sample-specific weights beside the kernel weights. Therefore, the global kernel becomes $k\left( x_{i},x_{j} \right)=\sum_{b=1}^{B} w_{ib}w_{jb}k_{b}\left( x_{i}^{(b)},x_{j}^{(b)} \right)$, where $w\in R^{n\times p}$ is a matrix of sample-specific kernel weights. **rMKL-LPP** (regularized Multiple Kernels Learning with Locality Preserving Projections)(Speicher and Pfeifer, 2015) is a regularized extension of MKL for subtype identification that enables simultaneous integration and dimensionality reduction by employing the locality preserving projections (LPP)(Thrun *et al.*, 2004) method. LPP clusters each sample to its k-nearest neighbors; hence, samples are projected in a lower-dimensional space. None of the abovementioned methods considers the correlation between the kernels, and therefore, they may select mutually redundant kernel matrices in the computation of the global kernel. To address this issue, **MKKM-MR** (Multiple Kernel K-means with Matrix-induced Regularization)(Liu *et al.*, 2016) was introduced to decrease redundancy between kernel matrices by employing the matrix-induced regularization (MR). **mixKernel**(Mariette and Villa-Vialaneix, 2018) is also an unsupervised MKL method. It first defines a kernel (similarity matrix) between samples for each Omics type. These kernels are then integrated to build the final global kernel via (1) consensus multiple kernels and (2) a sparse kernel. This global kernel can then be served as an input for further exploratory analysis (such as PCA and k-means) to get more insight into the relationships between the samples. mixKernel is, in fact, the name of a package that allows the performance of the multiple-kernel method. As described in the main text, **SNF** (Similarity Network Fusion)(Wang *et al.*, 2014) is another popular method for multi-Omics data integration and subtype analysis. It first builds a sample-by-sample similarity matrix (or network, where nodes are samples and edges, are pairwise similarities between samples) for each dataset separately and then fuses them to a global (weighted) sample similarity network. The second step (network-fusion) uses a nonlinear message-passing theory-based method(Pearl, 2014) to fuse the similarity matrices. Similarity matrix for samples $i$ and $j$ is defined as

$$W_{ij}=exp\left( -\frac{\rho_{x_{i},x_{j}}^{2}}{\mu\varepsilon_{i,j}} \right),$$

where $\mu$ (recommended range: [0.3,0.8]) is a hyperparameter, and $\varepsilon_{i,j}$ is defined as:

$$\varepsilon_{i,j}=\frac{mean\left( \rho\left( x_{i},N_{i} \right) \right)+mean\left( \rho\left( x_{j},N_{j} \right) \right)+\rho_{x_{i},x_{j}}}{3}.$$

where $\rho\left( x_{i},N_{i} \right)$ indicates the Euclidean distance between sample $i$ ($x_{i}$) and its neighbors ($N_{j}$). The global fused kernel is then computed as a normalized weighted matrix. **AFN** (Affinity Network Fusion)(Ma and Zhang, 2018) is an extension of SNF that enables the consideration of patients' pairwise distances. It then constructs the so-called *patient affinity matrix* by applying the kNN (k-nearest neighborhood) graph kernel to the pairwise distances. The final affinity matrix to compute the class memberships is a weighted sum of pairwise affinity matrices. To handle the partial multiple datasets (different sample sizes in different data types), **NEMO** (NEighborhood based Multi-Omics clustering)(Rappoport and Shamir, 2019) is introduced that enables the computation of global kernel matrix without performing any imputation on the observation. It first computes the similarity matrix (kernel) for each data type (each Omics) using Euclidean distance and based on the radial basis function kernel(Buhmann, 2003):

$$W_{i,j}^{(b)}=\frac{1}{\sqrt{2\pi\sigma_{ijl}}}exp\left( -\frac{{\|x_{i}^{(b)}-x_{j}^{(b)}\|}^{2}}{2\sigma_{ijl}^{2}} \right),$$

where $\sigma_{ijl}^{2}$ is a normalizing factor to control the density of samples. Then the relative similarity matrix for each data type (Omics) is defined, which measures the sample similarities relative to their k-nearest neighbors. Next, it fuses the similarity matrices into a global (average) similarity matrix. The global similarity matrix is then used for the final clustering goal using a spectral clustering method(Ng *et al.*, 2001). **INF** (Integrative Network Fusion)(Chierici *et al.*, 2020) is another extension of SNF that utilizes SNF within a predictive framework including random forest(Breiman, 2001) and linear support vector machine(Cortes and Vapnik, 1995). It first integrates the multi-Omics features using SNF and ranks the integrated features using a so-called feature ranking scheme(Chierici *et al.*, 2020) based on their contribution to the SNF-fused network, i.e., their network mutual information. It then employs a so-called compact random forest model which only considers the top-ranked multi-Omics feature to run the final task of disease classification and prediction. **PAMOGK** (PAthway-based MultiOmic Graph Kernel clustering)(Tepeli *et al.*, 2020) is a graph-kernel-based sample-subtyping method that incorporates pathway information. It consists of three steps, (1) converting each pathway to a gene-gene-interaction graph where nodes are genes and edges are exist if there are interactions between the nodes, (2) computing the patient similarities (kernel matrices) for each pathway (and each Omics) using graph-kernel that takes two graphs as input, (3) integrating the kernel matrices using a standard kernel clustering method (such as MKKM-MR(Liu *et al.*, 2016)).

**Illustrative Case-studies:** **SNF**(Wang *et al.*, 2014) application on a Chinese cohort of TNBC patients (2019)(Jiang *et al.*, 2019) (including genomic, transcriptomic, and clinical data), revealed four distinct transcriptome-based subtypes, including luminal androgen receptor (LAR), immunomodulatory, basal-like immune-suppressed, and mesenchymal-like. LAR cluster showed more somatic mutation of *ERBB2*, infrequent signature 3, and *CDKN2A* loss. In another study on a cohort of patients with medulloblastoma(Cavalli *et al.*, 2017), SNF could identify/define the intertumoral heterogeneity within the common medulloblastoma subgroups. First, DNA methylation and mRNA expression were integrated using SNF, and the results identified homogeneous clusters of patients. It was followed by integrating the cluster-specific CNV and clinical factors (by SNF), which revealed further 12 distinct disease subtypes and could distinguish between groups 3 and 4 medulloblastoma. **L-MKKM**(Gönen and Margolin, 2014) is applied on colon and rectal cancer data set from TCGA. Three commonly used biomarkers of colon/rectal cancer, including microsatellite instability, hypermutation, and mutation in BRAF oncogene, were used to evaluate the accuracy of the LMKK-M results. Interestingly, the results showed that the hypermutated genomic markers (especially for CNV) clusters are much less noisy than those of multi-Omics data analysis, i.e., CNV was the most informative Omics platform and could very well characterize some of the cancer patients. **AFN**(Ma and Zhang, 2018) is applied to five different cancer types. We also find that it is usually not the case that integrating three datatypes would generate better results than that integrating two datatypes. One possible reason is that clustering using DNA methylation beta values performs much better than using FPKM and normalizes miRNA expression values for all four cancer types, suggesting that DNA methylation data may contain highly relevant information about dis-ease types.

## A1.2 More details on: (Non-negative) Matrix Factorization-based Clustering Method

**NMF**(Lee and Seung, 2001) is one of the popular methods for variable selection and clustering in a single data setting. There are several extensions of NMF to deal with multi-table data settings (${X=[X}_{n\times p_{1}}^{(1)}|\ldots|X_{n\times p_{B}}^{(B)}$]) and the heterogeneity among them. (Ding *et al.*, 2005), proved the equivalence between NMF and k-means(MacQueen, 1967). Therefore, we categorize both NMF-based and k-mean-based methods under the same category. **iClusterPlus**(Mo *et al.*, 2013) is a categorical extension of iCluster(Shen *et al.*, 2009) that allows the analysis of categorical data (binary, multinomial, and count) via employing the generalized regression models (GLMs). iClusterPlus also enables the identification of features (Omics) associated with each subtype (cluster) through a lasso-penalized likelihood approach. **moCluster**(Meng *et al.*, 2016) has a similar approach as iCluster where it decomposes each datatype into a lower-dimension product of latent components ($T$) and loading matrices ($W^{(b)}$). $T$s are then clustered using a standard clustering method (such as k-means). Whereas iCluster (combination of probabilistic PCA and k-means), moCluster is based on CPCA and assumes that all data types have the same variance (ignoring the heterogeneity among Omics datasets). Therefore, it performs faster than iCluster. **JIVE** (Joint and Individual Variation Explained)(Lock *et al.*, 2013) is an extension of iCluster that allows the data-specific latent component instead of a shared one:

$$X^{(b)}=W^{(b)}́T+W^{*(b)}́T^{*(b)}+\varepsilon^{(b)}, for b=1,\ldots,B,$$

where $T^{(b)}$ is the latent component for datatype $b$. Therefore, JIVE decomposes each datatype into three components, including low-rank approximations of shared component (explains the variation between datatypes) and data-specific component (explains the variation within a data type), and the error term (remaining unexplained variation in data). **jNMF** (Joint NMF)(Zhang *et al.*, 2012) is developed to integrate multiple datasets via minimizing the same loss function as NMF(Lee and Seung, 2001) for each dataset:

$${argmin}_{w,T_{b}}\sum_{b=1}^{B} {\|X-wT_{b}\|}_{F}^{2}, for b=1,\ldots,B$$

$subject to w\geq0, T_{b}\geq0$,

where ${\|.\|}_{F}$ indicates the Frobenius norm. jNMF has the same methodology as standard NMF and can be considered a multiple-NMF subject to a shared matrix of weights and a different matrix of components for each dataset. Therefore, the main limitation of jNMF is that it does not fully distinguish between different data tables and the heterogeneity among them (it considers homogenous estimations, $wT_{b}$). jNMF can also be considered as a semi-supervised method. **PFA** (Pattern Fusion Analysis)(Shi *et al.*, 2017) is another recently introduced subtyping (disease-subtyping) method that has methodological similarities with iCluster and SNF. It first applies a PCA for each data type to reduce the dimensionality and calls the resulting components in each data type as “local sample-spectrum”. It then fuses these lower space components to find the global component called “global sample-spectrum”. During the data fusion step, PFA calculates the contribution of each data type (or each sample) on the “global sample-spectrum” and corrects the effect of noises iteratively. **IS-K means** (Integrative Sparse K-means)(Huo and Tseng, 2017) is a multi-Omics extension of sparse k-means(Witten and Tibshirani, 2010) that employs a GL(Chen *et al.*, 2012) (overlapping group-lasso) – similar to iCluster – and a fast optimization technique using alternating direction method of multiplier(Boyd *et al.*, 2011) (ADMM) for feature selection and subtype identification. The objective function in the k-means method is to minimize the within-cluster sum of square (WCSS) as follows:

$${argmin}_{C}\sum_{j=1}^{p} \sum_{k=1}^{K} \frac{1}{n_{k}}\sum_{l,m\epsilon C_{k}} \left( X_{jl}-X_{jm} \right)^{2},$$

where $K$ (pre-specified) is the number of clusters, $n_{k}$ is the sample size in cluster $k$, $p$ is the total number of features ($p=p_{1}+\ldots+p_{B}$), and $C=\left( C_{1},\ldots,C_{K} \right)$ denotes the results of the clustering (partition of samples into $K$ clusters). On the other hand, minimizing the WCSS is equivalent to maximizing the between-cluster sum of squares (BCSS) since $WCSS+BCSS$ is a constant value (total sum of squares, TSS). In IS-K means method first normalizes the BCSS using TSS in each datatype ($R_{j}\left( C \right)=\frac{{BCSS}_{J}\left( C \right)}{{TSS}_{j}\left( C \right)}$), and then applies a lasso regularization into the objective function as follows:

$${argmin}_{C,w}-\sum_{j=1}^{p} w_{j}R_{j}\left( C \right)+\lambda\alpha{\|w\|}_{1}+\lambda\left( 1-\alpha\right)\Omega\left( w \right), s.t. {\|w\|}_{2}\leq1, w_{j}\geq0,$$

where $w_{j}$ denotes the weight for feature $j$, $w$ is a vector of weights for all features, $\Omega\left( w \right)$ denotes the overlapping group-lasso penalty, $\lambda$ is the penalty (tuning) parameter, and $\alpha\in\left[ 0,1 \right]$ controls the individual feature and group feature penalty. For instance, $\alpha=1$ means that there is no group feature selection that is equivalent to the sparse k-means. ADMM is then used to solve the optimization problem. The main advantages of IS-K means method are (1) utilizing the overlapping group-lasso enables the use of biological information, and (2) applying ADAM instead of EM reduces the complexity of this optimization problem compared to the standard k-means methods.

**Illustrative Case-studies:** **iClusterPlus**(Mo *et al.*, 2013) is applied to a Chinese cohort of prostate cancer (including whole genome, whole transcriptome, and DNA methylation)(Li *et al.*, 2020). The results revealed four distinct cancer subtypes and showed that the molecular alternation in the Chinese population was noticeably different from Western populations (mutations in *FOXA1* and deletions in *ZNF292* and *CHD1)*. They also showed the correlation between genome and epigenome alternations that can help in disease prediction and progression. iClusterPlus is applied to lung adenocarcinoma(Dong *et al.*, 2019) and cholangiocarcinoma(Jusakul *et al.*, 2017) data as well. **iNMF**(Yang and Michailidis, 2016) is applied on ovarian cancer data from TCGA to identify cancer subtypes by integrating mRNA expression, miRNA expression, and DNA methylation. The iNMF results revealed four disease subtypes (immunoreactive (I), proliferative (P), differentiated (D), and mesenchymal (M)), which were in great accordance with previous studies(Network, 2011). iNMF could successfully capture the heterogeneous noise between the Omics data and the molecular patterns within the ovarian cancer subtypes by comparing the resulting pathways in each module with published studies. For instance, genes involved in module I were mostly related to regulatory pathways controlling the cell cycle in ovarian cancer(Cunningham *et al.*, 2009). **moCluster**(Meng *et al.*, 2016) is applied on the NCI-60 cell line proteomics and mRNA data (from nine tissues) to identify the joint patterns and disease subtypes(Meng *et al.*, 2016). DAVID enrichment analysis using non-zeros genes and proteins showed the relationship between genes/proteins in the first latent component (super-feature) with DNA replication and DNA repair processes besides the lymphocyte/T cell activation that is in concordance with leukemia cell lines. Enrichment analysis using another latent component (#4) could further define a sub-group in the first cluster. This subgroup includes BT549 and MDAMB231 (claudin-low breast cancer cell lines), shown before that they have a higher expression of genes related to T cell, B cell, and granulocyte function(Sabatier *et al.*, 2014). Interestingly, three other cell lines, including SF268 (central nervous system), OVCR 8 (ovarian), and LOXIMVI (melanoma), were also found in this subgroup. It suggests the possibility of the presence of similar characteristics between these specific cancer cell lines. Results were tested for drug sensitivity via comparing the GI50 (50% cell growth inhibition) of a mitochondrial HSP90 ATPase inhibitor (Gamitrinib). The results showed different drug sensitivity in different cancer subtypes. moCluster is applied on colorectal cancer (from TCGA) (Meng *et al.*, 2016) and breast cancer (human mammary epithelial cells)(Fonti *et al.*, 2019) data as well. Besides the case studies mentioned in the main text, **iCluster** has recently been applied to multiplatform PanCancer Atlas study(Campbell *et al.*, 2018), oesophageal cancer(Network, 2017b), skin cutaneous melanoma (SKCM)(Chen *et al.*, 2020), cervical cancer(Network, 2017a) and pulmonary sarcomatoid carcinoma (PSC)(Yang *et al.*, 2020) data.

## A1.3 More details on: Bayesian Clustering Method

As mentioned in the main text, **MDI** (Multiple Dataset Integration)(Kirk *et al.*, 2012), **TMD** (Transcriptional Modules Discovery)(Savage *et al.*, 2010), **PSDF** (Patient-Specific Data Fusion)(Yuan *et al.*, 2011), and **BCC** (Bayesian Consensus Clustering)(Lock and Dunson, 2013) are four closely related integrative methods that all adopt a DPM model– therefore, they are unsupervised integrative methods - and use a Bayesian approach to estimate the parameters of the DPM models. Whereas TMD and PSDF, MDI, BCC, and iCluster have the same objective (clustering and subtyping), and all can integrate more than two data types. Although, the methodological principals of iCluster (uses joint latent variable model approach) and others are significantly different. MDI first fits a DPM model for each data type and then merge these models via the following conditional prior (at the component level):

$$p\left( c_{i}^{\left( 1 \right)},\ldots,c_{i}^{\left( B \right)}|\phi\right)\propto\prod_{b=1}^{B} \pi_{c_{i}^{\left( b \right)}}b\prod_{b=1}^{B-1} \prod_{l=b+1}^{B} \left( 1+\phi^{\left( lb \right)}I\left( c_{i}^{\left( b \right)}=c_{i}^{\left( l \right)} \right) \right),$$

where $I$ is the indicator function, $\phi^{\left( lb \right)}$ is a parameter that controls the pairwise association (dependency) between the clusters, $c_{i}^{(b)}\in\left\{ 1,\ldots,N \right\}$ is the component allocation variable for feature $i$ in datatype $b$, and $\pi_{c_{i}^{(b)}}b$ is the mixture proportion related to the component $c_{i}^{(b)}.$ Value of $N$ identifies the upper bond of the clusters’ number, which is suggested to set as $N=n/2$ by the authors (to be computationally affordable as well as achieve a reasonable clustering performance). The main improvements of MDI approach over its alternatives are (1) that the data types are not clustered independently, i.e., the allocation of a feature to clusters in one datatype has an effect on the allocation of this feature to clusters in another data type (called dependent component allocation); (2) it models the correlation between different data types (Omics layers) within the modeling framework; and (3) the optimal number of clusters are automatically estimated. Dependent component allocation is helpful when, for instance, there is a collection of mRNA expressions, for each of which some other information is measured from different Omics layers. **PSDF**(Yuan *et al.*, 2011) assigns a concordance status (1: concordant, 0: non-concordant) to each sample via MCMC (Markov chain Monte Carlo) sampling and then fuses only the concordant samples (therefore, it is called patient-specific fusion model). PSDF(Yuan *et al.*, 2011) is one of the first approaches distinguished between concordant and discordant data (among the samples). Among these methods, **BCC**(Lock and Dunson, 2013) relaxes the assumption of a common cluster (one global cluster structure for all datatypes) by allowing the different data types to follow different structures (called local clusters). MDI also considers the associations between Omics datatype when fusing the clusters. However, the limitation of MDI approach is that it only considers the pairwise associations between datatypes. Especially in the multi-Omics data analysis, it is unrealistic to suppose that all Omics type (such as mRNA expression, DNA methylation, CNV, etc.) follows the same pattern and cluster structure. **Clusternomics**(Gabasova *et al.*, 2017) is also a Bayesian probabilistic (a DPM) approach (called context-dependent integrative clustering) that allows different Omics data types to follow different cluster structures - each Omics data is considered as a context that can have a different influence on the outcome.

**Illustrative Case-studies: BCC**(Lock and Dunson, 2013) is applied to a cohort of breast cancer patients from TCGA to identify cancer subtypes. The result revealed three clusters (including basal, Luminal A, and ER/PR-positive), consistent with other published studies on breast cancer. **PSDF**(Yuan *et al.*, 2011) is applied to a cohort of breast cancer patients and identified four distinct clusters of patients using mRNA expression and CNA. It can also distinguish between patients who share concordant signals across the Omics types (fused) and those with contradiction (unfused). The fused patients have well-defined structures, but unfused cases also carry some information. For instance, unfused patients in cluster 3 (and partly clusters 1, 2, 4) all have similar CANs but very different gene expression values suggesting the lack of gene expression signal-to-noise for these patients. Omics features selected by PSDF (60% CAN and 40% mRNA) included some of the most well-known biomarkers (such as 8q contains MYC, 17q contains BRCA1, and 17p encodes TP53). Further network module and KEGG pathway enrichment analysis supported the capability of PSDF.

## A1.4 More details on: Multivariate & Other Clustering Method

**iPF** (integrative Phenotyping Framework)(Kim *et al.*, 2015) is a hierarchical integrative and model-free strategy for sample-subtyping that allows the use of both continuous and categorical matrices of features. All data types are first standardized and concatenated to make a combined data matrix ($X$). Then multi-dimensional scaling is applied to convert the combined data matrix into a two-dimensional space (dimension reduction) and find feature coordinates. Then a non-parametric smoothing method (such as spline) and generalized additive model (GAM) are used to create a feature topology plot (FTP). **PARADIGM** (PAthway Recognition Algorithm using Data Integration on Genomic Models)(Vaske *et al.*, 2010) incorporates prior biological knowledge into a probabilistic modeling framework and provides a matrix of integrated pathway activities. **COCA** (Cluster-Of-Cluster Assignment)(Hoadley *et al.*, 2014) integrates the single-Omics clusters using hierarchical clustering based on pairwise concordance between different Omics platforms (including mRNA, miRNA, DNA methylation, and mutation). It is shown that both PARADIGM and COCA produce highly concordant classification results(Hoadley *et al.*, 2014). **iDRW** (integrative Directed Random Walk)(Kim *et al.*, 2018) enables the integration of information that exists in a gene-gene graph constructed from multi-Omics profiles (expression and methylation profiles). **PINS** (Perturbation clustering for data INtegration and disease Subtyping)(Nguyen *et al.*, 2017) is a disease sub-typing method. It first partitions the samples into $k$ ($k\in\left[ 2\ldots K \right]$) clusters, then builds the patient connectivity matrices ($C_{k}$) based on the pairwise connectivity for each possible cluster. The connectivity matrix elements are 1 (if and only if two samples belong to the same cluster) and 0 (otherwise). There are $K-1$ connectivity matrices, one for each choice of $k$. It then perturbs the original data and re-runs the previous steps to construct the connectivity matrices ($A^{k}$). Then the discrepancy between $C_{k}$ and $A^{k}$ is calculated ($D_{k}=|C_{k}-A_{k}|$) to assess the stability of clustering through the distribution of the elements of $D_{k}$ ($F_{k}$) – more shifts toward 1 indicates the less stable clustering. For instance, if $C_{k}$ and $A_{k}$ are identical, then $D_{k}$ will be a matrix of zeros and $F_{k}$ will be exactly 1 that indicates the most robust clustering (i.e., data perturbation did not change the clustering result). See also PINSPlus(Nguyen *et al.*, 2019), a tumor-subtyping tool using multi-Omics data. A drawback of PINS and COCA is that they do not consider the associations between different data types.

**Illustrative Case-studies:** Application of **COCA** in a study of urologic cancers(Chen *et al.*, 2017) identified nine distinct subtypes by integrating DNA methylation, CNV, mRNA and protein expressions. Further gene expression analysis resulted in a list of cancer subtype-specific genes that can help to span cancer types. Differences in patients’ survival and enrichment of specific pathways (such as hypoxia, metabolism, NRF2-ARE, Hippo, and immune checkpoint) could further distinguish the subtypes. COCA has also been applied in pan-cancer (Pan-Cancer-12)(Hoadley *et al.*, 2014), breast cancer(Aure *et al.*, 2017), tumor classification(Mamatjan *et al.*, 2017), and Immune infiltration of the 11 tumor types(Iglesia *et al.*, 2016).

# A2. More Details: Network-based Integration Methods

## A2.1 More details on: Matrix Factorization-based (MF-based) Networks

**FUSENET**(Žitnik and Zupan, 2015) is a Markov-network-based approach to simultaneously constructing networks from many different gene profile data that can have different distributions (such as mRNA expression from RNA-seq with Poisson or negative binomial distributions, whereas from microarray with Gaussian distribution). Markov network enables a more robust measurement to find the edges in a gene network. The main property of these methods is their ability to fuse multiple data types from nonidentical distributions. The same author introduced **DFMF** (Data Fusion with Matrix Factorization)(Žitnik and Zupan, 2014), which is also flexible about input data and takes any information that can be represented as a matrix. It first constructs the “relation” matrices ($R_{ij}\in R^{p_{i}\times p_{j}}$) that are the pairwise relations between all pairs of data types ($i,j\in\left\{ 1,2,\ldots.,B \right\}$). It then simultaneously constrains each relation matrix by a “constraint matrix” ($\Theta_{i}\in R^{p_{i}\times p_{i}}$) and fuses them all via a three-factor penalized factorization. Constraint matrix, $\Theta_{i}$, is a known data source providing the relations between features in $i^{th}$ datatype (such as PPIs for the proteome datatype). The main difference of this method is that it does not treat the entire input data as a single matrix. Therefore, it enables the identification of data-specific factors. **Medusa**(Zitnik and Zupan, 2016) is a module discovery method that takes different direct and indirect information between features (called data semantics) besides the heterogenous Omics data. For instance, the input can be a matrix of mRNA expression, pathways, disease symptoms, and disease ontology. It partly uses the same methodology as DFMF to construct a fused network where the nodes are different types of objects (such as DO and mRNA expression, and pathways), and edges represent the relations between each pair of nodes ($R_{ij}$). Medusa accepts as an input a set of predefined so-called “pivot” objects and a set of candidate features. It then aims to find a module (of size $k$) of the candidate features with respect to the pivots and to predict gene-disease association via collective matrix factorization(Žitnik and Zupan, 2014).

**Illustrative Case-studies: MAE**(Ma and Zhang, 2019) is applied on three TCGA datasets, including BLCA (urothelial bladder carcinoma, LGG (brain lower grade glioma), and pan-cancer to predict the progression-free interval (PFI) event (a binary clinical outcome endpoint(Liu *et al.*, 2018)). PPI network from STRING (<https://string-db.org/>) was used as the molecular interaction network, and miRNA-protein mapping was derived from miRDB (<http://www.mirdb.org/>) miRNA target prediction scores. These experiments are mainly utilized to discover the prediction power of MAE compared to other well-known methods (such as random forest and SVM). They also ran the experiments for single-Omics data. The results, collectively, showed the superiority of the MAE multi-Omics integrative method for disease/outcome prediction using known biological interaction networks and patient similarity networks.

## A2.2 More details on: Network Propagation-based Networks

**Network Propagation (NP)**(Cowen *et al.*, 2017) is a stochastic process that tracks each node's flow and tries to amplify the signals through prior information and pass them to its neighborhoods over time. To this end, prior biological information (such as gene-disease ontology) is overlayed on all nodes (such as genes). All this information is propagated via the edges with nearby nodes in an iterative manner for a fixed number of steps (local neighborhoods) or till convergence. Therefore, the final value of each node is influenced by its neighbors. NP is a powerful approach in, specifically, gene-prioritizing and ranking. For instance, suppose we have given a list of genes and are supposed to find a list of essential genes related to a specific phenotype/disease. Also, suppose that based on the previous studies and experiments (such as human or animal *de novo* studies at a genome level, i.e., GWAS results), we have access to a list of already known genes associated with the underlying phenotype/disease. NP allows us to overlay these known gene-disease interactions on the genes in our list (as the nodes of a network) and simultaneously update the network's gene-disease signal by updating each node (gene) with its all-possible neighbors. **RWRM** (Random Walk with Restart on Multigraphs)(Li and Li, 2012) is one of the first extensions of network propagation for integrating multigraph gene networks. It enables multiple edges between two nodes. Suppose $N_{i}$ is the number of networks that node $v$ belongs to, the transition probability of moving between nodes $v_{i}$ and $v_{j}$ is the weighted sum of the ${(i,j)}^{th}$ element of $W$ over $N_{i}$, where weights are the probability of selecting the corresponding network. **TieDIE** (Tied Diffusion through Interacting Events)(Paull *et al.*, 2013) accepts a biological graph/pathway (such as PPIs or gene interaction network) and a set of prior scores for each node indicating the involvement of each node (gene) in the network. Then each node will be assigned a new score by diffusing the prior scores onto the network as follows:

$$s=f\left( r\left( x,W \right), r\left( y,Ẃ \right) \right),$$

where $r\left( x,W \right)$ is called the relevance function that is a function of prior scores ($x$) and adjacency matrix ($W$), $f\left( . \right)$ is a function that assigns higher scores to nodes where both $r\left( x,W \right)$ and $r\left( y,Ẃ \right)$ are high and lower scores if both are low. **SNF**(Wang *et al.*, 2014) can be considered as both a clustering and network-based method. In summary, it converts each datatype (such as mRNA expression and DNA methylation) to a matrix of patient-by-patient similarity network, where nodes are patients and edges represent the similarities between a pair of patients. It then updates each network with information from the other network via iterative network fusion. The result of SNF will be a single (fused) patient-by-patient network. **NetICS**(Dimitrakopoulos *et al.*, 2018) is a per-sample network-based (cancer) gene prioritization method that enables the integration of multi-Omics data and biological networks. It can accept both continuous and categorical input (multi-Omics) data. It applies the insulated heat diffusion algorithm(Leiserson *et al.*, 2015), where the diffusion matrix is defined as:

$$\alpha\left[ I-\left( 1-\alpha\right)W^{*} \right]^{-1},$$

where $W^{*}$ is the normalized adjacency matrix, $\alpha$ is restart probability that controls the amount of diffusion in the network. NetICS calculates a ranked list of genes for each sample (sample-specific gene prioritization) besides the overall list of ranked genes across all samples. **RWR-M** (RWR for Multiplex networks)(Valdeolivas *et al.*, 2019) and **RWR-MH** (RWR for Multiplex-Heterogenous networks) (Valdeolivas *et al.*, 2019) is a recent extension of the RWR algorithm based on the novel theoretical framework called multiplex network(Castellani *et al.*, 2014) (multi-partite networks). Multiplex networks have multi-layer topological structures, but they are instead multi-partite networks, i.e., all layers (networks) consist of the same set of nodes. However, the edges between a pair of nodes represent different information in different layers. Suppose there are $L$ graphs (layers) that all share the same set of nodes and $W^{l}$ is the adjacency matrix of layer $l$ ($l=1,\ldots,L$) where its elements are 1 if a pair of nodes ($v_{i}^{l},v_{j}^{l}$) are connected in layer $l$, and 0 otherwise. Therefore, a multiplex graph is defined as $m=\left( V^{M},E^{M} \right)$, where $V^{M}$ is the vector of nodes in $L$ layers, and $E^{M}$ is the set of edges between each pair of nodes in each layer, with adjacency matrix ${W=W}^{1},\ldots,W^{L}$. In an RWR-M or RWR-MH model, a walker can walk through any neighbor of the initial node (within a layer) and can also jump to any node in another layer. The adjacency matrix on the RWR-M model is defined as follows:

$$W=\left[ \left( 1-\delta\right)W^{1} \frac{\delta}{\left( L-1 \right)}I \cdots\frac{\delta}{\left( L-1 \right)}I \left( 1-\delta\right)W^{2} \ldots\vdots\vdots\ddots\frac{\delta}{\left( L-1 \right)}I \frac{\delta}{\left( L-1 \right)}I \ldots\frac{\delta}{\left( L-1 \right)}I \frac{\delta}{\left( L-1 \right)}I \vdots\left( 1-\delta\right)W^{L} \right],$$

where $I\in R^{n\times n}$ is the identity matrix, and parameter $\delta\in\left[ 0,1 \right]$ controls the probability of jumping between the layers. For instance, $\delta=1$ indicates that a walker will always jump between the layers. The probability distribution is then defined as:

$$\underline{p}_{t}=\alpha\underline{p}_{RS}+\left( 1-\alpha\right)W^{*}\underline{p}_{t-1},$$

where $\underline{p}_{t}=\left[ p_{t}^{1},\ldots,p_{t}^{L} \right]$ is a vector of probability distributions in all layers, $\underline{p}_{RS}$ is the restart vector of initial probabilities.

# References

Aure MR, Vitelli V, Jernström S, Kumar S, Krohn M, Due EU*, et al.* (2017). Integrative clustering reveals a novel split in the luminal A subtype of breast cancer with impact on outcome. *Breast Cancer Research, 19*(1), 44.

Boyd S, Parikh N, Chu E. (2011). *Distributed optimization and statistical learning via the alternating direction method of multipliers*: Now Publishers Inc.

Breiman L. (2001). Random forests. *Machine learning, 45*(1), 5-32.

Buhmann MD. (2003). *Radial basis functions: theory and implementations* (Vol. 12): Cambridge university press.

Campbell JD, Yau C, Bowlby R, Liu Y, Brennan K, Fan H*, et al.* (2018). Genomic, pathway network, and immunologic features distinguishing squamous carcinomas. *Cell reports, 23*(1), 194-212. e196.

Castellani G, Intrator N, Remondini D. (2014). Systems biology and brain activity in neuronal pathways by smart device and advanced signal processing. *Frontiers in genetics, 5*, 253.

Cavalli FM, Remke M, Rampasek L, Peacock J, Shih DJ, Luu B*, et al.* (2017). Intertumoral heterogeneity within medulloblastoma subgroups. *Cancer cell, 31*(6), 737-754. e736.

Chen F, Zhang Y, Bossé D, Lalani A-KA, Hakimi AA, Hsieh JJ*, et al.* (2017). Pan-urologic cancer genomic subtypes that transcend tissue of origin. *Nature communications, 8*(1), 1-15.

Chen W, Cheng P, Jiang J, Ren Y, Wu D, Xue D. (2020). Epigenomic and genomic analysis of transcriptome modulation in skin cutaneous melanoma. *Aging (Albany NY), 12*(13), 12703.

Chen X, Lin Q, Kim S, Carbonell JG, Xing EP. (2012). Smoothing proximal gradient method for general structured sparse regression. *The Annals of Applied Statistics, 6*(2), 719-752.

Chierici M, Bussola N, Marcolini A, Francescatto M, Zandonà A, Trastulla L*, et al.* (2020). Integrative Network Fusion: a multi-omics approach in molecular profiling. *bioRxiv*.

Cortes C, Vapnik V. (1995). Support-vector networks. *Machine learning, 20*(3), 273-297.

Cowen L, Ideker T, Raphael BJ, Sharan R. (2017). Network propagation: a universal amplifier of genetic associations. *Nature Reviews Genetics, 18*(9), 551.

Cunningham JM, Vierkant R, Sellers T, Phelan C, Rider D, Liebow M*, et al.* (2009). Cell cycle genes and ovarian cancer susceptibility: a tagSNP analysis. *British journal of cancer, 101*(8), 1461-1468.

Dimitrakopoulos C, Hindupur SK, Häfliger L, Behr J, Montazeri H, Hall MN*, et al.* (2018). Network-based integration of multi-omics data for prioritizing cancer genes. *Bioinformatics, 34*(14), 2441-2448.

Ding C, He X, Simon HD. (2005). *On the equivalence of nonnegative matrix factorization and spectral clustering.* Paper presented at the Proceedings of the 2005 SIAM international conference on data mining.

Dong X, Zhang R, He J, Lai L, Alolga RN, Shen S*, et al.* (2019). Trans-omics biomarker model improves prognostic prediction accuracy for early-stage lung adenocarcinoma. *Aging (Albany NY), 11*(16), 6312.

Fonti C, Saumet A, Abi‐Khalil A, Orsetti B, Cleroux E, Bender A*, et al.* (2019). Distinct oncogenes drive different genome and epigenome alterations in human mammary epithelial cells. *International journal of cancer, 145*(5), 1299-1311.

Gabasova E, Reid J, Wernisch L. (2017). Clusternomics: Integrative context-dependent clustering for heterogeneous datasets. *PLoS computational biology, 13*(10), e1005781.

Gönen M, Alpaydın E. (2011). Multiple kernel learning algorithms. *The Journal of Machine Learning Research, 12*, 2211-2268.

Gönen M, Margolin AA. (2014). *Localized data fusion for kernel k-means clustering with application to cancer biology.* Paper presented at the Advances in neural information processing systems.

Hoadley KA, Yau C, Wolf DM, Cherniack AD, Tamborero D, Ng S*, et al.* (2014). Multiplatform analysis of 12 cancer types reveals molecular classification within and across tissues of origin. *Cell, 158*(4), 929-944.

Huo Z, Tseng G. (2017). Integrative sparse K-means with overlapping group lasso in genomic applications for disease subtype discovery. *The annals of applied statistics, 11*(2), 1011.

Iglesia MD, Parker JS, Hoadley KA, Serody JS, Perou CM, Vincent BG. (2016). Genomic analysis of immune cell infiltrates across 11 tumor types. *JNCI: Journal of the National Cancer Institute, 108*(11).

Jiang Y-Z, Ma D, Suo C, Shi J, Xue M, Hu X*, et al.* (2019). Genomic and transcriptomic landscape of triple-negative breast cancers: subtypes and treatment strategies. *Cancer cell, 35*(3), 428-440. e425.

Jusakul A, Cutcutache I, Yong CH, Lim JQ, Huang MN, Padmanabhan N*, et al.* (2017). Whole-genome and epigenomic landscapes of etiologically distinct subtypes of cholangiocarcinoma. *Cancer discovery, 7*(10), 1116-1135.

Kim S, Herazo-Maya JD, Kang DD, Juan-Guardela BM, Tedrow J, Martinez FJ*, et al.* (2015). Integrative phenotyping framework (iPF): integrative clustering of multiple omics data identifies novel lung disease subphenotypes. *BMC genomics, 16*(1), 924.

Kim SY, Kim TR, Jeong H-H, Sohn K-A. (2018). Integrative pathway-based survival prediction utilizing the interaction between gene expression and DNA methylation in breast cancer. *BMC medical genomics, 11*(3), 68.

Kirk P, Griffin JE, Savage RS, Ghahramani Z, Wild DL. (2012). Bayesian correlated clustering to integrate multiple datasets. *Bioinformatics, 28*(24), 3290-3297.

Lee DD, Seung HS. (2001). *Algorithms for non-negative matrix factorization.* Paper presented at the Advances in neural information processing systems.

Leiserson MD, Vandin F, Wu H-T, Dobson JR, Eldridge JV, Thomas JL*, et al.* (2015). Pan-cancer network analysis identifies combinations of rare somatic mutations across pathways and protein complexes. *Nature genetics, 47*(2), 106-114.

Li J, Xu C, Lee HJ, Ren S, Zi X, Zhang Z*, et al.* (2020). A genomic and epigenomic atlas of prostate cancer in Asian populations. *Nature, 580*(7801), 93-99.

Li Y, Li J. (2012). *Disease gene identification by random walk on multigraphs merging heterogeneous genomic and phenotype data.* Paper presented at the BMC genomics.

Liu J, Lichtenberg T, Hoadley KA, Poisson LM, Lazar AJ, Cherniack AD*, et al.* (2018). An integrated TCGA pan-cancer clinical data resource to drive high-quality survival outcome analytics. *Cell, 173*(2), 400-416. e411.

Liu X, Dou Y, Yin J, Wang L, Zhu E. (2016). *Multiple kernel k-means clustering with matrix-induced regularization.* Paper presented at the Proceedings of the thirtieth AAAI conference on artificial intelligence.

Lock EF, Dunson DB. (2013). Bayesian consensus clustering. *Bioinformatics, 29*(20), 2610-2616.

Lock EF, Hoadley KA, Marron JS, Nobel AB. (2013). Joint and individual variation explained (JIVE) for integrated analysis of multiple data types. *The annals of applied statistics, 7*(1), 523.

Ma T, Zhang A. (2018). Affinity network fusion and semi-supervised learning for cancer patient clustering. *Methods, 145*, 16-24.

Ma T, Zhang A. (2019). Integrate multi-omics data with biological interaction networks using Multi-view Factorization AutoEncoder (MAE). *BMC genomics, 20*(11), 1-11.

MacQueen J. (1967). *Some methods for classification and analysis of multivariate observations.* Paper presented at the Proceedings of the fifth Berkeley symposium on mathematical statistics and probability.

Mamatjan Y, Agnihotri S, Goldenberg A, Tonge P, Mansouri S, Zadeh G*, et al.* (2017). Molecular signatures for tumor classification: an analysis of the cancer genome atlas data. *The Journal of Molecular Diagnostics, 19*(6), 881-891.

Mariette J, Villa-Vialaneix N. (2018). Unsupervised multiple kernel learning for heterogeneous data integration. *Bioinformatics, 34*(6), 1009-1015.

Meng C, Helm D, Frejno M, Kuster B. (2016). moCluster: identifying joint patterns across multiple omics data sets. *Journal of Proteome Research, 15*(3), 755-765.

Mo Q, Wang S, Seshan VE, Olshen AB, Schultz N, Sander C*, et al.* (2013). Pattern discovery and cancer gene identification in integrated cancer genomic data. *Proceedings of the National Academy of Sciences, 110*(11), 4245-4250.

Network CGAR. (2011). Integrated genomic analyses of ovarian carcinoma. *Nature, 474*(7353), 609.

Network CGAR. (2017a). Integrated genomic and molecular characterization of cervical cancer. *Nature, 543*(7645), 378-384.

Network CGAR. (2017b). Integrated genomic characterization of oesophageal carcinoma. *Nature, 541*(7636), 169-175.

Ng A, Jordan M, Weiss Y. (2001). On spectral clustering: Analysis and an algorithm. *Advances in neural information processing systems, 14*, 849-856.

Nguyen H, Shrestha S, Draghici S, Nguyen T. (2019). PINSPlus: a tool for tumor subtype discovery in integrated genomic data. *Bioinformatics, 35*(16), 2843-2846.

Nguyen T, Tagett R, Diaz D, Draghici S. (2017). A novel approach for data integration and disease subtyping. *Genome research, 27*(12), 2025-2039.

Paull EO, Carlin DE, Niepel M, Sorger PK, Haussler D, Stuart JM. (2013). Discovering causal pathways linking genomic events to transcriptional states using Tied Diffusion Through Interacting Events (TieDIE). *Bioinformatics, 29*(21), 2757-2764.

Pearl J. (2014). *Probabilistic reasoning in intelligent systems: networks of plausible inference*: Elsevier.

Rappoport N, Shamir R. (2019). NEMO: Cancer subtyping by integration of partial multi-omic data. *Bioinformatics, 35*(18), 3348-3356.

Sabatier R, Finetti P, Guille A, Adelaide J, Chaffanet M, Viens P*, et al.* (2014). Claudin-low breast cancers: clinical, pathological, molecular and prognostic characterization. *Molecular cancer, 13*(1), 228.

Savage RS, Ghahramani Z, Griffin JE, De la Cruz BJ, Wild DL. (2010). Discovering transcriptional modules by Bayesian data integration. *Bioinformatics, 26*(12), i158-i167.

Shen R, Olshen AB, Ladanyi M. (2009). Integrative clustering of multiple genomic data types using a joint latent variable model with application to breast and lung cancer subtype analysis. *Bioinformatics, 25*(22), 2906-2912.

Shi Q, Zhang C, Peng M, Yu X, Zeng T, Liu J*, et al.* (2017). Pattern fusion analysis by adaptive alignment of multiple heterogeneous omics data. *Bioinformatics, 33*(17), 2706-2714.

Speicher NK, Pfeifer N. (2015). Integrating different data types by regularized unsupervised multiple kernel learning with application to cancer subtype discovery. *Bioinformatics, 31*(12), i268-i275.

Tepeli YI, Ünal AB, Akdemir FM, Tastan O. (2020). Pamogk: A pathway graph kernel based multi-omics approach for patient clustering. *Bioinformatics*.

Thrun S, Saul LK, Schölkopf B. (2004). *Advances in Neural Information Processing Systems 16: Proceedings of the 2003 Conference* (Vol. 16): MIT press.

Valdeolivas A, Tichit L, Navarro C, Perrin S, Odelin G, Levy N*, et al.* (2019). Random walk with restart on multiplex and heterogeneous biological networks. *Bioinformatics, 35*(3), 497-505.

Vaske CJ, Benz SC, Sanborn JZ, Earl D, Szeto C, Zhu J*, et al.* (2010). Inference of patient-specific pathway activities from multi-dimensional cancer genomics data using PARADIGM. *Bioinformatics, 26*(12), i237-i245.

Wang B, Mezlini AM, Demir F, Fiume M, Tu Z, Brudno M*, et al.* (2014). Similarity network fusion for aggregating data types on a genomic scale. *Nature methods, 11*(3), 333.

Witten DM, Tibshirani R. (2010). A framework for feature selection in clustering. *Journal of the American Statistical Association, 105*(490), 713-726.

Yang Z, Michailidis G. (2016). A non-negative matrix factorization method for detecting modules in heterogeneous omics multi-modal data. *Bioinformatics, 32*(1), 1-8.

Yang Z, Xu J, Li L, Li R, Wang Y, Tian Y*, et al.* (2020). Integrated molecular characterization reveals potential therapeutic strategies for pulmonary sarcomatoid carcinoma. *Nature communications, 11*(1), 1-14.

Yuan Y, Savage RS, Markowetz F. (2011). Patient-specific data fusion defines prognostic cancer subtypes. *PLoS Comput Biol, 7*(10), e1002227.

Zhang S, Liu C-C, Li W, Shen H, Laird PW, Zhou XJ. (2012). Discovery of multi-dimensional modules by integrative analysis of cancer genomic data. *Nucleic acids research, 40*(19), 9379-9391.

Zitnik M, Zupan B. (2016). Jumping across biomedical contexts using compressive data fusion. *Bioinformatics, 32*(12), i90-i100.

Žitnik M, Zupan B. (2014). Data fusion by matrix factorization. *IEEE transactions on pattern analysis and machine intelligence, 37*(1), 41-53.

Žitnik M, Zupan B. (2015). Gene network inference by fusing data from diverse distributions. *Bioinformatics, 31*(12), i230-i239.
