# Supplementary material for: Unsupervised Multi-Omics Data Integration Methods: A Comprehensive Review
Source: Front Genet. 2022 Mar 22;13:854752. doi: 10.3389/fgene.2022.854752 (PMC8981526; doi:10.3389/fgene.2022.854752)
Supplement: Supplementary file 2 [file Table2.DOCX]

**Supplementary Materials**

*Unsupervised Multi-Omics Data Integration Methods: A Comprehensive Review*

Nasim Vahabi^1^, George Michailidis^1,*^

^1^Informatics Institute, University of Florida, Gainesville, FL 326011, USA.

*To whom correspondence should be addressed ([gmichail@ufl.edu](mailto:gmichail@ufl.edu)).

# S1 Multi-Omics Data

Using the term “Omics” beside a molecular term indicates a broad assessment of a set of molecules. These molecules are dynamically interacting with each other through the process of protein synthesis. In this section, we introduce these Omics disciplines, including genome, transcriptome, proteome, and metabolome) and multi-Omics (and single-Omics) data sources and tools. See Figure 1 (in the main text) for the molecular order of these Omics, potential between-layer and within-layer interactions, and the features available for each Omics type.

**Genome:** refers to the genome and sequenced DNA of a cell or organism that can be in the form of whole genome/exome sequences (WGS and WES) or individual DNA variations. Each human haploid genome contains three billion DNA points (also called DNA bases or DNA pairs) with about 1% protein-coding genes (about 20 000 genes). The remaining regions (about 99% of the genome) are called non-coding regions. The first complete whole-genome sequencing (human blueprint) is provided by the Human Genome Project (HGP) between 1990-2003 (<https://www.genome.gov/human-genome-project>), followed by the Encyclopedia of DNA elements (ENCODE, 2003, <https://www.encodeproject.org/>) to find the functional parts of these blueprints. About 99.5% of a person’s DNA is the same as any other (unrelated) person’s DNA. Differences in the DNA sequences between individuals (about 0.5% of DNA) are called genetic variation, including single nucleotide variation (SNP), structural variation (copy number variation – CNV, insertion, deletion, inversion), variable number tandem repeat (VNTR), Aneuploidy and indel (small insertion-deletion). SNP and CNV are the two most studied variations at the DNA level. In general, there are two different sequencing technologies for the identification of these genetic variations, including targeted sequencing and next-generation sequencing – NGS (including WGS, WES)(Bewicke-Copley *et al.*, 2019; Griffiths *et al.*, 2005). In addition, there are several databases for genetic variation extraction, including Human Genetic Variation Database - HGV (<https://www.hgvd.genome.med.kyoto-u.ac.jp/>), Genome-Wide Repository of Associations Between SNPs and Phenotypes - GRASP (<https://grasp.nhlbi.nih.gov/>), National Center for Biotechnology Information - NCBI (<https://www.ncbi.nlm.nih.gov/>), PhenoScanner (<http://www.phenoscanner.medschl.cam.ac.uk/>), the European Bioinformatics Institute - EBI (<https://www.ebi.ac.uk/>), deep catalog of Human Genetic Variation - 1000 Genomes (<https://www.internationalgenome.org/>) and the Database of Genomic Variants – DGV (<http://dgv.tcag.ca/dgv/>). For more detail, refer to (Sun and Hu, 2016).

**Transcriptome:** is the complete set of coding (1-4%, called messenger RNA - mRNA) and non-coding (≥96%, including small nuclear & interfering, micro & long non-coding and ribosomal RNA) RNA transcripts within a cell or an organism(Champe *et al.*, 2005). Transcriptomics analysis (mRNA is the most analyzed feature) enables the determination of up/down-regulated genes(Rapaport *et al.*, 2013), gene pattern(Yang *et al.*, 2014), and splice variants(Eswaran *et al.*, 2013) in various diseases. There are two different techniques to capture the transcriptome, i.e., RNA-microarray (based on the previous knowledge of the genome – fixed probs, less expensive(Shendure, 2008)) and RNA-sequencing (more complex discoveries – any sequence or organism, more expensive(Nagalakshmi *et al.*, 2010)). Several transcriptome databases are available, including ENSEMBL Biomart (<https://www.ensembl.org/biomart/>), Gene Expression Omnibus – GEO (<https://www.ncbi.nlm.nih.gov/geo/>), and ArrayExpress (<https://www.ebi.ac.uk/arrayexpress/>). For more detail, refer to Manzoni et al., 2018(Manzoni *et al.*, 2018).

**Proteome:** is the complete set of proteins in a cell, tissue, or organism. Proteome analysis consists of six main objectives, i.e., proteome mining, protein expression profiling, post-translational modifications, structural proteomics, functional proteomics, and protein-protein interactions (PPIs)(Graves and Haystead, 2002). Omics data complexity increases by moving from genome and transcriptome level to the proteome level since proteins are not easily replicated like DNA and have a dynamic (highly abundant) nature that greatly depends on both samples (samples’ heterogeneity) and sampling conditions. The leading approaches for complete plasma/serum profiling and protein detection/characterization are based on mass spectrometry (MS), which also is challenging due to MS-bias and its dependency on a proteome reference(Bell *et al.*, 2009) (such as UniProt's human proteome, <https://www.uniprot.org/>). For more detail, refer to (Timp and Timp, 2020).

**Metabolome:** is the complete set of small molecular metabolites in cells or organisms that contribute to metabolic reactions. In metabolomics studies, changes in metabolite structure/concentration (as a reaction to a set of stimuli from endogenous/exogenous components) enable the assessment of potential biomarkers(Fiehn *et al.*, 2000). In general, there are two metabolomics approaches, i.e., targeted (limited to known metabolites, analytical tools include low-resolution triple quadrupole MS - TQMS) and non-targeted (broad coverage for any metabolites, analytical tools include high-resolution MS - HRMS) methods. There are also some methods that benefit from both approaches by re-analyzing the detected metabolites from HRMS in the TQMS mode(S. Chen *et al.*, 2013). Several metabolomic databases are available, including human metabolome database - HMDB (<https://hmdb.ca/>), MetaboLights (<https://www.ebi.ac.uk/metabolights/index>), METLIN (<https://metlin.scripps.edu/>), and BiGG (<http://bigg.ucsd.edu/>), as well as several metabolic pathway databases (including Kyoto Encyclopedia of Genes and Genomes – KEGG, <https://www.genome.jp/kegg/>, Reactome, <https://reactome.org/>, and MetaCyc, <https://metacyc.org/>). For more detail, refer to Liu et al., 2020(Liu *et al.*, 2019).

**Quantitative Trait Loci (QTL):** Genome-wide QTL mapping enables the determination of genetic loci (locations) affecting other Omics, i.e., transcriptome, proteome, and metabolome. Some Omics markers do not directly contribute to the outcome (disease occurrence or disease severity) and affect the disease through other Omics (intermediates). Therefore, identifying these associations in each Omics layer can provide more functional information about disease-associated markers.

Gene expression QTL **- eQTL** (SNP-Gene relationship) - mapping helps understand the relationship between disease and genetic loci via gene expression regulation(Schadt *et al.*, 2005). Romanoski et al. showed that over 30% of gene transcripts are significantly affected by eQTL(Romanoski *et al.*, 2010). GTEx(Consortium, 2015) (Genotype-Tissue Expression) Consortium is one of the significant research groups that collect (and analyze) tissue-specific transcriptome (including mRNA, rRNA, tRNA, and lncRNA) and genome-wide genetic variation as well as the QTL data (<https://gtexportal.org/home/>). QTLbase (<http://mulinlab.org/qtlbase>), ExSNP (<http://www.exsnp.org/>), PhenoScanner (<http://www.phenoscanner.medschl.cam.ac.uk/>), and GRASP (<https://grasp.nhlbi.nih.gov/>) are other available QTL databases.

Methylation QTL – **meQTL** (SNP-CpG relationship) - are locations (sites) where genetic variations (such as genotypes) influence DNA methylation resulting in different DNA methylation patterns (allele-specific) across a genomic region(Smith *et al.*, 2014). Roadmap Epigenome Project (<http://www.roadmapepigenomics.org/>) has one of the largest human reference epigenome data collections that makes the meQTL data available. Pancan-meQTL(Gong *et al.*, 2019) (<http://gong_lab.hzau.edu.cn/Pancan-meQTL/>) is also one of the recently introduced databases for meQTL data in cancer (including cis-meQTL and trans-meQTL).

Protein QTL – **pQTL** (SNP-Protein relationship) – is based on the association between genetic variant and protein. Sun et al., 2018(Sun *et al.*, 2018) showed that about half of the pQTLs in their study overlapped with eQTL and disease-associated loci, implying that the genetic variants’ effect on protein abundance can be driven by regulation of mRNA. PhenoScanner (<http://www.phenoscanner.medschl.cam.ac.uk/>), GRASP (<https://grasp.nhlbi.nih.gov/>), and QTLbase (<http://mulinlab.org/qtlbase>) are examples of pQTL databases.

Metabolite QTL – **mQTL** (SNP-Metabolite relationship) – indicates the loci (QTL) that influence metabolite abundance. The level of metabolite concentration is indirectly associated with protein expression and genetic loci, where some of them overlapped with GWAS loci. Several studies showed that mQTL could provide a functional link between a disease-associated genetic variant and disease (Kelly *et al.*, 2018; Labadie *et al.*, 2020; Vallarino *et al.*, 2019). mQTL data are available on PhenoScanner (<http://www.phenoscanner.medschl.cam.ac.uk/>) and QTLbase (<http://mulinlab.org/qtlbase>) portals. For more detail about single-Omics technologies and studies, including QTL studies, refer to Sun and Hu, 2016(Sun and Hu, 2016).

A list of public multi-Omics data sources/repositories is provided in Supplementary Table S1. For a detailed description of some of these repositories, refer to Subramanian et al., 2020(Subramanian *et al.*, 2020).

**Table S1.** Multi-Omics public data sources and repositories

| **Database** | **Link** |
| --- | --- |
| 1000 Genome | <https://www.genome.gov/27528684/1000-genomes-project> |
| ADNI (Alzheimer’s Disease Neuroimaging Initiative) | <http://adni.loni.usc.edu/> |
| APID (Agile Protein Interactomes DataServer)^*^ | <http://cicblade.dep.usal.es:8080/APID/init.action> |
| ARCHS^4^ | <https://maayanlab.cloud/archs4/> |
| ArrayExpress^[27]^ | <https://www.ebi.ac.uk/arrayexpress/> |
| BioGRID | <https://thebiogrid.org/> |
| CCLE (Cancer Cell Line Encyclopedia) | <https://portals.broadinstitute.org/ccle> |
| ChEBI (Chemical Entities of Biological Interest) | <https://www.ebi.ac.uk/chebi/> |
| CPTAC (Clinical Proteomic Tumor Analysis Consortium) | <https://proteomics.cancer.gov/programs/cptac> |
| dbSNP | <https://www.ncbi.nlm.nih.gov/snp/> |
| DoRiNA 2.0 (a database of RNA interactions in post-transcriptional regulation)^*^ | <https://dorina.mdc-berlin.de/> |
| ENCODE (Encyclopedia of DNA Elements) | <https://www.encodeproject.org/> |
| GeneBank | <https://www.ncbi.nlm.nih.gov/genbank/> |
| GEO (Gene Expression Omnibus) | <https://www.ncbi.nlm.nih.gov/geo/> |
| GO Consortium | <http://geneontology.org/> |
| GTEx (Genotype-Tissue Expression) | <https://www.gtexportal.org/home/> |
| GWAS Catalog (The NHGRI-EBI Catalog of Human Genome-Wide Association Studies) | <https://www.ebi.ac.uk/gwas/> |
| HMDB^[28]^ (Human Metabolome DataBase) | <https://hmdb.ca/> |
| ICGC (International Cancer Genomics Consortium) | <https://icgc.org/> |
| inBio map^*^ | <https://inbio-discover.com/> |
| IntAct^*^ | <https://www.ebi.ac.uk/intact/> |
| KEGG (Kyoto Encyclopedia of Genes and Genomes) | <https://www.genome.jp/kegg/> |
| MetaboLights | <https://www.ebi.ac.uk/metabolights/> |
| modENCODE (Model Organism ENCyclopedia Of DNA Elements) | <http://modencode.org/> |
| NIH-ROADMAP | <http://www.roadmapepigenomics.org/> |
| OmicsDI (Omics Discovery Index) | [https://www.omicsdi.org](https://www.omicsdi.org/) |
| OMIM (Online Mendelian Inheritance in Man) | <https://www.omim.org/> |
| Pathway Commons^*^ | <https://www.pathwaycommons.org/> |
| PMN (Plant Metabolic Network) | <https://plantcyc.org/> |
| PRIDE (PRoteomics Identification Database) | <https://www.ebi.ac.uk/pride/> |
| RefSeq (The Reference Sequence) | <https://www.ncbi.nlm.nih.gov/refseq/> |
| STRING^*^ | <https://string-db.org/> |
| TARGET (Therapeutically Applicable Research to Generate Effective Treatments) | <https://ocg.cancer.gov/programs/target> |
| TCGA (The Cancer Genome Atlas Program) | <https://cancergenome.nih.gov/> |
| The Human Protein Atlas | <https://www.proteinatlas.org/> |

# S2 Feature Selection Methods

The goal of feature selection is to select a sparse model by identifying the subset of genuinely relevant features to the outcome. Sparsity, particularly in high-dimensional settings ($n<<p$), is a desirable property that can help to improve statistical stability and interpretability. In fact, it is mainly incorrect to fit an unregularized model in high-dimensional settings. To address this issue, there are generally two different adaptions: (A) leveraging various variable selection methods -stepwise approaches, or regularization methods (see (Bühlmann *et al.*, 2013) and references therein)-, (B) reducing the dimensionality of the predictors by creating new features (super-features) that are the combination of all the original features (such as principal components analysis(Jolliffe, 1986), or partial least squares(Wold *et al.*, 1983)). A number of methods - based on both approaches - have been developed and applied for Omics data analysis (see Table S2), including (1) Traditional feature selection(Bühlmann *et al.*, 2013) (including univariate selection and stepwise selection), (2) $L_{P}$-regularization of regression coefficients (such as ridge(Hoerl *et al.*, 1975), least absolute shrinkage and selection operator - lasso(Tibshirani, 1996), and elastic-net(Zou and Hastie, 2005)) or the inverse covariance matrix (covariance-regularized regression or scout(Witten and Tibshirani, 2009)), (3) principal component analysis(Jolliffe, 1986) (PCA), (4) canonical correlation analysis(Dolédec and Chessel, 1994) (CCA), (5) co-inertia analysis(Hotelling, 1992) (CIA), (6) multivariate factor analysis(Escofier and Pagès, 1998) (MFA), (7) partial triadic analysis(Kroonenberg, 2008) (PTA), (8) partial least square(Wold *et al.*, 1983) (PLS), (9) random forest(Breiman, 2001) (RF), and (10) Bayesian variable selection(Mitchell and Beauchamp, 1988) (BVS).

For the rest of this section, let $y\in R^{n}$ denotes the vector of $n$ response variables, $X\in R^{n\times p}$ denotes a matrix of $p$ features (covariates) measured for $n$ samples. Let define the ordinary least square (OLS) regression of $y$ on $X$ as $y=X\beta+\epsilon$, where $\beta\in R^{p}$ and $\epsilon\in R^{n}$ indicate the vector of regression coefficients and independent random errors, respectively. In the high-dimensional settings, the number of samples is smaller than the number of features ($n<p$).

**Table S2. Feature selection methods**. Let $y\in R^{n}$ denotes the vector of $n$ response variables, $X\in R^{n\times p}$ denotes a matrix of $p$ features (covariates) measured for $n$ samples. Let define the ordinary least square (OLS) regression of $y$ on $X$ as $y=X\beta+\epsilon$, where $\beta\in R^{p}$ and $\epsilon\in R^{n}$ indicate the vector of regression coefficients and independent random errors, respectively. “*LossFun*”, and “*ObjFun*” are the abbreviations for “loss function”, and “objective function”, respectively.

| **Method** | **Rule** | **Representation** | **Variations** | **Packages** |
| --- | --- | --- | --- | --- |
| **Traditional methods**(Bühlmann *et al.*, 2013) | *LossFun (λ)* | These methods select features based on statistical tests to determine the features' relationship's strength with a given outcome. The procedure starts with either the full or the reduced model and either eliminate or adds the features to the start model. The final decision is made by minimizing the sum of squared error. | - Univariate selection - FS(Draper and Smith, 1998; Efroymson, 1966) (Forward selection) - BE(Efroymson, 1966) (Backward elimination) - BSS(Beale *et al.*, 1967; Hocking and Leslie, 1967) (Best subset selection) - BSS using MIO(Bertsimas *et al.*, 2016) (Mixed-integer optimization) - RFE(Guyon *et al.*, 2002) (Recursive feature elimination) | - *stats(Team, 2013)* - *MASS(Ripley et al., 2013)* - *leaps(Lumley and Lumley, 2013)* - *caret(Kuhn, 2008)* - *glmnet(Friedman et al., 2010b)* - *olsrr(Hebbali and Hebbali, 2017)* |
| **Ridge**(Hoerl *et al.*, 1975) | *LossFun (λ)* | Minimizes the loss function by adding $L_{2}-norm$ constraint on the regression coefficients:  ${argmin}_{\beta}{\Vert y-X\beta\Vert}_{2}^{2}+ \lambda{\Vert\beta\Vert}_{2}^{2}.$  This method only shrinks the coefficients towards zero. | - GRridge^(Van De Wiel^ *^et al.^*^, 2016)^ (Adaptive group-regularized ridge regression) | - *caret(Kuhn, 2008)* - *glmnet(Friedman et al., 2010b)* - *GRridge(Van De Wiel et al., 2016)* - *lmridge(Ullah et al., 2018)* - *mlr(Bischl et al., 2016)* |
| **Lasso**(Tibshirani, 1996)  ^(Least absolute shrinkage and selection)^ | *LossFun (λ)* | In contrast to the ridge, lasso aims to simultaneously shrink and select a subset of variables through an $L_{1}-norm$ constraint on the regression coefficients:  ${argmin}_{\beta}{\Vert y-X\beta\Vert}_{2}^{2}+\lambda{\Vert\beta\Vert}_{1},$  where $\lambda>0$ is the penalty parameter. | - ALasso(Zou, 2006) (Adaptive lasso) - Relaxed lasso(Meinshausen, 2007) - SEA-lasso(Qian and Yang, 2013) (Standard error adjusted adaptive lasso) - Priority-lasso(Klau *et al.*, 2018) - Group lasso(Yuan and Lin, 2006) - Sparse group lasso(Friedman *et al.*, 2010a) - SGL(Simon *et al.*, 2013) (Sparse group lasso) - gLASSO(Friedman *et al.*, 2008) (Graphical lasso) - MGM(Lee and Hastie, 2013) (Mixed graphical models) - Fused lasso(Tibshirani *et al.*, 2005) - GFlasso(Chen *et al.*, 2010) (Graph-guided fused lasso) - wgLASSO(Li and Jackson, 2015) (Weighted graphical lasso) - IPF-Lasso(Boulesteix *et al.*, 2017) (Integrative lasso with penalty factors) - OSCAR(Bondell and Reich, 2008) (Octagonal shrinkage and clustering) | - *caret(Kuhn, 2008)* - *glmnet(Friedman et al., 2010b)* - *pec(Mogensen et al., 2012)* - *SGL(Simon et al., 2018)* - *psych(Revelle and Revelle, 2015)* - *mvtnorm(Genz et al., 2020)* - *grpreg(Breheny and Breheny, 2020)* (grouplasso) - *mlr(Bischl et al., 2016)* |
| **El-net**(Zou and Hastie, 2005)  ^(Elastic-net)^ | *LossFun* $\alpha,\lambda$*)* | Minimizes the loss function by adding both $L_{1}$ and $L_{2}$ constraints on the coefficients:  ${argmin}_{\beta}\frac{1}{2n}{\Vert y-X\beta\Vert}_{2}^{2}+\lambda\left[ \frac{1}{2}\left( 1-\alpha\right){\Vert\beta\Vert}_{2}^{2}+\alpha{\Vert\beta\Vert}_{1} \right],$  where $\lambda>0$ and $\alpha\in\left[ 0,1 \right]$ are penalty parameters. When $\alpha=0$ and $\alpha=1$, this reduces to the ridge and lasso, respectively. | - CEN(Price and Sherwood, 2017) (Cluster elastic net) - Scout(Witten and Tibshirani, 2009) (Covariance-regularized regression)^*^ - Grace(Li and Li, 2010) (Graph-constrained estimate) - aGrace(Li and Li, 2010) (Adaptive grace) | - *caret(Kuhn, 2008)* - *glmnet(Friedman et al., 2010b)* - *ensr(DeWitt and Bennett, 2019)* - *mlr(Bischl et al., 2016)* |
| **PCA**(Jolliffe, 1986)  ^(Principal component analysis)^ | *ObjFun (*Max Correlation) | Computes a collection of $v<n$ new features (orthogonal latent components) as linear combinations of the original features (in a single-datatype setting). PCA only considers $X$ to construct the super features by maximizing the following objective function (variance of data, $X$):  ${argmax}_{w^{k}}{\Vert Xw^{k}\Vert}_{2}^{2}, for k=1,\ldots,v$  $subject to \Vert w^{k}\Vert=1,$  where $v$ is the number of components and $w^{k}$ is the weight (loading) for the $k^{th}$ component. The component $T^{k}$ is then calculated as $T^{k}=Xw^{K}$. | - SPCA(Zou *et al.*, 2006) (Sparse PCA) - PPCA(Tipping and Bishop, 1999) (Probabilistic PCA) - SCoTLASS(Jolliffe *et al.*, 2003) - SUN-PCA(Smilde *et al.*, 2003) - PCoA(Gower, 1966; Zuur *et al.*, 2007) (Principle coordinate analysis) - CPCA(Westerhuis *et al.*, 1998; Wold, 1987) (Consensus PCA) - GPCA(Casin, 2001) (Generalized PCA) - HPCA(Wold *et al.*, 1996) (Hierarchical PCA) - PCA-SIR(Tu *et al.*, 2015) (PCA-sliced inverse regression ) - MGPCA(Krzanowski, 1984) (Multi-group PCA) - cPCA(Abid *et al.*, 2018) (Contrastive PCA) - scPCA(Boileau *et al.*, 2020a) (Sparse contrastive PCA) - nsPCA(Zass and Shashua, 2006) (Nonnegative sparse PCA) - bPCA(Bishop, 1999) (Bayesian PCA) - GSPPCA(Bouveyron *et al.*, 2018) (Globally sparse probabilistic PCA) | - *ade4(Dray and Dufour, 2007)* - *FactoMineR(Lê et al., 2008)* - *Base R* - *PCAtools(Blighe et al., 2019)* - *scPCA(Boileau et al., 2020b)* - *stats(Team, 2013)* - *vegan(Oksanen et al., 2007)* - *psych(Revelle, 2011)* - *pcaMethods(Stacklies et al., 2007)* - *ape(Paradis et al., 2015)* |
| **CCA**(Hotelling, 1992)  ^(Canonical correlation analysis)^ | *ObjFun* (Max-Covariance) | An approach to analyze two-table data (${X=[X}_{n\times p_{1}}^{(1)}\vert X_{n\times p_{2}}^{\left( 2 \right)}]$) to find the low dimensional components with maximal covariance. The objective function is as follows:  ${argmax}_{u,v} ú\sigma_{X^{(1)}X^{(2)}} v$  $s.t. ú \sigma_{X^{(1)}X^{(1)}} u=v́ \sigma_{X^{(2)}X^{(2)}} v=1,$  where $\sigma$ indicates the covariance estimate. The components are then calculated as $T_{X^{(1)}}=X^{(1)}u$ and $T_{X^{(2)}}=X^{(2)}v$. | - rCCA(Cruz-Cano and Lee, 2014; Leurgans *et al.*, 1993) (Regularized CCA) - pCCA/PMDCCA(Witten *et al.*, 2009) (Penalized CCA/Penalized matrix decomposition CCA) - sCCA(Lykou and Whittaker, 2010) (Sparse CCA) - gCCA(Carroll, 1968) (Generalized CCA) - RGCCA(Tenenhaus and Tenenhaus, 2011; M. Tenenhaus *et al.*, 2017) (Regularized generalized CCA) - ssCCA(J. Chen *et al.*, 2013) (Structure-constrained sparse CCA) - CCA-EN(Waaijenborg *et al.*, 2008) (CCA with elastic-net) | - *ade4(Dray and Dufour, 2007)* - *cca(González et al., 2008)* - *vegan(Oksanen et al., 2007)* - *Base R* - *dmt(Lahti et al., 2013)* - *rgcca(A. Tenenhaus et al., 2017)* - *mixOmics(Rohart et al., 2017)* - *PMA(Witten et al., 2020)* |
| **CIA**(Dolédec and Chessel, 1994) | *ObjFunc* (Max-Covariance) | An approach to analyze two-table data (${X=[X}_{n\times p_{1}}^{(1)}\vert X_{n\times p_{2}}^{\left( 2 \right)}]$) to find orthonormal directions ($u$ and $v$**)**, by maximizing the covariance between the data tables:  ${argmax}_{u,v} ú X^{(1)}́X^{(2)} v,$  $s.t. \Vert u\Vert=\Vert v\Vert=1.$  The components are then calculated as $T_{X^{(1)}}=X^{(1)}u$ and $T_{X^{(2)}}=X^{(2)}v$.  The main difference between CCA and CIA is that the CCA objective function maximizes the correlation between data tables, while CIA maximizes the covariance. | - PCIA^(Min^ *^et al.^*^, 2019)^ (Penalized CIA) - MCIA(Meng *et al.*, 2014) (Multiple CIA) - sMCIA(Min and Long, 2020) (Sparse multiple CIA) | - *ade4(Dray and Dufour, 2007)* - *made4(Culhane et al., 2005)* - *omicade4(Meng et al., 2013)* - *cocorresp(Šmilauer et al., 2020)* |
| **MFA**(Escofier and Pagès, 1998)  ^(Multiple Factor Analysis)^ | *ObjFun (*Max Correlation) | An extension of PCA to analyze multiple-table data (${X=[X}_{n\times p_{1}}^{(1)}\vert\ldots\vert X_{n\times p_{B}}^{(B)}$]) in two steps:  *Step1.* PCA on each data-table and collect the matrix of weights ($w$).  *Step2.* Generalized PCA on $X$ using the weights ($w$) to compute the factor scores (factor loadings) via generalized singular value decomposition (GSVD) of $X$:  $X=P\Delta Q^{T},$  $s.t. P^{T}MP=Q^{T}AQ=I,$  where $M_{n\times n}$ and $A_{p\times p}$ are positive define matrices, $\Delta_{r\times r}$ is the diagonal matrix of the $r$ generalized singular values, where $r$ is the rank of $X$. $P_{n\times r}$ and $Q_{n\times r}$ are matrices of the normalized generalized left and right singular vectors, respectively. | - HMFA(Le Dien and Pagès, 2003) (Hierarchical MFA) - PMFA(Morand and Pagès, 2006) (Procrustes MFA) - DMFA(Lê and Pagès, 2010) (Dual MFA) - MUFABADA(Abdi *et al.*, 2012) (MFA barycentric discriminant analysis) | - *FactoMineR(Lê et al., 2008)* - *SensoMineR(Lê and Husson, 2008)* - *factoextra(Kassambara and Mundt, 2017)* - *AFMULT(Escofier and Pages, 1994)* |
| **PTA**(Kroonenberg, 2008)  ^(Partial Triadic Analysis)^ | *ObjFun* (Max-Correlation) | PTA is a two-step approach to analyze multiple-table data (${X=[X}_{n\times p_{1}}^{(1)}\vert\ldots\vert X_{n\times p_{B}}^{\left( B \right)}]$) when each data type has the same dimension ($p_{1}=p_{2}=\ldots=p_{B}$):  *Step1.* Combine the $B$ data tables into a single data matrix ($X_{n\times p}$). A naïve approach to do so is to average each sample (row) across the $B$ data tables. However, PTA up-weights the data tables which are closer to the combined (average) data matrix as $X=\sum_{b=1}^{B} \alpha^{(b)}X_{..}^{(b)}$, where $\alpha$ is chosen to maximize the following objective function:  ${argmax}_{\alpha}\sum_{b=1}^{B} \alpha^{(b)}{{(X}_{..}^{(b)}}^{T}\underline{X}),$  $s.t. {\Vert\alpha\Vert}_{1}=1,$  where $\underline{X}=\frac{1}{B}\sum_{b=1}^{B} X_{..}^{(b)}$.  *Step2.* Apply standard methods, such as PCA, on the combined data matrix ($X$). | - | - *ade4(Dray and Dufour, 2007)* |
| **NMF**(Lee and Seung, 2001) ^(Nonnegative Matrix Factorization)^ | *ObjFun (*Max Correlation) | Similar to PCA, NMF computes a collection of $v<n$ latent components ($T$), except that it employs the non-negativity constraint on the data:  ${argmin}_{w,T}{\Vert X-wT\Vert}_{F}^{2},$  $s.t. w,T\geq0,$  where ${\Vert.\Vert}_{F}$ is a Frobenius norm, and $w$ is a $p\times v$ matrix of weights. | - CNMF(Brunet *et al.*, 2004) - jNMF (Zhang *et al.*, 2012) (Joint NMF) - iNMF (Yang and Michailidis, 2016) (Integrative NMF) - iONMF(Stražar *et al.*, 2016) (Integrative, orthogonality regularized nonnegative matrix factorization) | - *NMF(Gaujoux and Seoighe, 2010)* - *NNLM(Lin and Boutros, 2019)* - *ccFindR(Woo et al., 2019)* |
| **PLS**(Wold *et al.*, 1983) ^(Partial least squares)^ | *ObjFunc* (Max-Covariance) | Similar to PCA, PLS computes a collection of $v<n$latent components ($T$), except that it uses both $X$ and $y$ to construct the components by maximizing the following objective function:  $w_{k}={argmax}_{w_{k}}w_{k}́X́yýXw_{k}, for k=1,\ldots,v$  $s.t. {\Vert w\Vert}_{2}=1,$  where $v$ is the number of latent components, $w_{k}$ is the weight vector related to the $k^{th}$ component ($k=1,\ldots,v$), and $W$ is a $p\times v$ matrix of all weights. The latent component $T$ is then calculated as $T=Xw$. | - WA-PLS(Ter Braak *et al.*, 1993) (Weighted averaging PLS) - sPLS(Chun and Keleş, 2010; Lê Cao *et al.*, 2008) (Sparse PLS) - Correlation-based penalty(Tutz and Ulbricht, 2009) - sPLS-DA(Lê Cao *et al.*, 2011) (Sparse PLS discriminant analysis) - O-PLS(Trygg and Wold, 2002) (Orthogonal PLS) - O2-PLS(Trygg, 2002) (Two-way orthogonal PLS) - pPLS(Huang and Pan, 2003) (Penalized PLS) - OnPLS(Löfstedt and Trygg, 2011) - On-PLS & MB-VIOP(Reinke *et al.*, 2018) (Multi-block variable influence on orthogonal projections) - K-OPLS(Rantalainen *et al.*, 2007) (Kernel-based PLS) - MBPLS(Wangen and Kowalski, 1989) (Multi-block PLS) - sMBPLS(Li *et al.*, 2012) (Sparse MBPLS) | - *pls(Wehrens and Mevik, 2007)* - *spls(Chung et al., 2012)* - *K-OPLS(Bylesjö et al., 2008)* - *mixOmics(Rohart et al., 2017)* - *OmicsPLS(el Bouhaddani et al., 2018)* - *STATegRa(Consortia, 2014)* |
| **RF**(Breiman, 2001)  ^(Random forest)^ | *LossFun* | RF is an ensemble method consisting of the aggregation of decision trees. Each tree is randomly drawn from data using the CART method and decreased Gini impurity (DGI) as the splitting rule. RF provides estimators of either the Bayes classifier or the regression function by minimizing the expected value of the loss function:  ${argmin}_{y} E_{Xy}\left[ L\left( y, f\left( X \right) \right) \right],$  where $f\left( X \right)$ is a collection of $J$ *learners* (trees), $l_{1}\left( X \right), \ldots, l_{J}\left( X \right)$, which are averaged as:  $f\left( X \right)=\{\frac{1}{J}\sum_{j=1}^{J} l_{j}\left( X \right) In regression {argmax}_{y} \sum_{j=1}^{J} I\left( y=l_{j}\left( X \right) \right) In classification$  Common choices for the loss function are squared error loss (for prediction) and zero-one loss (for classification). | - Block Forest(Hornung and Wright, 2019) - MERF(Hajjem *et al.*, 2014) (Mixed-effects RF) - RSF(Ishwaran *et al.*, 2008) (Random survival forest) - RF-RFE(Granitto *et al.*, 2006) (Random forest-recursive feature elimination) | - *caret(Kuhn, 2008)* - *glmnet(Friedman et al., 2010b)* - *randomForest(Liaw and Wiener, 2002)* - *randomForestSRC(Ishwaran et al., 2020)* - *mlr(Bischl et al., 2016)* |
| **BVS**(Mitchell and Beauchamp, 1988)  ^(Bayesian Variable Selection)^ | *LossFun (λ)* | The regularization in BVS is commonly formulated via shrinkage priors under which the shrinkage priors are expressed as the so-called “scale-mixture" of normal and gamma distributions. For instance, in the Bayesian Lasso, the scale mixture priors are represented as:  $\pi\left( \beta\vert\sigma^{2},\tau_{1},\ldots,\tau_{p} \right)=\pi\left( 0, \sigma^{2}D_{\tau} \right)$,  $\pi\left( \tau_{1},\ldots,\tau_{p} \right)=\prod_{j=1}^{p} \frac{\lambda^{2}}{2}exp\left( -\lambda^{2}\tau_{j}^{2}/2 \right),$  where $D_{\tau}=diag\left( \tau_{1}^{2},\ldots,\tau_{p}^{2} \right)$ , and $\lambda$ is a tuning parameter. | - MRF(Li and Zhang, 2010) (Markov random field) - Joint(Peterson *et al.*, 2016) - HB-HHSVM(Chakraborty and Guo, 2011) (Bayesian hybrid huberized SVM) - Spatial BVS(Smith and Fahrmeir, 2007) - Bayesian-lasso(Hans, 2009; Park and Casella, 2008) - Bgla(Wang, 2012) (Bayesian graphical lasso) - Bayesian fused lasso(Kyung *et al.*, 2010) - Bayesian elastic-net(Hans, 2011; Kyung *et al.*, 2010) - pLasso(Wang *et al.*, 2013) (Prior lasso) - EMVS(Ročková and George, 2014) (Expectation-maximization approach to BVS) - Joint BVS and graph estimation(Sun *et al.*, 2020) | - *mvtnorm(Genz et al., 2020)* - [*gamlss.spatial*](https://www.rdocumentation.org/packages/gamlss.spatial/versions/2.0.0)*(De Bastiani et al., 2018)* - *MRFcov(Clark et al., 2018)* - *mgcv(Wood and Wood, 2015)* - *spikeSlabGAM(Scheipl and Scheipl, 2020)* - *BayesVarSel(Garcia-Donato et al., 2015)* - *EBglmnet(Huang and Liu, 2016)* - *BayesSUR(Zhao et al.)* - *EBglmnet(Huang and Liu, 2016)* |

**Traditional** variable selection methods (dating back to about 1966) include univariate selection, forward selection(Draper and Smith, 1998; Efroymson, 1966), backward elimination(Efroymson, 1966), and best subset selection(Beale *et al.*, 1967; Hocking and Leslie, 1967). These methods are based on evaluating the regression coefficients (the effect of the features on the outcome) against zero. These methods select features based on statistical tests to determine the strength of the relationship between features and a given outcome. The procedure starts with either the full or the reduced model and either eliminates or adds the features to the start model. The final decision is made by minimizing the sum of squared error. For instance, in the backward elimination(Efroymson, 1966) method, the procedure starts with a multiple regression model built with all features (covariates, $X$). Then nonsignificant features are sequentially eliminated from the model, and the model is re-estimated until all remaining features have a significant association with the outcome ($y$). In 2002, recursive feature elimination (RFE), or SVM-RFE (support vector machine RFE), was introduced as an iterative algorithm that generates the features’ ranks by eliminating one backward feature at a time. Some extensions of RFE include EnRFE(Chen and Jeong, 2007) (enhanced RFE), SVM-RFE with mRMR(Mundra and Rajapakse, 2007) (minimum redundancy maximum relevance(Ding and Peng, 2005)), and SVM-RFE with correlation(Yoon and Kim, 2009), which modified the ranking criteria in the original RFE. Best subset selection(Beale *et al.*, 1967; Hocking and Leslie, 1967) (BSS) finds a subset of $k$ features ($k<n$) that minimize the loss function (sum of squared errors) subject to ${\|\beta\|}_{0}<k$, as follows:

$${argmin}_{\beta}{\|y-X\beta\|}_{2}^{2}, s.t. {\|\beta\|}_{0}<k,$$

where ${\|\beta\|}_{0}$ is the $L_{0}-norm$ of the regression coefficients (${\|\beta\|}_{0}=\sum_{i=1}^{p} 1\left\{ \beta_{i}\neq0 \right\}$). BSS via MIO(Bertsimas *et al.*, 2016) (mixed-integer optimization) is a recent extension of BSS, which allows the use of MIO-solver to minimize the loss function. Simulation studies(Bertsimas *et al.*, 2016; Hastie *et al.*, 2017) showed that BSS is superior to stepwise (forward or backward) and lasso variable selections.

**The ridge**(Hoerl *et al.*, 1975) minimizes the loss-function by replacing the $L_{0}-norm$ with $L_{2}-norm$, which can be written as:

$${argmin}_{\beta}{\|y-X\beta\|}_{2}^{2}+ \lambda{\|\beta\|}_{2}^{2},$$

where $\lambda$ is a tuning parameter ($\lambda\geq0$). **The lasso**(Tibshirani, 1996) minimizes the loss-function by utilizing the $L_{1}-norm$ of the regression coefficients as ${argmin}_{\beta}{\|y-X\beta\|}_{2}^{2}+\lambda{\|\beta\|}_{1}$. However, ridge regression only shrinks the coefficients towards zero. Instead, the lasso method aims to simultaneously shrink and select a subset of variables through an $L_{1}-norm$ constraint on the regression coefficients. An important limitation of the lasso method, especially in the case of Omics data, is that lasso tends to select only one variable among a group of correlated variables. For instance, there are many features in the multi-Omics framework that interact as a network (or module) and share the same biological pathway. Therefore, the lasso method can poorly indicate this grouping information in the multi-Omics setting. Theoretical and practical explanations of this limitation are given in Efron et al., 2004(Efron *et al.*, 2004), and Zou and Hastie, 2005(Zou and Hastie, 2005). To address these limitations, **the elastic-net**(Zou and Hastie, 2005) (el-net) was introduced by imposing a convex combination of the lasso and ridge ($L_{1}-L_{2}$) penalties on the regression coefficients. An extension of the LARS algorithm (Efron *et al.*, 2004) (LARS-EN (Zou and Hastie, 2005)) was then used to solve the elastic-net with the same computational complexity as the OLS method. There are various extensions of the ridge (including adaptive group regularized ridge(Van De Wiel *et al.*, 2016)), lasso (including fused lasso(Tibshirani *et al.*, 2005), adaptive lasso(Zou, 2006), group lasso(Yuan and Lin, 2006), graphical lasso(Friedman *et al.*, 2008) and priority lasso(Klau *et al.*, 2018)) and el-net (including cluster elastic-net(Price and Sherwood, 2017)) which can be found in Table S2.

**PCA** is one of the classic dimensionality reduction and visualization methods that computes a collection of $v<n$ new features, so-called super-features (orthogonal latent components, also called PCs), linear combinations of the original features. PCA only considers $X$ to project the high-dimensional data into the super features by maximizing the sample covariance between the features ($X$):

$${argmax}_{w^{k}}{\|Xw^{k}\|}_{2}^{2}, for k=1,\ldots,v$$

$$subject to \|w^{k}\|=1,$$

where $v$ is the number of components, and $w$ is a $p\times v$ matrix of weights. The component $T$ (a $n\times v matrix$) is then calculated as $T=Xw$. Different algorithm(Meng *et al.*, 2016) can be used to compute the PCs, including eigenanalysis, factor analysis (FA), and singular value decomposition (SVD). However, in the high-dimensional setting, PCA may fail to consistently assess the true super-features with maximal variability(Meng *et al.*, 2016; Sill *et al.*, 2015). Moreover, PCA was initially introduced for Gaussian data ($X\sim Normal\left( M,\Sigma\right))$, and can be impacted in the presence of strong skewness or outliers. Horseshoe effect(Legendre and Legendre, 2012) is another limitation of PCA in the presence of unimodal data and/or nonlinearity between the features. Another important drawback of PCA is that each super-feature (PC) is a linear combination of all original features, making its interpretability complicated. One simple (but misleading(Cadima and Jolliffe, 1995)) way to deal with the interpretability issues is to ignore all the features with coefficients less than a fixed threshold (consider them as zero) and only interpret the remaining features in the PC. SCoTLASS(Jolliffe *et al.*, 2003), Sparse PCA(Zou *et al.*, 2006) (SPCA), and non-negative sparse PCA(Zass and Shashua, 2006) (nsPCA) are three extensions of PCA that modify the PCs by imposing an extra lasso-based penalty on the sum of the absolute values of the latent loadings ($\sum_{i=1}^{p} w_{i}\leq\lambda$). Multi-group PCA(Krzanowski, 1984) (MGPCA), consensus PCA(Westerhuis *et al.*, 1998) (CPCA), hierarchical PCA(Wold *et al.*, 1996) (HPCA), generalized PCA(Casin, 2001) (GPCA), and contrastive PCA(Abid *et al.*, 2018) (cPCA) are another extensions of PCA to analyze the hierarchical (grouped) or multiple datasets. The Bayesian version of sparse PCA is also recently introduced as the globally sparse probabilistic PCA(Bouveyron *et al.*, 2018) (GSP-PCA).

**CCA**(Hotelling, 1992) is an approach to find the low-dimensional components (super-features) in the multiple-tables settings where ${X=[X}_{n\times p_{1}}^{(1)}|\ldots|X_{n\times p_{B}}^{\left( B \right)}]$. In the conventional CCA, suppose $B=2$ (i.e., there are two sets of data measured on the same $n$ samples), the components (super-features) are computed by maximizing the covariance between linear combinations of the data $X^{(1)}$ and $X^{(2)}$ (a cross-product matrix):

$${argmax}_{u,v} ú\sigma_{X^{(1)}X^{(2)}} v, s.t. ú \sigma_{X^{(1)}X^{(1)}} u=v́ \sigma_{X^{(2)}X^{(2)}} v=1,$$

where $\sigma$ indicates the covariance estimate. This method enables us to simultaneously analyze multiple datasets instead of considering them separately. However, when the number of data tables ($B$) is large compared to the sample size ($n$), the calculation of $\sigma^{-1}$ becomes complicated or impossible. There are several extensions of CCA for more than two datasets ($B\geq3)$ and/or high dimensional data, including regularized CCA(Cruz-Cano and Lee, 2014; Leurgans *et al.*, 1993) (rCCA), generalized CCA(Hanafi *et al.*, 2011) (gCCA) and regularized generalized CCA(Tenenhaus and Tenenhaus, 2011) (RGCCA). Penalized CCA(Witten *et al.*, 2009) (pCCA), sparse CCA(Lykou and Whittaker, 2010) (sCCA) and CCA with el-net(Waaijenborg *et al.*, 2008) (CCA-EN) are also sparse versions of CCA via applying a penalized matrix decomposition (PMD) to the cross-product matrix of data.

**CIA**(Dolédec and Chessel, 1994; Dray *et al.*, 2003) (also called co-inertia analysis, CoIA) is another method to find the low-dimensional components in two-table data settings where ${X=[X}_{n\times p_{1}}^{(1)}|X_{n\times p_{2}}^{\left( 2 \right)}]$. This method was first introduced in ecology to link species abundance with environmental features. Orthonormal directions ($u$ and $v$**)** are computed by maximizing the covariance between the data tables:

$${argmax}_{u,v} ú X^{(1)}́X^{(2)} v, s.t. \|u\|=\|v\|=1,$$

CIA can be considered as a variation of CCA(Sankaran and Holmes, 2019), and the only difference is in CIA the norm constraint ($\|u\|=\|v\|=1$) is directly applied on the orthonormal directions ($u$ and $v$**)** instead of a transformation of them (including the covariance). It is shown that the features selected by CCA and PLS approaches are highly similar but marginally different from ones selected by CIA(Lê Cao *et al.*, 2009; Meng *et al.*, 2014). Multiple CIA(Meng *et al.*, 2014) (MCIA) is the extension of CIA (and CPCA as well) for the analysis of more than two data tables. Penalized CIA(Min *et al.*, 2019) (PCIA) and sparse MCIA(Min and Long, 2020) (sMCIA) are sparse versions of CIA to aid the interpretability by forcing some of the features in the direction vectors through zero.

**MFA**(Escofier and Pagès, 1998) is another variation of PCA designed to handle multiple table data where ${X=[X}_{n\times p_{1}}^{(1)}|\ldots|X_{n\times p_{B}}^{(B)}$]. MFA is a two-step procedure: (1) PCA on each data table ($X_{n\times p_{B}}^{(B)}$) and collect the matrix of weights ($w$), then (2) apply a generalized PCA on X using the weights matrix (w) to compute the factor scores (or factor loadings) via generalized singular value decomposition (GSVD) of $X$, as:

$$X=P\Delta Q^{T}, s.t. P^{T}MP=Q^{T}AQ=I,$$

where $M_{n\times n}$ and $A_{p\times p}$ are positive define matrices, $\Delta_{r\times r}$ is the diagonal matrix of the r generalized singular values, where r is the rank of $X$. $P_{n\times r}$ and $Q_{n\times r}$ are matrices of the normalized generalized left and right singular vectors, respectively. Hierarchical MFA(Le Dien and Pagès, 2003) (HMFA) is an extension of MFA for the settings where data have a hierarchical structure, and one wants to consider the role of the groups of features at each hierarchy as well as the marginal (overall) hierarchy. Dual MFA(Lê and Pagès, 2010) (DMFA) is another extension of MFA where there are structured samples, i.e., the same variables are measured on different sets of samples.

**PTA**(Kroonenberg, 2008) is a two-step method to analyze multi-table data (${X=[X}_{n\times p_{1}}^{(1)}|\ldots|X_{n\times p_{B}}^{\left( B \right)}]$) when each data type has the same number of features ($p_{1}=p_{2}=\ldots=p_{B}$), including (1) Combining the $B$ blocks into a single data block ($X_{n\times p}$). A naïve approach to do so is to average each sample (row) across the $B$ blocks; however, PTA up-weights the data blocks which are closer to the combined (average) data block as $X_{n\times p}=\sum_{b=1}^{B} \alpha^{(b)}X_{..}^{(b)}$, where $\alpha$ is chosen to maximize the objective function:

$${argmax}_{\alpha}\sum_{b=1}^{B} \alpha^{(b)}{{(X}_{..}^{(b)}}^{T}\underline{X}), s.t. {\|\alpha\|}_{1}=1,$$

where $\underline{X}=\frac{1}{B}\sum_{b=1}^{B} X_{..}^{(b)}$; and then (2) applying standard methods, such as PCA, on the combined data block ($X_{n\times p}$).

**NMF**(Lee and Seung, 2001), similar to PCA, computes a collection of $v<n$ latent components in a single-datatype setting ($X=X^{(1)}$), except that it employs the non-negativity constraint on the data:

$${argmin}_{w,T}{\|X-wT\|}_{F}^{2}, s.t. w\geq0, T\geq0,$$

where ${\|.\|}_{F}$ is a Frobenius norm, and $w$ is a $p\times v$ matrix of weights. NMF enables patterns, modules, and class discovery. Joint NMF(Zhang *et al.*, 2012) (jNMF) is an extension of NMF for multiple datatypes (${X=[X}_{n\times p_{1}}^{(1)}|\ldots|X_{n\times p_{B}}^{\left( B \right)}]$) and can be considered as a multiple-NMF. It minimizes the same objective function as NMF for each datatype with a shared matrix of weights ($w$) but a data-specific matrix of components ($T^{(b)}, b=1,\ldots,B$). Integrative NMF(Yang and Michailidis, 2016) (iNMF) is the next extension of NMF for multiple datatypes that also account for heterogeneity between the multiple datatypes by providing heterogenous estimations/combinations ($v_{b}T_{b}$), i.e., it has data-specific matrices of weights ($v_{b}$), and latent components ($T_{b}$).

**PLS**(Wold *et al.*, 1983), similar to PCA, is a method to compute a collection of $v<n$new features as linear combinations of the original features (called latent components, $T$). In contrast to PCA, PLS uses both $X$ and $y$ to construct the super features by maximizing the covariance between $y$ and the latent components, as follows:

$$w_{k}={argmax}_{w_{k}}w_{k}́X́yýXw_{k}, for k=1,\ldots,v$$

$$s.t. {\|w\|}_{2}=1,$$

where $v$ is the number of latent components (fixed by user), and $w$ is a matrix of weights (also called direction vector). This method is then extended for various generalized regression models, including Cox-PH(Cox, 1972) model (PLS-GLR)(Bastien and Tenenhaus, 2001; Bastien *et al.*, 2005; Garthwaite, 1994; Nguyen and Rocke, 2002; Nygård *et al.*, 2008). Shortly after these extensions, Cao(Lê Cao *et al.*, 2008) (2008) and Chun and Keles (2010)(Chun and Keleş, 2010) showed that a large number of features in the high-dimensional framework could greatly affect the prediction performance in PLS regression. Chun and Keles(Chun and Keleş, 2010) proposed the sparse PLS (sPLS) by incorporating a variable selection constraint directly on the PLS direction vectors. Sparse PLS discriminant analysis(Lê Cao *et al.*, 2011) (sPLS-DA) is an extension of sPLS for multiclass classification problems. However, the approaches mentioned above do not distinguish between variables obtained from different molecular compartments and ignore any biological constraints – e.g., many variables belong to the same functional pathway or act as regulators of other variables. A similar issue arises in complex chemical systems, where variables can be naturally grouped into blocks. Multi-Block PLS(Wangen and Kowalski, 1989) (MBPLS) was developed to study the association between a numerical outcome variable and blocks of a priori defined predictors. The algorithm estimates each block's model parameters and combines them using each block's relative importance in predicting the outcome variable. Orthogonal PLS(Trygg and Wold, 2002) (O-PLS) and two-way orthogonal PLS(Trygg, 2002) (O2-PLS) are two alternatives to PLS to remove the systematic variations in $X$ by separating the systematic variations in the outcome, $y$, from the feature structure via orthogonal signal correction(Wold *et al.*, 1998) (OSC). These methods capture the noise by making sure that the latent components ($T$) also become the PCs in $X$ ($Tw^{T}$). The same problem can be also taken care in the PLS method by increasing the number of latent components in the model, which increases the complexity of the model. Kernel-based orthogonal PLS(Bylesjö *et al.*, 2008) (K-OPLS) is an extension of O-PLS by replacing the $X$ with the kernel Gramian matrix. OnPLS(Löfstedt and Trygg, 2011) is an extension of O2-PLS to handle multi-block designs with more than two blocks (matrices) of data. On-PLS is then combined with multi-block variable influence on orthogonal projections(Reinke *et al.*, 2018) (MB-VIOP) to better identify the features with the highest contribution in the outcome.

**RF**(Breiman, 2001) has become a commonly used (and standard) non-parametric method for classification and prediction problems besides variable selection. RF is an ensemble method that consists of the aggregation of several decision trees. Each tree is randomly drawn from data using the CART method and the Decrease Gini Impurity (DGI) as the splitting rule. RF provides estimators of either the Bayes classifier or the regression function by minimizing the expected value of the following loss function:

$${argmin}_{y} E_{Xy}\left[ L\left( y, f\left( X \right) \right) \right],$$

where $f\left( X \right)$ is a collection of $J$ *learners* (trees), $l_{1}\left( X \right), \ldots, l_{J}\left( X \right)$, which are averaged as:

$$f\left( X \right)=\{\frac{1}{J}\sum_{j=1}^{J} l_{j}\left( X \right) In regression {argmax}_{y} \sum_{j=1}^{J} I\left( y=l_{j}\left( X \right) \right) In classification$$

Common choices for the loss function are *squared error loss* (for prediction) and *zero-one loss* (for classification). RF recursive feature elimination(Granitto *et al.*, 2006) (RF-RFE) is an extension of RF, which uses RFE to reduce the bias in the error estimations. Random survival forest(Ishwaran *et al.*, 2008) (RSF) is another extension of RF to analyze survival data (in the presence of right censoring) by introducing a new splitting rule and missing data imputation algorithm. Mixed-effects RF(Hajjem *et al.*, 2014) (MERF) is a generalization of RF for the clustered (hierarchical) data framework where samples are nested within specific groups. In this method, the RF has been used within the expectation-maximization(Dempster *et al.*, 1977) (EM) algorithm. Block Forests(Hornung and Wright, 2019) is another recent extension of RF for multi-Omics data integration purposes. Block forests consider the block structure (each Omics structure) via a modified split point selection of RF.

**BVS**(Mitchell and Beauchamp, 1988) is also an approach that simultaneously performs variable selection and prediction in the regression model. $L_{P}$-regularized estimations, such as lasso, ridge, and el-net can be considered as posterior Bayesian mode when placing an appropriate prior distribution for the regression coefficients. Lasso and ridge estimates can be considered as a posterior mode when the regression parameters ($\beta$) have an independent double exponential (Laplace) and independent normal priors, respectively(Jeffreys, 1998; Tibshirani, 1996). The regularization in BVS is commonly formulated via these shrinkage priors (so-called “scale-mixture"). For instance, in the Bayesian lasso, the scale mixture priors are represented as:

$\pi\left( \beta|\sigma^{2},\tau_{1},\ldots,\tau_{p} \right)=\pi\left( 0, \sigma^{2}D_{\tau} \right)$,

$$\pi\left( \tau_{1},\ldots,\tau_{p} \right)=\prod_{j=1}^{p} \frac{\lambda^{2}}{2}exp\left( -\lambda^{2}\tau_{j}^{2}/2 \right),$$

where $D_{\tau}=diag\left( \tau_{1}^{2},\ldots,\tau_{p}^{2} \right)$ and $\lambda$ indicates the tuning parameter. There are various extensions of BVS, including spatial BVS(Smith and Fahrmeir, 2007), Bayesian-lasso(Hans, 2009; Park and Casella, 2008), Bayesian graphical lasso(Wang, 2012) (Bgla), Bayesian fused lasso(Kyung *et al.*, 2010), Bayesian elastic-net, spatial BVS(Smith and Fahrmeir, 2007), hierarchical Bayesian hybrid Huberized support vector machine(Chakraborty and Guo, 2011) (HB-HHSVM), prior lasso(Wang *et al.*, 2013) (pLasso), expectation-maximization approach to BVS(Ročková and George, 2014) (EMVS), joint Bayesian variable and graph selection(Peterson *et al.*, 2016) (joint), and joint BVS and graph estimation(Sun *et al.*, 2020). Most of these extensions broaden the Bayesian approach and its connection with other variable selection methods by proposing new types of priors.

# References

Abdi H, Williams LJ, Connolly AC, Gobbini MI, Dunlop JP, Haxby JV. (2012). Multiple Subject Barycentric Discriminant Analysis (MUSUBADA): how to assign scans to categories without using spatial normalization. *Computational and Mathematical Methods in Medicine, 2012*.

Abid A, Zhang MJ, Bagaria VK, Zou J. (2018). Exploring patterns enriched in a dataset with contrastive principal component analysis. *Nature communications, 9*(1), 1-7.

Bastien P, Tenenhaus M. (2001). *PLS generalised linear regression. Application to the analysis of life time data.* Paper presented at the PLS and Related Methods, Proceedings of the PLS’01 International Symposium, CISIA-CERESTA, Paris.

Bastien P, Vinzi VE, Tenenhaus M. (2005). PLS generalised linear regression. *Computational Statistics & data analysis, 48*(1), 17-46.

Beale E, Kendall M, Mann D. (1967). The discarding of variables in multivariate analysis. *Biometrika, 54*(3-4), 357-366.

Bell AW, Deutsch EW, Au CE, Kearney RE, Beavis R, Sechi S*, et al.* (2009). A HUPO test sample study reveals common problems in mass spectrometry–based proteomics. *Nature methods, 6*(6), 423.

Bertsimas D, King A, Mazumder R. (2016). Best subset selection via a modern optimization lens. *The annals of statistics*, 813-852.

Bewicke-Copley F, Kumar EA, Palladino G, Korfi K, Wang J. (2019). Applications and analysis of targeted genomic sequencing in cancer studies. *Computational and structural biotechnology journal, 17*, 1348-1359.

Bischl B, Lang M, Kotthoff L, Schiffner J, Richter J, Studerus E*, et al.* (2016). mlr: Machine Learning in R. *The Journal of Machine Learning Research, 17*(1), 5938-5942.

Bishop CM. (1999). *Bayesian pca.* Paper presented at the Advances in neural information processing systems.

Blighe K, Lewis M, Lun A, Blighe MK. (2019). Package ‘PCAtools’.

Boileau P, Hejazi NS, Dudoit S. (2020a). Exploring high-dimensional biological data with sparse contrastive principal component analysis. *Bioinformatics, 36*(11), 3422-3430.

Boileau P, Hejazi NS, Dudoit S. (2020b). scPCA: A toolbox for sparse contrastive principal component analysis inR. *Journal of Open Source Software, 5*(46), 2079.

Bondell HD, Reich BJ. (2008). Simultaneous regression shrinkage, variable selection, and supervised clustering of predictors with OSCAR. *Biometrics, 64*(1), 115-123.

Boulesteix A-L, De Bin R, Jiang X, Fuchs M. (2017). IPF-LASSO: Integrative-penalized regression with penalty factors for prediction based on multi-omics data. *Computational and mathematical methods in medicine, 2017*.

Bouveyron C, Latouche P, Mattei P-A. (2018). Bayesian variable selection for globally sparse probabilistic PCA. *Electronic Journal of Statistics, 12*(2), 3036-3070.

Breheny P, Breheny MP. (2020). Package ‘grpreg’.

Breiman L. (2001). Random forests. *Machine learning, 45*(1), 5-32.

Brunet J-P, Tamayo P, Golub TR, Mesirov JP. (2004). Metagenes and molecular pattern discovery using matrix factorization. *Proceedings of the national academy of sciences, 101*(12), 4164-4169.

Bühlmann P, Rütimann P, van de Geer S, Zhang C-H. (2013). Correlated variables in regression: clustering and sparse estimation. *Journal of Statistical Planning and Inference, 143*(11), 1835-1858.

Bylesjö M, Rantalainen M, Nicholson JK, Holmes E, Trygg J. (2008). K-OPLS package: kernel-based orthogonal projections to latent structures for prediction and interpretation in feature space. *BMC bioinformatics, 9*(1), 106.

Cadima J, Jolliffe IT. (1995). Loading and correlations in the interpretation of principle compenents. *Journal of applied Statistics, 22*(2), 203-214.

Carroll JD. (1968). *Generalization of canonical correlation analysis to three or more sets of variables.* Paper presented at the Proceedings of the 76th annual convention of the American Psychological Association.

Casin P. (2001). A generalization of principal component analysis to K sets of variables. *Computational statistics & data analysis, 35*(4), 417-428.

Chakraborty S, Guo R. (2011). A Bayesian hybrid Huberized support vector machine and its applications in high-dimensional medical data. *Computational Statistics & Data Analysis, 55*(3), 1342-1356.

Champe PC, Harvey RA, Ferrier DR. (2005). *Biochemistry*: Lippincott Williams & Wilkins.

Chen J, Bushman FD, Lewis JD, Wu GD, Li H. (2013). Structure-constrained sparse canonical correlation analysis with an application to microbiome data analysis. *Biostatistics, 14*(2), 244-258.

Chen S, Kong H, Lu X, Li Y, Yin P, Zeng Z*, et al.* (2013). Pseudotargeted metabolomics method and its application in serum biomarker discovery for hepatocellular carcinoma based on ultra high-performance liquid chromatography/triple quadrupole mass spectrometry. *Analytical chemistry, 85*(17), 8326-8333.

Chen X-w, Jeong JC. (2007). *Enhanced recursive feature elimination.* Paper presented at the Sixth International Conference on Machine Learning and Applications (ICMLA 2007).

Chen X, Kim S, Lin Q, Carbonell JG, Xing EP. (2010). Graph-structured multi-task regression and an efficient optimization method for general fused lasso. *arXiv preprint arXiv:1005.3579*.

Chun H, Keleş S. (2010). Sparse partial least squares regression for simultaneous dimension reduction and variable selection. *Journal of the Royal Statistical Society: Series B (Statistical Methodology), 72*(1), 3-25.

Chung D, Chun H, Keles S. (2012). An Introduction to the ‘spls’ Package, Version 1.0. In: June.

Clark N, Wells K, Lindberg O. (2018). MRFcov: Markov Random Fields with additional covariates. R package version 1.0. In.

Consortia S. (2014). STATegRa: Classes and methods for multi-omics data integration. *R package, 1*, 1.

Consortium G. (2015). The Genotype-Tissue Expression (GTEx) pilot analysis: Multitissue gene regulation in humans. *Science, 348*(6235), 648-660.

Cox DR. (1972). Regression models and life‐tables. *Journal of the Royal Statistical Society: Series B (Methodological), 34*(2), 187-202.

Cruz-Cano R, Lee M-LT. (2014). Fast regularized canonical correlation analysis. *Computational Statistics & Data Analysis, 70*, 88-100.

Culhane AC, Thioulouse J, Perrière G, Higgins DG. (2005). MADE4: an R package for multivariate analysis of gene expression data. *Bioinformatics, 21*(11), 2789-2790.

De Bastiani F, Stasinopoulos M, Rigby R, De Bastiani MF. (2018). Package ‘gamlss. spatial’.

Dempster AP, Laird NM, Rubin DB. (1977). Maximum likelihood from incomplete data via the EM algorithm. *Journal of the Royal Statistical Society: Series B (Methodological), 39*(1), 1-22.

DeWitt PE, Bennett TD. (2019). ensr: R Package for Simultaneous Selection of Elastic Net Tuning Parameters. *arXiv preprint arXiv:1907.00914*.

Ding C, Peng H. (2005). Minimum redundancy feature selection from microarray gene expression data. *Journal of bioinformatics and computational biology, 3*(02), 185-205.

Dolédec S, Chessel D. (1994). Co‐inertia analysis: an alternative method for studying species–environment relationships. *Freshwater biology, 31*(3), 277-294.

Draper NR, Smith H. (1998). *Applied regression analysis* (Vol. 326): John Wiley & Sons.

Dray S, Chessel D, Thioulouse J. (2003). Co‐inertia analysis and the linking of ecological data tables. *Ecology, 84*(11), 3078-3089.

Dray S, Dufour A-B. (2007). The ade4 package: implementing the duality diagram for ecologists. *Journal of statistical software, 22*(4), 1-20.

Efron B, Hastie T, Johnstone I, Tibshirani R. (2004). Least angle regression. *The Annals of statistics, 32*(2), 407-499.

Efroymson M. (1966). Stepwise regression–a backward and forward look. *Florham Park, New Jersey*.

el Bouhaddani S, Uh H-W, Jongbloed G, Hayward C, Klarić L, Kiełbasa SM*, et al.* (2018). Integrating omics datasets with the OmicsPLS package. *BMC bioinformatics, 19*(1), 371.

Escofier B, Pages J. (1994). Multiple factor analysis (AFMULT package). *Computational statistics & data analysis, 18*(1), 121-140.

Escofier B, Pagès J. (1998). Analyses factorielles simples et multiples. *Dunod, Paris*.

Eswaran J, Horvath A, Godbole S, Reddy SD, Mudvari P, Ohshiro K*, et al.* (2013). RNA sequencing of cancer reveals novel splicing alterations. *Scientific reports, 3*, 1689.

Fiehn O, Kopka J, Dörmann P, Altmann T, Trethewey RN, Willmitzer L. (2000). Metabolite profiling for plant functional genomics. *Nature biotechnology, 18*(11), 1157-1161.

Friedman J, Hastie T, Tibshirani R. (2008). Sparse inverse covariance estimation with the graphical lasso. *Biostatistics, 9*(3), 432-441.

Friedman J, Hastie T, Tibshirani R. (2010a). A note on the group lasso and a sparse group lasso. *arXiv preprint arXiv:1001.0736*.

Friedman J, Hastie T, Tibshirani R. (2010b). Regularization paths for generalized linear models via coordinate descent. *Journal of statistical software, 33*(1), 1.

Garcia-Donato G, Forte A, Forte MA. (2015). Package ‘BayesVarSel’. *R Foundation for Statistical Computing, Vienna*.

Garthwaite PH. (1994). An interpretation of partial least squares. *Journal of the American Statistical Association, 89*(425), 122-127.

Gaujoux R, Seoighe C. (2010). A flexible R package for nonnegative matrix factorization. *BMC bioinformatics, 11*(1), 367.

Genz A, Bretz F, Miwa T, Mi X, Leisch F, Scheipl F*, et al.* (2020). Package ‘mvtnorm’. *Journal of Computational and Graphical Statistics, 11*, 950-971.

Gong J, Wan H, Mei S, Ruan H, Zhang Z, Liu C*, et al.* (2019). Pancan-meQTL: a database to systematically evaluate the effects of genetic variants on methylation in human cancer. *Nucleic acids research, 47*(D1), D1066-D1072.

González I, Déjean S, Martin P, Baccini A. (2008). CCA: An R package to extend canonical correlation analysis. *Journal of Statistical Software, 23*(12), 1-14.

Gower JC. (1966). Some distance properties of latent root and vector methods used in multivariate analysis. *Biometrika, 53*(3-4), 325-338.

Granitto PM, Furlanello C, Biasioli F, Gasperi F. (2006). Recursive feature elimination with random forest for PTR-MS analysis of agroindustrial products. *Chemometrics and Intelligent Laboratory Systems, 83*(2), 83-90.

Graves PR, Haystead TA. (2002). Molecular biologist's guide to proteomics. *Microbiology and molecular biology reviews, 66*(1), 39-63.

Griffiths JF, Griffiths AJ, Wessler SR, Lewontin RC, Gelbart WM, Suzuki DT*, et al.* (2005). *An introduction to genetic analysis*: Macmillan.

Guyon I, Weston J, Barnhill S, Vapnik V. (2002). Gene selection for cancer classification using support vector machines. *Machine learning, 46*(1-3), 389-422.

Hajjem A, Bellavance F, Larocque D. (2014). Mixed-effects random forest for clustered data. *Journal of Statistical Computation and Simulation, 84*(6), 1313-1328.

Hanafi M, Kohler A, Qannari E-M. (2011). Connections between multiple co-inertia analysis and consensus principal component analysis. *Chemometrics and intelligent laboratory systems, 106*(1), 37-40.

Hans C. (2009). Bayesian lasso regression. *Biometrika, 96*(4), 835-845.

Hans C. (2011). Elastic net regression modeling with the orthant normal prior. *Journal of the American Statistical Association, 106*(496), 1383-1393.

Hastie T, Tibshirani R, Tibshirani RJ. (2017). Extended comparisons of best subset selection, forward stepwise selection, and the lasso. *arXiv preprint arXiv:1707.08692*.

Hebbali A, Hebbali MA. (2017). Package ‘olsrr’.

Hocking RR, Leslie R. (1967). Selection of the best subset in regression analysis. *Technometrics, 9*(4), 531-540.

Hoerl AE, Kannard RW, Baldwin KF. (1975). Ridge regression: some simulations. *Communications in Statistics-Theory and Methods, 4*(2), 105-123.

Hornung R, Wright MN. (2019). Block Forests: random forests for blocks of clinical and omics covariate data. *BMC bioinformatics, 20*(1), 358.

Hotelling H. (1992). Relations between two sets of variates. In *Breakthroughs in statistics* (pp. 162-190): Springer.

Huang A, Liu D. (2016). EBglmnet: a comprehensive R package for sparse generalized linear regression models. *Bioinformatics*.

Huang X, Pan W. (2003). Linear regression and two-class classification with gene expression data. *Bioinformatics, 19*(16), 2072-2078.

Ishwaran H, Kogalur UB, Blackstone EH, Lauer MS. (2008). Random survival forests. *The annals of applied statistics, 2*(3), 841-860.

Ishwaran H, Kogalur UB, Kogalur MUB. (2020). Package ‘randomForestSRC’.

Jeffreys H. (1998). *The theory of probability*: OUP Oxford.

Jolliffe IT. (1986). Principal components in regression analysis. In *Principal component analysis* (pp. 129-155): Springer.

Jolliffe IT, Trendafilov NT, Uddin M. (2003). A modified principal component technique based on the LASSO. *Journal of computational and Graphical Statistics, 12*(3), 531-547.

Kassambara A, Mundt F. (2017). Package ‘factoextra’. *Extract and visualize the results of multivariate data analyses, 76*.

Kelly R, Weiss S, Levy B, Raby B, Lasky-Su J. (2018). Metabolite quantitative trait loci provide functional link between FADS2 and lung obstruction in asthmatics. In: Eur Respiratory Soc.

Klau S, Jurinovic V, Hornung R, Herold T, Boulesteix A-L. (2018). Priority-Lasso: a simple hierarchical approach to the prediction of clinical outcome using multi-omics data. *BMC bioinformatics, 19*(1), 322.

Kroonenberg PM. (2008). *Applied multiway data analysis* (Vol. 702): John Wiley & Sons.

Krzanowski W. (1984). Principal component analysis in the presence of group structure. *Journal of the Royal Statistical Society: Series C (Applied Statistics), 33*(2), 164-168.

Kuhn M. (2008). Building predictive models in R using the caret package. *Journal of statistical software, 28*(5), 1-26.

Kyung M, Gill J, Ghosh M, Casella G. (2010). Penalized regression, standard errors, and Bayesian lassos. *Bayesian Analysis, 5*(2), 369-411.

Labadie M, Vallin G, Petit A, Ring L, Hoffmann T, Gaston A*, et al.* (2020). Metabolite Quantitative Trait Loci for flavonoids provide new insights into the genetic architecture of strawberry (Fragaria x ananassa) fruit quality. *Journal of Agricultural and Food Chemistry*.

Lahti L, Huovilainen O-P, Lahti ML, DependencyModel CARAR, Matrix I. (2013). Package ‘dmt’.

Lê Cao K-A, Boitard S, Besse P. (2011). Sparse PLS discriminant analysis: biologically relevant feature selection and graphical displays for multiclass problems. *BMC Bioinformatics, 12*(1), 253. doi:10.1186/1471-2105-12-253

Lê Cao K-A, Martin PG, Robert-Granié C, Besse P. (2009). Sparse canonical methods for biological data integration: application to a cross-platform study. *BMC bioinformatics, 10*(1), 34.

Lê Cao K-A, Rossouw D, Robert-Granié C, Besse P. (2008). A sparse PLS for variable selection when integrating omics data. *Statistical applications in genetics and molecular biology, 7*(1).

Le Dien S, Pagès J. (2003). Hierarchical multiple factor analysis: application to the comparison of sensory profiles. *Food quality and preference, 14*(5-6), 397-403.

Lê S, Husson F. (2008). Sensominer: A package for sensory data analysis. *Journal of sensory studies, 23*(1), 14-25.

Lê S, Josse J, Husson F. (2008). FactoMineR: an R package for multivariate analysis. *Journal of statistical software, 25*(1), 1-18.

Lê S, Pagès J. (2010). Dmfa: Dual multiple factor analysis. *Communications in Statistics—Theory and Methods, 39*(3), 483-492.

Lee DD, Seung HS. (2001). *Algorithms for non-negative matrix factorization.* Paper presented at the Advances in neural information processing systems.

Lee J, Hastie T. (2013). *Structure learning of mixed graphical models.* Paper presented at the Artificial Intelligence and Statistics.

Legendre P, Legendre L. (2012). *Numerical ecology*: Elsevier.

Leurgans SE, Moyeed RA, Silverman BW. (1993). Canonical correlation analysis when the data are curves. *Journal of the Royal Statistical Society: Series B (Methodological), 55*(3), 725-740.

Li C, Li H. (2010). Variable selection and regression analysis for graph-structured covariates with an application to genomics. *The annals of applied statistics, 4*(3), 1498.

Li F, Zhang NR. (2010). Bayesian variable selection in structured high-dimensional covariate spaces with applications in genomics. *Journal of the American statistical association, 105*(491), 1202-1214.

Li W, Zhang S, Liu C-C, Zhou XJ. (2012). Identifying multi-layer gene regulatory modules from multi-dimensional genomic data. *Bioinformatics, 28*(19), 2458-2466.

Li Y, Jackson SA. (2015). Gene network reconstruction by integration of prior biological knowledge. *G3: Genes, Genomes, Genetics, 5*(6), 1075-1079.

Liaw A, Wiener M. (2002). Classification and regression by randomForest. *R news, 2*(3), 18-22.

Lin X, Boutros P. (2019). NNLM: A package For Fast And Versatile Nonnegative Matrix Factorization. In.

Liu X, Zhou L, Shi X, Xu G. (2019). New advances in analytical methods for mass spectrometry-based large-scale metabolomics study. *TrAC Trends in Analytical Chemistry, 121*, 115665.

Löfstedt T, Trygg J. (2011). OnPLS—a novel multiblock method for the modelling of predictive and orthogonal variation. *Journal of Chemometrics, 25*(8), 441-455.

Lumley T, Lumley MT. (2013). Package ‘leaps’. *Regression Subset Selection. Thomas Lumley Based on Fortran Code by Alan Miller. Available online:* [*http://CRAN*](http://cran)*. R-project. org/package= leaps (accessed on 18 March 2018)*.

Lykou A, Whittaker J. (2010). Sparse CCA using a Lasso with positivity constraints. *Computational Statistics & Data Analysis, 54*(12), 3144-3157.

Manzoni C, Kia DA, Vandrovcova J, Hardy J, Wood NW, Lewis PA*, et al.* (2018). Genome, transcriptome and proteome: the rise of omics data and their integration in biomedical sciences. *Briefings in bioinformatics, 19*(2), 286-302.

Meinshausen N. (2007). Relaxed lasso. *Computational Statistics & Data Analysis, 52*(1), 374-393.

Meng C, Culhane A, Gholami AM, Meng MC, BiocStyle S, Thioulouse J*, et al.* (2013). Package ‘omicade4’. *Journal of Statistical Software, 22*(4), 1-20.

Meng C, Kuster B, Culhane AC, Gholami AM. (2014). A multivariate approach to the integration of multi-omics datasets. *BMC bioinformatics, 15*(1), 162.

Meng C, Zeleznik OA, Thallinger GG, Kuster B, Gholami AM, Culhane AC. (2016). Dimension reduction techniques for the integrative analysis of multi-omics data. *Briefings in bioinformatics, 17*(4), 628-641.

Min EJ, Long Q. (2020). Sparse multiple co-Inertia analysis with application to integrative analysis of multi-Omics data. *BMC bioinformatics, 21*, 1-12.

Min EJ, Safo SE, Long Q. (2019). Penalized co-inertia analysis with applications to-omics data. *Bioinformatics, 35*(6), 1018-1025.

Mitchell TJ, Beauchamp JJ. (1988). Bayesian variable selection in linear regression. *Journal of the american statistical association, 83*(404), 1023-1032.

Mogensen UB, Ishwaran H, Gerds TA. (2012). Evaluating random forests for survival analysis using prediction error curves. *Journal of statistical software, 50*(11), 1.

Morand E, Pagès J. (2006). Procrustes multiple factor analysis to analyse the overall perception of food products. *Food quality and preference, 17*(1-2), 36-42.

Mundra PA, Rajapakse JC. (2007). *SVM-RFE with relevancy and redundancy criteria for gene selection.* Paper presented at the IAPR International Workshop on Pattern Recognition in Bioinformatics.

Nagalakshmi U, Waern K, Snyder M. (2010). RNA‐Seq: a method for comprehensive transcriptome analysis. *Current protocols in molecular biology, 89*(1), 4.11. 11-14.11. 13.

Nguyen DV, Rocke DM. (2002). Partial least squares proportional hazard regression for application to DNA microarray survival data. *Bioinformatics, 18*(12), 1625-1632.

Nygård S, Borgan Ø, Lingjærde OC, Størvold HL. (2008). Partial least squares Cox regression for genome-wide data. *Lifetime Data Analysis, 14*(2), 179-195.

Oksanen J, Kindt R, Legendre P, O’Hara B, Stevens MHH, Oksanen MJ*, et al.* (2007). The vegan package. *Community ecology package, 10*(631-637), 719.

Paradis E, Blomberg S, Bolker B, Brown J, Claude J, Cuong HS*, et al.* (2015). Package ‘ape’. *Analyses of phylogenetics and evolution, version, 2*, 4-1.

Park T, Casella G. (2008). The bayesian lasso. *Journal of the American Statistical Association, 103*(482), 681-686.

Peterson CB, Stingo FC, Vannucci M. (2016). Joint Bayesian variable and graph selection for regression models with network‐structured predictors. *Statistics in medicine, 35*(7), 1017-1031.

Price BS, Sherwood B. (2017). A cluster elastic net for multivariate regression. *The Journal of Machine Learning Research, 18*(1), 8685-8723.

Qian W, Yang Y. (2013). Model selection via standard error adjusted adaptive lasso. *Annals of the Institute of Statistical Mathematics, 65*(2), 295-318.

Rantalainen M, Bylesjö M, Cloarec O, Nicholson JK, Holmes E, Trygg J. (2007). Kernel‐based orthogonal projections to latent structures (K‐OPLS). *Journal of Chemometrics: A Journal of the Chemometrics Society, 21*(7‐9), 376-385.

Rapaport F, Khanin R, Liang Y, Pirun M, Krek A, Zumbo P*, et al.* (2013). Comprehensive evaluation of differential gene expression analysis methods for RNA-seq data. *Genome biology, 14*(9), 1-13.

Reinke SN, Galindo-Prieto B, Skotare T, Broadhurst DI, Singhania A, Horowitz D*, et al.* (2018). OnPLS-based multi-block data integration: a multivariate approach to interrogating biological interactions in asthma. *Analytical chemistry, 90*(22), 13400-13408.

Revelle W. (2011). An overview of the psych package. *Dep Psychol Northwest Univ, 3*, 1-25.

Revelle W, Revelle MW. (2015). Package ‘psych’. *The comprehensive R archive network*.

Ripley B, Venables B, Bates DM, Hornik K, Gebhardt A, Firth D*, et al.* (2013). Package ‘mass’. *Cran R, 538*.

Ročková V, George EI. (2014). EMVS: The EM approach to Bayesian variable selection. *Journal of the American Statistical Association, 109*(506), 828-846.

Rohart F, Gautier B, Singh A, Lê Cao K-A. (2017). mixOmics: An R package for ‘omics feature selection and multiple data integration. *PLoS computational biology, 13*(11), e1005752.

Romanoski CE, Lee S, Kim MJ, Ingram-Drake L, Plaisier CL, Yordanova R*, et al.* (2010). Systems genetics analysis of gene-by-environment interactions in human cells. *The American Journal of Human Genetics, 86*(3), 399-410.

Sankaran K, Holmes SP. (2019). Multitable methods for microbiome data integration. *Frontiers in genetics, 10*.

Schadt EE, Lamb J, Yang X, Zhu J, Edwards S, GuhaThakurta D*, et al.* (2005). An integrative genomics approach to infer causal associations between gene expression and disease. *Nature genetics, 37*(7), 710-717.

Scheipl F, Scheipl MF. (2020). Package ‘spikeSlabGAM’.

Shendure J. (2008). The beginning of the end for microarrays? *Nature methods, 5*(7), 585-587.

Sill M, Saadati M, Benner A. (2015). Applying stability selection to consistently estimate sparse principal components in high-dimensional molecular data. *Bioinformatics, 31*(16), 2683-2690.

Simon N, Friedman J, Hastie T, Tibshirani R. (2013). A sparse-group lasso. *Journal of computational and graphical statistics, 22*(2), 231-245.

Simon N, Friedman J, Hastie T, Tibshirani R, Simon MN. (2018). Package ‘SGL’. *CRAN Documentation*.

Šmilauer P, Košnar J, Kotilínek M, Šmilauerová M. (2020). Contrasting effects of host identity, plant community, and local species pool on the composition and colonization levels of arbuscular mycorrhizal fungal community in a temperate grassland. *New Phytologist, 225*(1), 461-473.

Smilde AK, Westerhuis JA, de Jong S. (2003). A framework for sequential multiblock component methods. *Journal of Chemometrics: A Journal of the Chemometrics Society, 17*(6), 323-337.

Smith AK, Kilaru V, Kocak M, Almli LM, Mercer KB, Ressler KJ*, et al.* (2014). Methylation quantitative trait loci (meQTLs) are consistently detected across ancestry, developmental stage, and tissue type. *BMC genomics, 15*(1), 145.

Smith M, Fahrmeir L. (2007). Spatial Bayesian variable selection with application to functional magnetic resonance imaging. *Journal of the American Statistical Association, 102*(478), 417-431.

Stacklies W, Redestig H, Scholz M, Walther D, Selbig J. (2007). pcaMethods—a bioconductor package providing PCA methods for incomplete data. *Bioinformatics, 23*(9), 1164-1167.

Stražar M, Žitnik M, Zupan B, Ule J, Curk T. (2016). Orthogonal matrix factorization enables integrative analysis of multiple RNA binding proteins. *Bioinformatics, 32*(10), 1527-1535.

Subramanian I, Verma S, Kumar S, Jere A, Anamika K. (2020). Multi-omics data integration, interpretation, and its application. *Bioinformatics and biology insights, 14*, 1177932219899051.

Sun BB, Maranville JC, Peters JE, Stacey D, Staley JR, Blackshaw J*, et al.* (2018). Genomic atlas of the human plasma proteome. *Nature, 558*(7708), 73-79.

Sun W, Chang C, Long Q. (2020). *Joint Bayesian Variable Selection and Graph Estimation for Non-linear SVM with Application to Genomics Data.* Paper presented at the 2020 IEEE 7th International Conference on Data Science and Advanced Analytics (DSAA).

Sun YV, Hu Y-J. (2016). Integrative analysis of multi-omics data for discovery and functional studies of complex human diseases. In *Advances in genetics* (Vol. 93, pp. 147-190): Elsevier.

Team RC. (2013). R: A language and environment for statistical computing. In: Vienna, Austria.

Tenenhaus A, Guillemot V, Tenenhaus MA. (2017). Package ‘RGCCA’.

Tenenhaus A, Tenenhaus M. (2011). Regularized generalized canonical correlation analysis. *Psychometrika, 76*(2), 257.

Tenenhaus M, Tenenhaus A, Groenen PJ. (2017). Regularized generalized canonical correlation analysis: a framework for sequential multiblock component methods. *Psychometrika, 82*(3), 737-777.

Ter Braak C, Juggins S, Birks H, Van der Voet H. (1993). Weighted averaging partial least squares regression (WA-PLS): definition and comparison with other methods for species-environment calibration. In *Multivariate environmental statistics* (pp. 525-560): Elsevier.

Tibshirani R. (1996). Regression shrinkage and selection via the lasso. *Journal of the Royal Statistical Society: Series B (Methodological), 58*(1), 267-288.

Tibshirani R, Saunders M, Rosset S, Zhu J, Knight K. (2005). Sparsity and smoothness via the fused lasso. *Journal of the Royal Statistical Society: Series B (Statistical Methodology), 67*(1), 91-108.

Timp W, Timp G. (2020). Beyond mass spectrometry, the next step in proteomics. *Science Advances, 6*(2), eaax8978.

Tipping ME, Bishop CM. (1999). Probabilistic principal component analysis. *Journal of the Royal Statistical Society: Series B (Statistical Methodology), 61*(3), 611-622.

Trygg J. (2002). O2‐PLS for qualitative and quantitative analysis in multivariate calibration. *Journal of Chemometrics: A Journal of the Chemometrics Society, 16*(6), 283-293.

Trygg J, Wold S. (2002). Orthogonal projections to latent structures (O‐PLS). *Journal of Chemometrics: A Journal of the Chemometrics Society, 16*(3), 119-128.

Tu Y, Hung YS, Hu L, Zhang Z. (2015). *PCA-SIR: A new nonlinear supervised dimension reduction method with application to pain prediction from EEG.* Paper presented at the 2015 7th International IEEE/EMBS Conference on Neural Engineering (NER).

Tutz G, Ulbricht J. (2009). Penalized regression with correlation-based penalty. *Statistics and Computing, 19*(3), 239-253.

Ullah MI, Aslam M, Altaf S. (2018). lmridge: A Comprehensive R Package for Ridge Regression. *R J., 10*(2), 326.

Vallarino JG, Pott DM, Cruz-Rus E, Miranda L, Medina-Minguez JJ, Valpuesta V*, et al.* (2019). Identification of quantitative trait loci and candidate genes for primary metabolite content in strawberry fruit. *Horticulture Research, 6*(1), 1-17.

Van De Wiel MA, Lien TG, Verlaat W, van Wieringen WN, Wilting SM. (2016). Better prediction by use of co‐data: adaptive group‐regularized ridge regression. *Statistics in Medicine, 35*(3), 368-381.

Waaijenborg S, de Witt Hamer PCV, Zwinderman AH. (2008). Quantifying the association between gene expressions and DNA-markers by penalized canonical correlation analysis. *Statistical applications in genetics and molecular biology, 7*(1).

Wang H. (2012). Bayesian graphical lasso models and efficient posterior computation. *Bayesian Analysis, 7*(4), 867-886.

Wang Z, Xu W, San Lucas FA, Liu Y. (2013). Incorporating prior knowledge into gene network study. *Bioinformatics, 29*(20), 2633-2640.

Wangen L, Kowalski B. (1989). A multiblock partial least squares algorithm for investigating complex chemical systems. *Journal of chemometrics, 3*(1), 3-20.

Wehrens R, Mevik B-H. (2007). The pls package: principal component and partial least squares regression in R.

Westerhuis JA, Kourti T, MacGregor JF. (1998). Analysis of multiblock and hierarchical PCA and PLS models. *Journal of Chemometrics: A Journal of the Chemometrics Society, 12*(5), 301-321.

Witten D, Tibshirani R, Gross S, Narasimhan B, Witten MD. (2020). Package ‘pma’. *Genetics and Molecular Biology, 8*(1), 28.

Witten DM, Tibshirani R. (2009). Covariance‐regularized regression and classification for high dimensional problems. *Journal of the Royal Statistical Society: Series B (Statistical Methodology), 71*(3), 615-636.

Witten DM, Tibshirani R, Hastie T. (2009). A penalized matrix decomposition, with applications to sparse principal components and canonical correlation analysis. *Biostatistics, 10*(3), 515-534.

Wold S. (1987). *PLS modeling with latent variables in two or more dimensions*: Verlag nicht ermittelbar.

Wold S, Antti H, Lindgren F, Öhman J. (1998). Orthogonal signal correction of near-infrared spectra. *Chemometrics and Intelligent laboratory systems, 44*(1-2), 175-185.

Wold S, Kettaneh N, Tjessem K. (1996). Hierarchical multiblock PLS and PC models for easier model interpretation and as an alternative to variable selection. *Journal of chemometrics, 10*(5‐6), 463-482.

Wold S, Martens H, Wold H. (1983). The multivariate calibration problem in chemistry solved by the PLS method. In *Matrix pencils* (pp. 286-293): Springer.

Woo J, Winterhoff BJ, Starr TK, Aliferis C, Wang J. (2019). De novo prediction of cell-type complexity in single-cell RNA-seq and tumor microenvironments. *Life science alliance, 2*(4).

Wood S, Wood MS. (2015). Package ‘mgcv’. *R package version, 1*, 29.

Yang Z, Michailidis G. (2016). A non-negative matrix factorization method for detecting modules in heterogeneous omics multi-modal data. *Bioinformatics, 32*(1), 1-8.

Yang Z, Zheng R, Gao Y, Zhang Q, Zhang H. (2014). Abnormal gene expression and gene fusion in lung adenocarcinoma with high-throughput RNA sequencing. *Cancer gene therapy, 21*(2), 74-82.

Yoon S, Kim S. (2009). Mutual information-based SVM-RFE for diagnostic classification of digitized mammograms. *Pattern Recognition Letters, 30*(16), 1489-1495.

Yuan M, Lin Y. (2006). Model selection and estimation in regression with grouped variables. *Journal of the Royal Statistical Society: Series B (Statistical Methodology), 68*(1), 49-67.

Zass R, Shashua A. (2006). Nonnegative sparse PCA. *Advances in neural information processing systems, 19*, 1561-1568.

Zhang S, Liu C-C, Li W, Shen H, Laird PW, Zhou XJ. (2012). Discovery of multi-dimensional modules by integrative analysis of cancer genomic data. *Nucleic acids research, 40*(19), 9379-9391.

Zhao Z, Banterle M, Bottolo L, Richardson S, Lewin A, Zucknick M. BayesSUR: An R package for high-dimensional multivariate Bayesian variable and covariance selection in linear regression.

Zou H. (2006). The adaptive lasso and its oracle properties. *Journal of the American statistical association, 101*(476), 1418-1429.

Zou H, Hastie T. (2005). Regularization and variable selection via the elastic net. *Journal of the royal statistical society: series B (statistical methodology), 67*(2), 301-320.

Zou H, Hastie T, Tibshirani R. (2006). Sparse principal component analysis. *Journal of computational and graphical statistics, 15*(2), 265-286.

Zuur A, Ieno EN, Smith GM. (2007). *Analyzing ecological data*: Springer.
